# Phylogenomic analyses of Sapindales support new family relationships, rapid Mid-Cretaceous Hothouse diversification, and heterogeneous histories of gene duplication

**DOI:** 10.3389/fpls.2023.1063174

**Published:** 2023-03-07

**Authors:** Elizabeth M. Joyce, Marc S. Appelhans, Sven Buerki, Martin Cheek, Jurriaan M. de Vos, José R. Pirani, Alexandre R. Zuntini, Julien B. Bachelier, Michael J. Bayly, Martin W. Callmander, Marcelo F. Devecchi, Susan K. Pell, Milton Groppo, Porter P. Lowry, John Mitchell, Carolina M. Siniscalchi, Jérôme Munzinger, Harvey K. Orel, Caroline M. Pannell, Lars Nauheimer, Hervé Sauquet, Andrea Weeks, Alexandra N. Muellner-Riehl, Ilia J. Leitch, Olivier Maurin, Félix Forest, Katharina Nargar, Kevin R. Thiele, William J. Baker, Darren M. Crayn

**Affiliations:** ^1^ Systematics, Biodiversity and Evolution of Plants, Ludwig-Maximilians-Universität München, Munich, Germany; ^2^ College of Science and Engineering, James Cook University, Cairns, QLD, Australia; ^3^ Australian Tropical Herbarium, James Cook University, Cairns, QLD, Australia; ^4^ Department of Systematics, Biodiversity and Evolution of Plants, University of Göttingen, Goettingen, Germany; ^5^ Department of Botany, National Museum of Natural History, Smithsonian Institution, Washington, DC, United States; ^6^ Department of Biological Sciences, Boise State University, Boise, ID, United States; ^7^ Royal Botanic Gardens, Kew, Richmond, United Kingdom; ^8^ Department of Environmental Sciences, University Basel, Basel, Switzerland; ^9^ Departamento de Botaênica, Universidade de Saão Paulo, Herbário SPF, Saão Paulo, Brazil; ^10^ Institut für Biologie, Freie Universität Berlin, Berlin, Germany; ^11^ School of BioSciences, The University of Melbourne, Parkville, VIC, Australia; ^12^ Conservatoire et Jardin botaniques de la Ville de Genève, Geneva, Switzerland; ^13^ United States Botanic Garden, Washington, DC, United States; ^14^ Missouri Botanical Garden, St. Louis, MO, United States; ^15^ Institut de Systématique, Évolution, et Biodiversité (ISYEB), Muséum National d’Histoire Naturelle, Centre National de la Recherche Scientifique, Sorbonne Université, École Pratique des Hautes Études, Université des Antilles, Paris, France; ^16^ New York Botanical Garden, New York, NY, United States; ^17^ Department of Biological Sciences, Harned Hall, Mississippi State University, Mississippi State, MS, United States; ^18^ AMAP, Université Montpellier, Institut de Recherche pour le Développement (IRD), Centre de coopération internationale en recherche agronomique pour le développement (CIRAD), Centre National de la Recherche Scientifique (CNRS), Institut national de la recherche agronomique (INRAE), Montpellier, France; ^19^ Department of Biology, Oxford University, Oxford, United Kingdom; ^20^ Marine Laboratory, Queen’s University Belfast, Portaferry, United Kingdom; ^21^ National Herbarium of New South Wales (NSW), Royal Botanic Gardens and Domain Trust, Sydney, NSW, Australia; ^22^ Department of Biology, George Mason University, Fairfax, VA, United States; ^23^ Department of Molecular Evolution and Plant Systematics & Herbarium, Faculty of Life Sciences, University of Leipzig, Leipzig, Germany; ^24^ German Centre for Integrative Biodiversity Research (iDiv) Halle-Jena-Leipzig, Leipzig, Germany; ^25^ National Research Collections Australia, Commonwealth Industrial and Scientific Research Organization (CSIRO), Canberra, ACT, Australia; ^26^ School of Biological Sciences, University of Western Australia, Perth, WA, Australia

**Keywords:** Cenomanian-Turonian Thermal Maximum, phylogenomics, target enrichment, sequence capture, HybSeq, paralogy

## Abstract

Sapindales is an angiosperm order of high economic and ecological value comprising nine families, c. 479 genera, and c. 6570 species. However, family and subfamily relationships in Sapindales remain unclear, making reconstruction of the order’s spatio-temporal and morphological evolution difficult. In this study, we used Angiosperms353 target capture data to generate the most densely sampled phylogenetic trees of Sapindales to date, with 448 samples and c. 85% of genera represented. The percentage of paralogous loci and allele divergence was characterized across the phylogeny, which was time-calibrated using 29 rigorously assessed fossil calibrations. All families were supported as monophyletic. Two core family clades subdivide the order, the first comprising Kirkiaceae, Burseraceae, and Anacardiaceae, the second comprising Simaroubaceae, Meliaceae, and Rutaceae. Kirkiaceae is sister to Burseraceae and Anacardiaceae, and, contrary to current understanding, Simaroubaceae is sister to Meliaceae and Rutaceae. Sapindaceae is placed with Nitrariaceae and Biebersteiniaceae as sister to the core Sapindales families, but the relationships between these families remain unclear, likely due to their rapid and ancient diversification. Sapindales families emerged in rapid succession, coincident with the climatic change of the Mid-Cretaceous Hothouse event. Subfamily and tribal relationships within the major families need revision, particularly in Sapindaceae, Rutaceae and Meliaceae. Much of the difficulty in reconstructing relationships at this level may be caused by the prevalence of paralogous loci, particularly in Meliaceae and Rutaceae, that are likely indicative of ancient gene duplication events such as hybridization and polyploidization playing a role in the evolutionary history of these families. This study provides key insights into factors that may affect phylogenetic reconstructions in Sapindales across multiple scales, and provides a state-of-the-art phylogenetic framework for further research.

## Introduction

Sapindales is a flowering plant order of great biological and economic importance; it includes c. 2% of the world’s angiosperm diversity, and in 2021, raw products from its taxa were estimated to be worth more than US$31 billion p.a. (c. 0.2% of the world trade market; Freiberg et al., 2020 onwards; Stevens, 2001; Simoes and Hidalgo, 2011; Simoes and Hidalgo, 2022 onwards). Sapindales currently includes six medium-sized to large families (Anacardiaceae, Burseraceae, Meliaceae, Rutaceae, Sapindaceae, and Simaroubaceae, all with >150 species) and three small families (Nitrariaceae, and the monogeneric Biebersteiniaceae and Kirkiaceae), with c. 479 genera and c. 6750 species. Its species are predominantly tropical woody plants with pinnately compound leaves and small, tetra- or pentamerous flowers with intrastaminal nectar disks. However, remarkable morphological and ecological diversity exists within Sapindales, with species presenting as herbs, lianas, shrubs, trees and mangroves that inhabit tropical, arid, coastal, or montane environments. Taxa such as mangoes, citrus, mahoganies, cashews, maples, pistachio, lychee, frankincense, and myrrh are important to agricultural, pharmaceutical, cosmetic, chemical, and timber industries, and contribute to the high economic value of the order.

Despite its biological and economic significance, Sapindales has a complex taxonomic history, and relationships among families within the order are uncertain. From the nineteenth century, families now placed in Sapindales were variously assigned to 25 different orders. In the 20th Century, two main ordinal concepts persisted, with Wettstein (1901) and Cronquist (1968) both recognizing an expanded order including Rutales + Sapindales, and Takhtajan (2009) assigning families to separate orders (i.e., Sapindales, Rutales and Zygophyllales). More recently, molecular studies supported the expanded ordinal concept, suggesting that Anacardiaceae, Biebersteiniaceae, Burseraceae, Kirkiaceae, Nitrariaceae, Meliaceae, Rutaceae, Sapindaceae, and Simaroubaceae form a monophyletic clade distinct from Zygophyllales (Chase et al., 1993; Gadek et al., 1996; Muellner et al., 2007; Muellner-Riehl et al., 2016; Li et al., 2019; Li et al., 2021). The inclusion of these nine families in Sapindales is now generally accepted (Kubitzki, 2011; APG, 2016). Angiosperm-wide molecular studies differ in their placement of Sapindales within the rosids, but most studies suggest Sapindales is most closely related to Malvales, Brassicales, Huerteales, and Picramniales (Chase et al., 1993; APG, 2016; Li et al., 2019; Ramírez-Barahona et al., 2020; Li et al., 2021; Baker et al., 2022).

Despite extensive systematic research on the order, the relationships of most families within Sapindales remain uncertain (Gadek et al., 1996; Stevens, 2001; Muellner et al., 2007; Wang et al., 2009; Muellner-Riehl et al., 2016; Lin et al., 2018; Li et al., 2019). A close relationship of Burseraceae and Anacardiaceae is well-established, with both families previously included in the family Terebinthaceae and sharing the synapomorphies of vertical intercellular secretory canals in the primary and secondary phloem and the ability to synthesise biflavonyls (Wannan et al., 1985; Wannan, 1986; Wannan and Quinn, 1990; Wannan and Quinn, 1991; Terrazas, 1994). More recent molecular studies support Anacardiaceae and Burseraceae as monophyletic, although infra-familial classifications are in need of revision (Gadek et al., 1996; Pell, 2004; Weeks et al., 2014; Muellner-Riehl et al., 2016; Mitchell et al., 2022). Morphological studies have shown a close affinity in floral structure of members of the monogeneric family Kirkiaceae to Anacardiaceae and Burseraceae (Bachelier and Endress, 2008), and, together, these families form a moderately to well-supported clade in recent molecular studies (Muellner et al., 2007; Muellner-Riehl et al., 2016; Li et al., 2021). However, the relationships of Biebersteiniaceae and Nitrariaceae to the rest of the order, the position of Sapindaceae, and the relationships between Rutaceae, Meliaceae, and Simaroubaceae remain less clear. Nitrariaceae and Biebersteiniaceae are usually retrieved sequentially as sister to the other families in the order (Muellner et al., 2007; Appelhans et al., 2012; Muellner-Riehl et al., 2016; Li et al., 2019; Li et al., 2021), but Ramírez-Barahona et al. (2020) placed them together as sister to a clade comprising Kirkiaceae, Burseraceae, and Anacardiaceae. Regardless of their position within the order, the node between Nitrariaceae and Biebersteiniaceae has remained unsupported in multiple studies (Muellner-Riehl et al., 2016; Li et al., 2019; Ramírez-Barahona et al., 2020; Li et al., 2021). The position of Sapindaceae within the order also remains unresolved, being variously reconstructed as sister to a clade containing Rutaceae, Simaroubaceae, and Meliaceae (Appelhans et al., 2012; Muellner-Riehl et al., 2016; Li et al., 2019; Ramírez-Barahona et al., 2020; Li et al., 2021), as sister to Anacardiaceae and Burseraceae (Gadek et al., 1996; Lin et al., 2018), as sister to Anacardiaceae (Chase et al., 1993), or as a clade in a polytomy (Muellner et al., 2007); in all cases, the family relationships of Sapindaceae are poorly supported. Finally, the consensus of morphological and molecular evidence indicates that Rutaceae, Simaroubaceae, and Meliaceae form a clade within Sapindales; however, the relationships between these three families remain unclear. Many studies have found high support for Meliaceae being sister to Simaroubaceae and Rutaceae (Gadek et al., 1996; Lin et al., 2018; Li et al., 2019; Ramírez-Barahona et al., 2020; Li et al., 2021), but strong contradictory evidence suggests Rutaceae is sister to Simaroubaceae and Meliaceae (Muellner et al., 2007; Appelhans et al., 2012; Muellner-Riehl et al., 2016).

Resolution of family relationships within Sapindales is critical for understanding the evolutionary history of the order. This is particularly pertinent for understanding the development and evolution of unique traits with ecological and commercial significance such as wood characters (e.g. Pace et al., 2022), secondary metabolite synthesis (e.g. Fernandes da Silva et al., 2022), flower morphology (e.g. Bachelier and Endress, 2008; Bachelier et al., 2011; Alves et al., 2022), pollen morphology (e.g. Gonçalves-Esteves et al., 2022), secretory structures (e.g. Tölke et al., 2022), and cuticular chemical composition (e.g. Roma and Santos, 2022). Furthermore, an understanding of the evolution of the vast variation in nuclear DNA organization and ploidy levels within the order requires a robust, detailed phylogenetic framework (Pennington and Styles, 1975; Guimarães and Forni-Martins, 2022). Likewise, the spatio-temporal origins of the order can only be understood once relationships within it have been resolved. Differences in tree topology have also likely contributed to discrepancies in divergence ages estimated for families in previous phylogenetic work, with some studies reporting a Cretaceous origin for most Sapindales families (stem and crown nodes; Muellner-Riehl et al., 2016; Ramírez-Barahona et al., 2020), but others retrieving a Cenozoic origin for families (Li et al., 2019).

Recent advances in high-throughput sequencing methods provide new opportunities for resolving the familial relationships within Sapindales. Target capture sequencing has become the foremost high-throughput sequencing method for phylogenomics, enabling the reliable retrieval of hundreds or thousands of target loci at an increasingly affordable price (Cronn et al., 2012; Barrett et al., 2016; Bragg et al., 2016). The amount of data generated with target capture sequencing in combination with the development of universal bait kits such as Angiosperms353 (Johnson et al., 2019) has facilitated global efforts to resolve relationships of plants across multiple taxonomic scales (Baker et al., 2021; McDonnell et al., 2021; Baker et al., 2022). In addition, unlike historical Sanger approaches, the sequencing of a high number of reads in target capture approaches allows for the detection and handling of paralogous genes. Paralogous genes are genes with multiple copies that are the product of duplication of an ancestral gene (either by duplication of part of the genome, or of the whole genome) (Fitch, 1970). Duplication of genes can also be produced in the process of allopolyploidization, whereby the hybridization of two species results in the doubling of the genome; as these gene copies do not share a common ancestor they are technically called homeologs, but for the purposes of this paper we do not distinguish between paralogs and homeologs and refer to all loci with multiple copies resulting from duplication as paralogous. Angiosperm genomes often contain a large number of paralogous genes due to the prevalence of polyploidy or whole-genome duplication events in the evolutionary history of plants (Soltis et al., 2009; Jiao et al., 2011; Panchy et al., 2016). Although target capture bait kits such as Angiosperms353 are designed to target low- or single-copy loci, paralogous copies of targeted loci are present in many lineages (Nauheimer et al., 2021; Smith and Hahn, 2021; Ufimov et al., 2022). Paralogous loci can violate the assumption of homology in phylogenetic analysis and confound resulting trees, and so are commonly removed in analyses (e.g. Jones et al., 2019; Larridon et al., 2020). However, the retention and identification of paralogous loci in phylogenetic studies has been shown to be highly valuable for maximizing the amount of informative data, explaining discordance between gene trees, and in pinpointing where genome duplication events such as ancient polyploidization and hybridization have played an important role in the evolution of lineages (e.g. Nauheimer et al., 2021; Morales-Briones et al., 2021; Smith and Hahn, 2021; Ufimov et al., 2022). Identification, characterization and analysis of paralogy is now possible with target capture sequencing, making it a promising method for improving the resolution of relationships within Sapindales, and for gaining new insight into the role of gene duplication events during the evolution of the order.

In this study, we have achieved the most comprehensive sampling of Sapindales species in a phylogenetic study to date, and use target capture sequencing with the Angiosperms353 bait kit to infer family and subfamily relationships. We characterize patterns of paralogy across the order to investigate whether gene duplication events (whether through hybridization, autopolyploidization, or local gene duplications) have played a role in the evolution of Sapindales lineages and may explain any topological uncertainty. Finally, we go on to infer the temporal evolution of Sapindales, identifying key periods for the evolution of the order and assessing how these change when different crown ages for the angiosperms are assumed. The resulting phylogeny aims to improve our understanding of the order’s evolutionary history, and to serve as a robust framework for future phylogenetic, morphological, taxonomic, and systematic studies.

## Methods

### Sampling

A total of 472 samples were obtained for this analysis, including 448 representatives of Sapindales from all nine families and encompassing c. 85% of genera in the order (**Supplementary Material 1**). Generally, one sample per genus was included (where possible, the type species for the genus); multiple species were sampled for genera that were suspected to be polyphyletic based on previous studies and expert opinion. The outgroup comprised 24 samples from across the Pentapetalae, from the orders Brassicales, Crossosomatales, Ericales, Fabales, Geraniales, Huerteales, Malvales, and Myrtales (**Supplementary Material 1**). Data for 287 samples were newly generated for this study, sourced from the living collections of the Royal Botanic Gardens, Kew, silica gel-dried field collections, herbarium specimens from multiple institutions, and the DNA banks of the Royal Botanic Gardens, Kew, Australian Tropical Herbarium, United States Botanical Gardens, and Göttingen University (**Supplementary Material 1**). The dataset was augmented with Angiosperms353 data for 132 species produced for the Sapindaceae phylogeny of Buerki et al. (2021) and with data for 53 species downloaded from the Sequence Read Archive (SRA; NCBI, https://www.ncbi.nlm.nih.gov/sra, **Supplementary Material 1**).

### DNA extraction and quality control

For the new data generated in this study, DNA was extracted from silica gel-dried and herbarium samples using the CTAB protocol of Doyle and Doyle (1987). The protocol was modified at the isopropanol precipitation step, with samples left to precipitate at -20°C degrees over 24 hours for silica-dried and fresh samples, and a minimum of 72 hours for herbarium samples. Extractions were cleaned using Agencourt AMPureXP beads (Beckman Coulter, Indianapolis, USA) according to the manufacturer’s protocol and eluted to 50 µL. DNA quality and quantity was ascertained using a NanoDrop 1000 Spectrophotometer (Thermo Fisher Scientific, Massachusetts, USA) and Quantus Fluorometer (Promega Corporation, Wisconsin, USA), and average fragment size assessed visually after electrophoresis on a 1% agarose gel. For extractions with a concentration of less than 4 ng/µL, yield was increased by combining additional DNA extractions from the same sample and concentrating using a vacuum centrifuge.

### Library preparation and sequencing

Library preparation protocols varied depending on DNA quality. For higher-quality extractions (i.e., average fragment size > 350 bp), DNA was sonicated in an M220 Focused-ultrasonicator with microTUBES AFA Fiber Pre-slit Snap-caps (Covaris, Massachusetts, USA) following the manufacturer’s protocol. Shearing time varied from 30–90 seconds depending on DNA fragment size profile to obtain an average fragment size of 350 bp. Highly degraded samples with an average fragment size <350 bp were not sonicated. Sonicated samples were diluted to 200 ng DNA in 50 µL Tris, and non-sonicated samples to 100 ng DNA in 25 µL Tris.

Dual-indexed libraries were prepared using the NEBNext Ultra II Library Preparation Kit and the NEBNext Multiplex Oligos for Illumina (New England BioLabs, Massachusetts, USA) using half the manufacturer’s recommended volumes. Library size profiles were evaluated on a 4200 TapeStation System using High Sensitivity D1000 ScreenTapes (Agilent Technologies, California, USA), and library concentrations ascertained using a Quantus Fluorometer (Promega Corporation, Wisconsin, USA). All libraries were of an average fragment size of approximately 500 bp (including adapters). For libraries not meeting these standards, PCR, adaptor cleanup and/or size selection steps of the library preparation protocol were repeated. All libraries were normalized to a concentration of 10 nM and combined in 7.5 µL library pools with 20–24 samples per pool of similar fragment lengths.

Pooled libraries were enriched using the myBaits ‘Angiosperms 353 v1’ Target Sequence Capture Kit (Arbor Bioscience, Michigan, USA) following the manufacturer’s protocol. Hybridizations were performed at 60 or 65°C (depending on average fragment length) for 24 hours in a Hybex Microsample Incubator (SciGene, California, USA) using the same volume as the hybridization reaction volume (usually 30 µL) of red Chill-out Liquid Wax (Bio224 Rad, Hercules, CA, USA) to prevent evaporation.

Enriched library pools were amplified with KAPA HiFi 2X HotStart ReadyMix PCR Kit (Roche, Basel, Switzerland) for 14 PCR cycles, and subsequently cleaned using Agencourt AMPure XP Beads. Concentrations of pools were quantified with a Quantus Fluorometer and quality and size profiling were conducted on a 4200 TapeStation System using High Sensitivity D1000 ScreenTapes (Agilent Technologies, California, USA). The hybridised pools were then combined into sequencing runs of approximately 96 libraries in 30 µL at 6 nM concentration. Library pools were multiplexed and sequenced by Macrogen (Macrogen Inc., Seoul, South Korea) on an Illumina HiSeq (Illumina Inc., California, USA) producing 2x 150 bp paired-end reads.

### Gene retrieval

Trimmomatic was used to remove adapter sequences, poor-quality base calls and poor-quality reads from sequencing reads with the settings: illuminaclip 2:30:10, leading 30, trailing 30, sliding window 4:2:30, minimum length 36 and Phred-33 base quality encoding (Bolger et al., 2014). Exon sequences were assembled using the HybPiper v1 pipeline for nucleotide data (Johnson et al., 2016). Trimmed reads were mapped against the Sapindales subset of the mega target file, which led to a substantially higher recovery for Sapindales than with the standard Angiosperms353 target file (McLay et al., 2021). Exons and supercontigs were retrieved using the HybPiper script retrieve_sequences.py, and summary gene recovery statistics for each sample were generated for each sample with the HybPiper scripts get_seq_lengths.py and hybpiper_stats.py.

### Data cleaning and paralogy characterization

We cleaned loci or samples where the multiple copies are uninformative (i.e., are likely due to contamination or laboratory error), and retained paralogous loci where the cause is more likely to be gene duplication events. This enabled maximum retention of data, and the identification of lineages in the phylogenetic tree where gene duplication events (such as polyploidization or hybridization) could have played a role. It is uncertain whether Angiosperms353 baits differ in their ability to hybridize with paralogous copies of genes across lineages at the ordinal scale, and theoretically, this could result in an underestimation of paralogy in cases where gene copies are highly divergent or lost. Nevertheless, we suggest that the characterization of paralogy is useful for gaining a general understanding of where genes have been duplicated and retained in Sapindales, how these patterns may differ across the order, and where gene duplication events may affect evolutionary inference from phylogenetic trees and morphology.

To detect, clean and characterize paralogy in our Sapindales target capture data, we used HybPhaser v1 (Nauheimer et al., 2021). HybPhaser v1 uses reference mapping and codes any discrepancies as ambiguity characters. This process enables the identification of single nucleotide polymorphisms (SNPs), which facilitates the characterization of signals of paralogy across the phylogenetic tree (whereby a high number of SNPs compared to related lineages can indicate the presence of multiple gene copies), allows the cleaning of sequences and samples with extremely high signals of paralogy (that are more likely to be due to contamination), and enables reconciliation of polymorphic sites as ambiguities for phylogenetic analysis (rather than consensus bases that may be called from the most common copy of a gene for any given locus). It is possible that the use of ambiguities in sequences could depress branch lengths relative to the use of a tree estimated with consensus sequences; however, we consider the use of ambiguities to be the most conservative and accurate way to code SNPs from paralogous loci, as it avoids the analysis of chimeric contigs assembled from reads of paralogous loci as orthologous loci, and places more weight on the regions of the locus that are conserved across copies within the same sample in the phylogenetic analysis.

Reads were remapped to the contig for each gene generated with the HybPhaser script Generate_consensus_sequences.sh. Information on length and coverage of sequences from all samples and loci was collated with the HybPhaser script Rscript_1a_count_snps_in_consensus_seqs.R. Following visual inspection of the outputs which summarised heterozygosity in the raw Angioperms353 data and manual inspection of the remapped.bam files, HybPhaser scripts R1b_optimize_dataset.R and Configure_1_SNPs_assessment.R were used to clean the data by removing samples with >50% missing loci and loci with <30% locus recovery, >21% missing samples, as well as samples and loci with outlying heterozygosity (> 1.5x the inter-quartile range for heterozygosity, which we considered more likely to be contaminated). Tables of heterozygosity and allele divergence were collated with the script Rscript_1c_summary_table.R, and the cleaned consensus sequences with ambiguities exported with Rscript_1d_generate_sequence_lists.R. A summary of final sample coverage, sequence length, heterozygosity, and allele divergence after cleaning is given in **Supplementary Material 2**.

We examined SNP patterns in the cleaned Angiosperms353 dataset and determined the proportion of heterozygous loci and the proportion of loci with more than 0%, 0.5%, 1%, and 2% SNPs, as well as mean allele divergence for each sample. While heterozygosity (as indicated by the presence of SNPs, i.e., any locus with > 0% SNPs) can be expected in any homologous locus due to allelic variation, loci with a high proportion of SNPs (e.g., >1% SNPs per locus) more likely result from multiple gene copies. Therefore, we consider that any locus with >1% SNPs in the cleaned dataset is likely to be paralogous, with paralogy caused by biological processes such as gene duplication, polyploidization, and hybridization, rather than by allelic variation, sequencing error or contamination.

### Phylogenomic tree construction

HybPhaser consensus sequences (i.e. sequences including ambiguity codes) were aligned using MAFFT with the -auto flag to automatically select alignment strategy (Katoh and Standley, 2013). Sites with >75% missing data were removed from the alignment using the –clean option in Phyutility (Smith and Dunn, 2008), and exon alignments concatenated with AMAS following visual inspection (Borowiec, 2016). A maximum likelihood concatenated tree was then estimated from the clean alignment in IQ-TREE (Nguyen et al., 2015), with the appropriate substitution model and partitioning scheme for the alignment chosen using ModelFinder Plus option –MFP+MERGE and 1000 ultrafast bootstrap replicates to determine bootstrap support (BS; Lanfear et al., 2012; Nguyen et al., 2015; Kalyaanamoorthy et al., 2017; Hoang et al., 2018). To generate a coalescent species tree, gene trees were estimated from cleaned gene alignments using IQ-TREE with the appropriate substitution model chosen using the ModelFinder and 1000 ultrafast bootstrap replicates (Lanfear et al., 2012; Nguyen et al., 2015; Kalyaanamoorthy et al., 2017; Hoang et al., 2018). Newick Utils v1.6 was used to collapse branches with a BS value of <10, and TreeShrink was used to automatically remove branches with outlying length (Junier and Zdobnov, 2010; Mai and Mirarab, 2018). A species tree was generated from the cleaned gene trees using ASTRAL v5.7.8, and node support was assessed with posterior probability (PP; Mirarab et al., 2014). Nodes with BS <90 and PP <0.9 were considered to have low support, nodes with BS = 90–97 and PP = 0.9–0.97 were considered moderately supported, while nodes with BS = 97–99 and PP = 0.97–0.99 were considered to have high support. Nodes with BS = 100 and PP = 1.0 received maximum support in our analyses.

### Divergence time estimation

To date the Sapindales phylogenetic tree, 29 fossils were selected from the literature as calibrations (**Table 1**). The reliability of each fossil’s identification and age was rigorously assessed and scored following the approach used in a previous angiosperm-wide fossil calibration dataset (Ramírez-Barahona et al., 2020) and using best practices for justifying fossil calibrations (Parham et al., 2012; **Supplementary Material 3**). A conservative approach to calibration was employed, with fossils assigned to the stem node of the taxon or clade the fossil was assigned to. Full justification of node assignment for each fossil calibration is given in **Supplementary Material 3**.

**Table 1:** Summary of fossils used to calibrate the genus-level molecular dating analysis of Sapindales.

	Family	Taxon	Organ/s	Country	Oldest stratum	Min. age (Ma)	Node calibrated	Reference
C02	Sapindaceae	†*Sapindospermum nitidum* Knobloch & Mai	Seed	Czech Republic	Turonian	89.8	crown Sapindales	Knobloch and Mai (1986)
C03	Sapindaceae	†*Aesculus hickeyi* Manchester	Leaf, fruit, seed	USA	Paleocene	56	crown Sapindaceae	Manchester (2001a)
C04	Sapindaceae	†*Dipteronia brownii* McClain & Manchester	Fruit	USA	Middle Paleocene	60	stem *Dipteronia*	McClain and Manchester (2001)
C05	Sapindaceae	†*Acer* sp.	Leaf		Late Paleocene	56	stem *Acer*	Crane (1990)
C06	Sapindaceae	†*Koelreuteria allenii* (Lesq.) W. N. Edwards	Fruit	USA	Eocene	52	stem *Koelreuteria*	Wang et al. (2013)
C07	Sapindaceae	†*Allophylus graciliformis* (Berry) Berry	Leaf	Argentina	Early Eocene	46	stem *Allophylus*	Panti (2020)
C08	Burseraceae	†*Bursera inaequalateralis* (Lesq.) MacGintie	Leaf	USA	Eocene	48.5	crown Burserinae	MacGinitie (1969)
C09	Burseraceae	†*Bursericarpum aldwickens*e Chandler	Pyrene, seed	UK	Ypresian	47.8	stem *Protium* alliance	Chandler (1961)
C10	Anacardiaceae	†*Coahuiloxylon terrazasiae* Estrada-Ruiz, Martínez-Vabrera & Cevallos-Ferriz	Wood	Mexico	Campanian	72.1	stem Anacardiaceae	Estrada-Ruiz et al. (2010)
C11	Anacardiaceae	†*Choerospondias sheppeyensis* (Reid & Chandler) Chandler	Fruit, seed	UK	Ypresian	47.8	crown Anacardiaceae	Reid and Chandler (1933); Chandler (1961)
C12	Anacardiaceae	†*Dracontomelon macdonaldii* (Berry) Herrera, Manchester & Jaramillo	Fruit, seed	Panama	Late Eocene	33.9	crown Spondioideae (-*Campnosperma*)	Herrera et al. (2012)
C13	Anacardiaceae	†*Spondias rothwellii* Herrera, Carvalho, Jaramillo & Manchester	Fruit, seed	Panama	Early Miocene	18.5	stem *Spondias*	Herrera et al. (2019)
C14	Anacardiaceae	†*Anacardium germanicum* Manchester et al.	Fruit	Germany	Middle Eocene (Lutetian assumed)	41.2	stem *Fegimanra* + *Anacardium*	Manchester et al. (2007)
C15	Anacardiaceae	†*Mangifera paleoindica* Sawangchote, Grote, and Dilcher	Leaf	Thailand	Late Oligocene	23.03	stem *Mangifera*	Sawangchote et al. (2009)
C16	Anacardiaceae	†*Cotinus fraterna* (Lesquereux) MacGinitie	Leaf	USA	Late Eocene	33.9	stem *Cotinus*	MacGinitie (1953); Manchester (2001b)
C17	Anacardiaceae	†*Pistacia* sp.	Pollen	Austria	Middle Miocene (Middle to Upper Serravallian)	12.7	stem *Pistacia*	Grímsson et al. (2020)
C18	Anacardiaceae	†*Loxopterygium laplayense* Burnham and Carranco	Fruit	Ecuador	Middle Miocene	13	stem *Loxopterygium*	Burnham and Carranco (2004)
C19	Simaroubaceae	†*Ailanthus confucii* Unger	Fruit	Germany	Middle Eocene (Lutetian assumed)	41.2	stem *Ailanthus + Picrasma*	Collinson et al. (2012)
C20	Meliaceae	†*Cedrela* sp.	Leaf	USA	Eocene	51	crown Cedreloideae	Hickey and Hodges (1975)
C21	Meliaceae	†*Cedrela merrilli* (Chaney) Brown	Leaf, seed	USA	Late Eocene	36.21	stem *Cedrela* + *Toona*	Meyer and Manchester (1997); Manchester and McIntosh (2007)
C22	Meliaceae	†*Swietenia miocenica* Castañeda-Posadas & Cevallos-Ferriz	Flower	Mexico	Late Oligocene	22.5	stem *Swietenia*	Castañeda-Posadas and Cevallos-Ferriz (2007)
C23	Meliaceae	†*Manchestercarpa vancouverensis* Atkinson	Fruit, seed	Canada	Middle Campanian	72.1	crown Meliaceae	Atkinson (2020)
C24	Rutaceae	†*Rutaspermum biornatum* Knobloch & Mai	Seed	Germany	Maastrichtian	66	crown Rutaceae	Knobloch and Mai (1986)
C25	Rutaceae	†*Clausena* sp.	Leaf	Ethiopia	Late Oligocene	27.23	stem *Clausena* + *Glycosmis*	Pan (2010)
C26	Rutaceae	†*Citrus linczangensi*s Xie et al.	Leaf	China	Late Miocene	11.6	stem *Citrus*	Xie et al. (2013)
C27	Rutaceae	†*Ptelea paliuruoides* (Brown) Manchester & O’Leary	Fruit, seed	USA	Middle Eocene	48.5	stem *Ptelea* + *Peltostigma* + *Plethadenia* + *Decazyx* clade	Manchester and O’Leary (2010)
C28	Rutaceae	†*Zanthoxylum* sp.	Seed	UK	Ypresian	47.8	stem *Zanthoxylum*	Chandler (1961)
C29	Rutaceae	†*Euodia costata* (Chandler) Tiffney	Seed	UK	Latest Paleocene (Thanetian)	56	crown Rutoideae clade	Tiffney (1981)
C30	Rutaceae	†*Vepris* sp.	Leaf	Ethiopia	Late Oligocene	27.23	stem *Vepris*	Pan (2010)

Further details of each fossil and justification for their placement are provided in **Supplementary Material 3**.

The dagger symbol (†) indicates fossil taxa.

Computational efficiency of the dating analysis was optimized through gene-shopping, as implemented in SortaDate (Smith et al., 2018). Gene trees were filtered firstly by their similarity to the species tree, secondly by clock-likeness (as indicated by root-to-tip variance), and thirdly by tree length. The three best loci according to these criteria were selected for downstream dating analyses. Three loci were chosen to facilitate time-efficient completion of dating analyses, and because the inclusion of more data is unlikely to improve results, with recent studies suggesting that age calibration priors are the major influence on dating analysis results rather than the quantity of sequence data included (Dos Reis and Yang, 2013; Foster et al., 2017; Sauquet et al., 2022). These loci were aligned using MAFFT with the -auto flag to automatically select alignment strategy (Katoh and Standley, 2013).

Bayesian divergence time estimations were carried out in BEAST v2.6.6 after setting parameters in BEAUti (Bouckaert et al., 2019). Although outgroup topology was not consistent with APG, all outgroup representatives fall within the Pentapetalae, and so the crown age of Pentapetalae was used as the root calibration for the dating analyses. However, the crown age of the angiosperms is uncertain, with molecular and fossil-based studies supporting both a young (Lower Cretaceous) and old angiosperm crown age (Lower Jurassic), resulting in both relatively young and old ages for crown Pentapetalae (Ramírez-Barahona et al., 2020; Silvestro et al. 2021; Sauquet et al., 2022). Given the strong influence of root calibrations on the age of Sapindales families (Muellner-Riehl et al., 2016), the choice of an old or young secondary calibration for the Pentapetalae root prior is likely to affect the results of the current dating analysis. For this reason, three dating analyses were conducted with three alternative ages for the age of Pentapetalae, as estimated by Ramírez-Barahona et al. (2020). Based on dating analyses of the angiosperms with many carefully selected fossil calibrations and three alternative root calibrations, Ramírez-Barahona et al. (2020) reported three possible age ranges for the Pentapetalae, with the crown of Pentapetalae being dated to be between 140.33–144.29 Ma in the ‘CC-complete’ analysis, 143.91–147.94 Ma in the ‘RC-complete’ analysis, and 212.25–221.02 Ma in the ‘UC-complete’ analysis. The 95% HPDs for the age of crown Pentapetalae for the CC-complete, RC-complete and UC-complete analyses of Ramírez-Barahona et al. (2020) were therefore applied as the bounds of a uniform prior in three separate BEAST analyses, with the CC-complete incorporating ages of Pentapetalae taken from a young-angiosperm scenario, RC-complete analysis representing an scenario where angiosperms are assumed to be older, and the UC-complete analysis incorporates ages for Pentapetalae from an analysis where angiosperms were assumed to be very old. These three analyses were run with a fixed tree topology, a Birth-Death tree prior, an uncorrelated log-normal (relaxed) clock model, and with all primary fossil calibrations as uniform priors, with the maximum age boundary set to the maximum age of crown Pentapetalae. To fix the tree topology and maximise computational efficiency, the starting tree was assigned to the best maximum likelihood concatenated tree and topology exchange operators were disabled (i.e., Wide Exchange, Nanon Exchange, Wilson Balding and Subtree-slide; Bouckaert et al., 2019). Ten runs of each model were conducted, each with a chain length of 50,000,000 and with trees sampled every 1,000 generations, resulting in a combined tree exploration space where most priors and statistics reached an effective sampling size (ESS) >200 and all priors and statistics had an ESS >100. Runs were checked for convergence and stationarity in Tracer v1.7.2, and every 50,000th tree was sampled from each run after a burn-in of 20% and combined using logCombiner, and TreeAnnotator was used to generate the consensus tree (Rambaut et al., 2018; Bouckaert et al., 2019).

To test the effect of the tree prior and distribution of the fossil priors, two additional sensitivity analyses were conducted using the RC-complete root calibration. The tree prior sensitivity analysis was performed as described above but with a Yule tree prior (instead of a Birth-Death tree prior). The fossil prior distribution sensitivity analysis was conducted as described above but with log-normal distributions on the fossil calibrations (instead of uniform distributions), with the minimum age of the fossil set to the offset age of the distribution, the mean rounded up to the nearest 5 Ma, and a sigma value of 1.0.

## Results

### Locus recovery and paralogy

The final Sapindales dataset comprised 472 species (including 24 outgroup representatives) with an average of 324 loci recovered per sample and 74% target coverage per locus (**Supplementary Material 2**). On average, 15 (0–44) loci with an outlying proportion of SNPs were removed per sample in the cleaning steps of HybPhaser.

**Figure 1:**
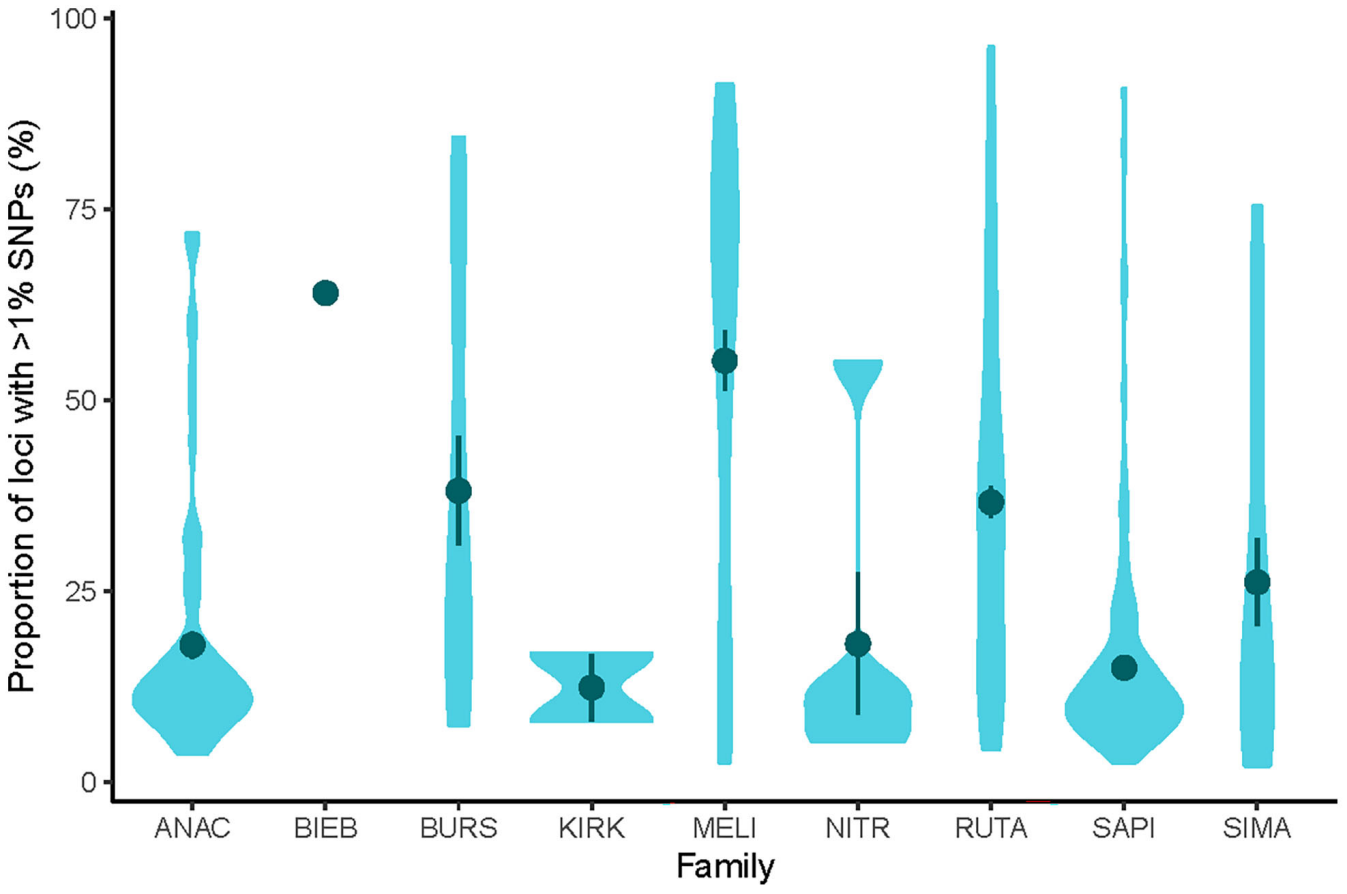
Violin plots of mean proportion of paralogous loci (loci with >1% SNPs) of each family in Sapindales as calculated with HybPhaser. ANAC, Anacardiaceae (n = 79), BIEB, Biebersteiniaceae (n = 1), BURS, Burseraceae (n = 16), KIRK, Kirkiaceae (n = 2), MELI, Meliaceae (n = 56), NITR, Nitrariaceae (n = 5), RUTA, Rutaceae (n = 136), SAPI, Sapindaceae (n = 134), SIMA, Simaroubaceae (n = 18).

In the cleaned Sapindales dataset, 63% (15–99) of loci contained one or more SNP, and the mean allele divergence was 1.33% (0.89–7.52). While the presence of a low number of SNPs can be expected in any orthologous locus, a high proportion of SNPs in a locus (e.g. >1% SNPs per locus) in the cleaned dataset is more likely to be indicative of multiple copies of that locus in the data (i.e., paralogy). Therefore, to differentiate allelic variation from paralogy, we consider loci with >1% SNPs to be paralogous (i.e., have multiple gene copies). Overall, 28.55% (2.14–96.31) of Angiosperms353 loci for Sapindales contained >1% SNPs (i.e., were paralogous). Variation in the degree of paralogy and allele divergence was unevenly spread across the order. Meliaceae showed substantially higher levels of paralogy and allele divergence relative to other Sapindales families, with an average of 51% ± 4.11 of loci with >1% SNPs and an average allele divergence of 2.97 ± 0.27 (**Figures 1**, **2**). Kirkiaceae had the lowest level of paralogy and allele divergence, with 11% ± 3.89 of loci with >1% SNPs and a mean allele divergence of 0.40 ± 0.086 (**Figures 1**, **2**). Similar patterns in paralogy and allele divergence were observed when the threshold for paralogy was raised to >2% SNPs (shown in **Supplementary Material 4**).

### Phylogenetic relationships

The concatenated alignment was 194,132 bp long and comprised 330 loci with 135,613 parsimony-informative sites and 14.73% gaps or ambiguities. IQ-TREE identified the best partition scheme and merged the alignment into 45 partitions (lnL = -10642504, df =1822), all of which were allocated an optimal substitution model with the ModelFinder function of IQ-TREE. After 173 tree search iterations and 1000 bootstrap trees, IQ-TREE produced a consensus tree with a log-likelihood of -10642396 (**Figure 2**).

**Figure 2A:**
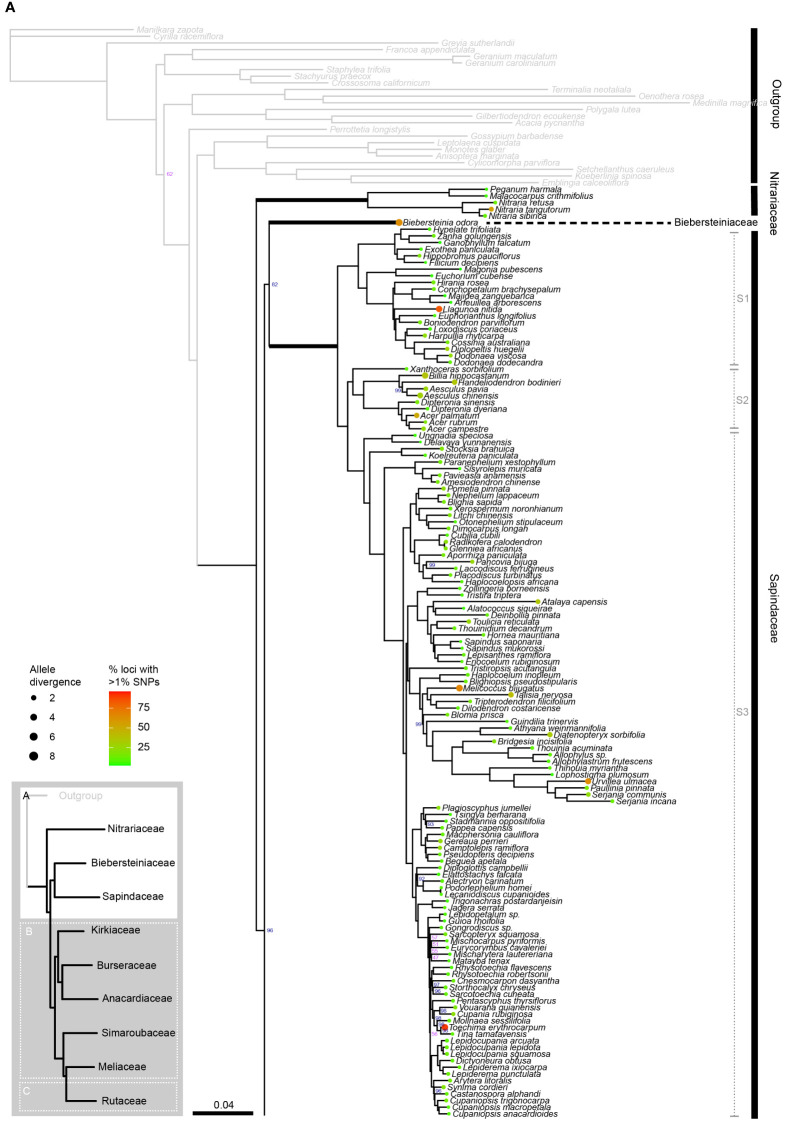


**Figure 2B:**
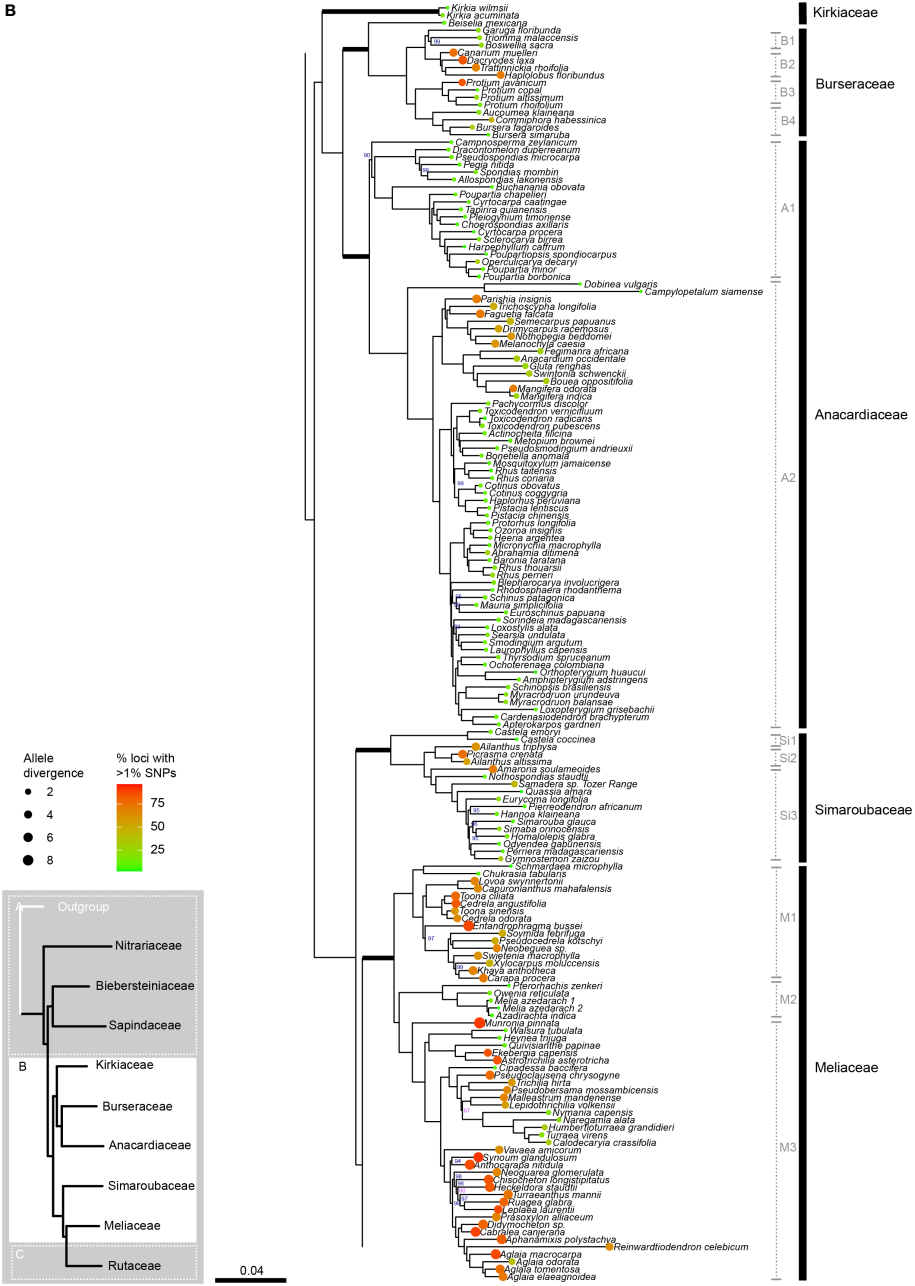


**Figure 2C:**
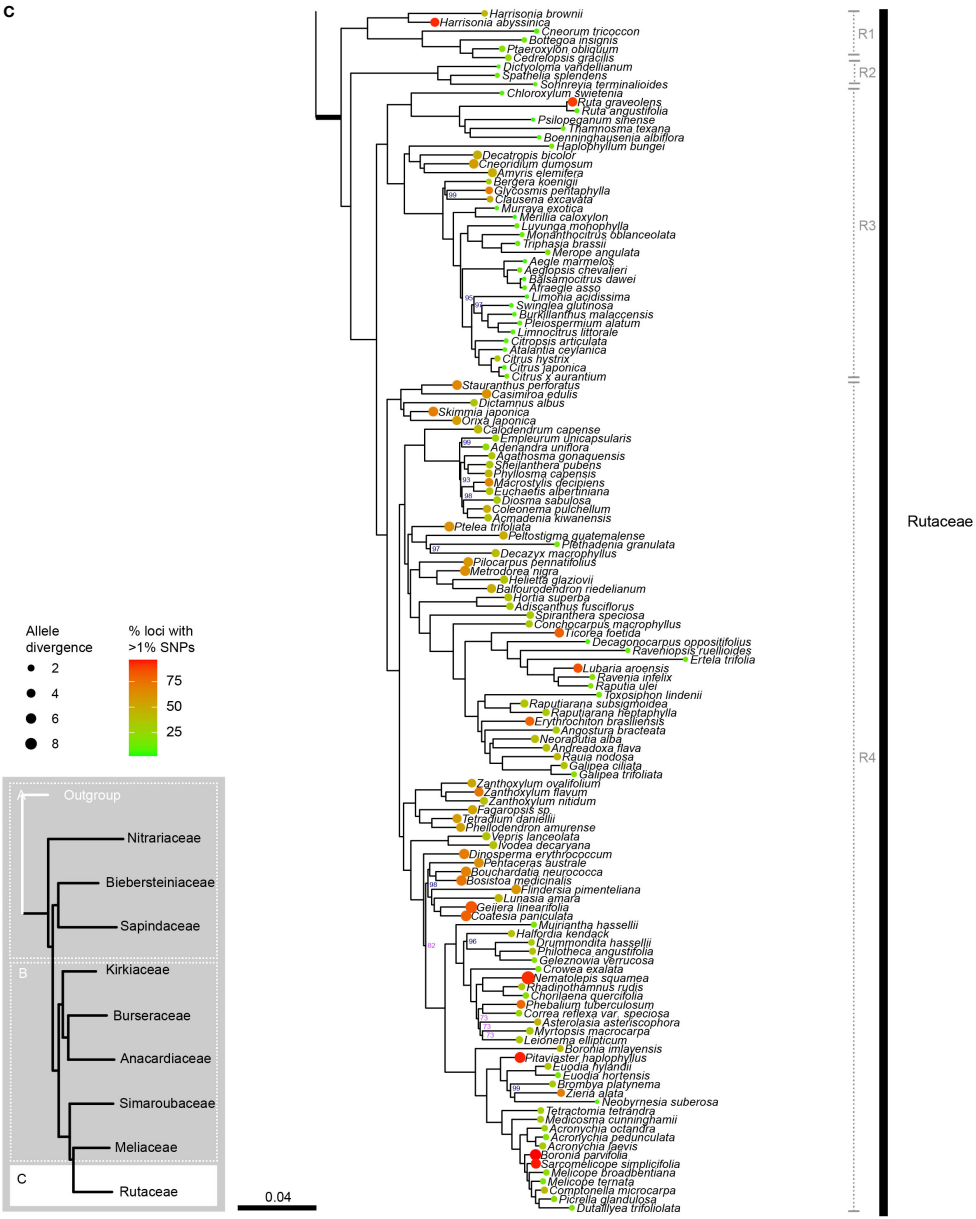
Phylogenetic relationships in Sapindales based on concatenated analyses of 324 nuclear loci. **(A)**, Nitrariaceae, Biebersteiniaceae, Sapindaceae; **(B)**, Kirkiaceae, Burseraceae, Anacardiaceae, Simaroubaceae, Meliaceae, and **(C)**, Rutaceae. Numbers on branches indicate support for nodes with low support (BS<90%; violet) or moderate to high support (BS>90%; blue); branches without bootstrap values have maximum support (BS = 100%). Thick branches indicate stems of families, with major family clades annotated in grey to the right. Tip circle colour indicates the percentage of paralogous loci (= percentage of loci with >1% SNPs), and tip circle size is proportional to allele divergence for the accession.

**Figure 3:**
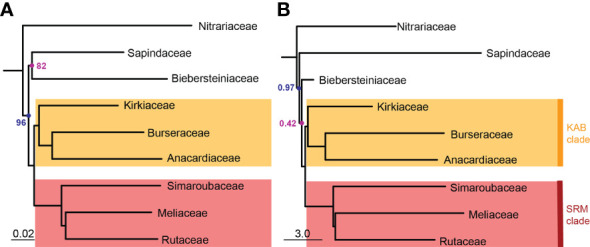
Pruned **(A)** concatenated with bootstrap values and **(B)** multispecies coalescent tree with posterior probabilities showing retrieved family topology in Sapindales. Nodes without annotation were retrieved with maximum support (BS = 100 or PP = 1.0). For the complete concatenated tree refer to **Figure 2**; for the complete coalescent tree refer to **Supplementary Material 5**. The scale bar in **(A)** denotes the expected number of substitutions per site; in **(B)** it corresponds to coalescent units for internal branches (not terminal branches). Note that Biebersteiniaceae only comprised one sample, resulting in the short branch length on the multispecies coalescent tree.

Sapindales and all Sapindales families were found to be monophyletic with maximum support in both the concatenated and multispecies coalescent analyses. Nitrariaceae was retrieved as sister to the rest of Sapindales with moderate support (BS = 96, PP = 0.97) in both analyses (**Figure 3**). In the concatenated analysis, Sapindaceae and Biebersteiniaceae were retrieved as sister families, whereas in the coalescent analysis they were placed as a grade; however, in both cases, the relationships of Sapindaceae and Biebersteiniaceae received poor support (**Figure 3**). The remaining families grouped into two clades that were consistent and well-supported in both analyses: the ‘KAB clade’ with Kirkiaceae sister to Anacardiaceae + Burseraceae, and the ‘SRM clade’ with Simaroubaceae sister to Rutaceae + Meliaceae.

Within Sapindaceae, three major clades were retrieved in both the concatenated and multispecies coalescent analyses (**Figure 2A**, clades S1, S2, and S3; **Supplementary Material 5**). These clades had maximum support, as did most generic relationships within the family. *Xanthoceras* was consistently retrieved as sister to clade S2 with maximum support in both the multispecies coalescent and concatenated analyses (**Figure 2A**; **Supplementary Material 5**). The greatest uncertainty in generic relationships was found in clade S3, particularly among *Eurycorymbus, Matayba, Mischarytera, Mischocarpus*, and *Sarcopteryx*, (BS = 47–57; **Figure 2A**). The ancestral node of the clade containing *Cupaniopsis* and the clade containing *Rhysotoechia* were also poorly supported (BS = 56; **Figure 2A**).

In Burseraceae, *Beiselia* was found to be sister to the rest of the family with maximum support in both the concatenated and coalescent analyses (**Figure 2B**; **Supplementary Material 5**). The remaining Burseraceae genera were retrieved in four well-supported main clades (**Figure 2B**, clades B1–4). The relationships between all Burseraceae genera were well-supported in both the concatenated and coalescent analyses, with the exception of the sister relationship of *Dacryodes* and *Canarium* retrieved in the coalescent analysis (**Supplementary Material 5**).

Within Anacardiaceae, two major clades were recovered (**Figure 2B**, clades A1 & A2). In the concatenated analysis, *Campnosperma* was retrieved as a crown member of clade A1 with moderate support (BS = 90), but in the coalescent analysis it was placed as sister to clades A1 and A2 with low support (**Supplementary Material 5**, PP = 0.69). *Dobinea* and *Campylopetalum* formed a well-supported clade sister to clade A2 in both analyses. *Cyrtocarpa* was not resolved as monophyletic since the two sampled species (*C. procera* and *C. caatingae*) were placed in different subclades within clade A1. Similarly, the genus *Rhus* was not monophyletic, with *R. taitensis* + *R. coriaria* placed separately from *R. thouarsii* + *R. perrieri* within clade A2. Other genera for which multiple accessions were included (*Cotinus*, *Mangifera*, *Pistacia*, and *Toxicodendron*) were retrieved as monophyletic.

Three major clades were recovered in Simaroubaceae with maximum support in the concatenated and coalescent analyses (**Figure 2B**, clades Si1–3; **Supplementary Material 5**). Clade Si1 comprises the monophyletic genus *Castela* that was placed as sister to the rest of the family. Clade Si2 contains *Ailanthus* and *Picrasma*, with *Ailanthus* retrieved as paraphyletic in relation to *Picrasma crenata* in both the concatenated and coalescent analyses (**Figure 2B**; **Supplementary Material 5**). The third clade, Si3, contains the remaining 13 sampled genera, predominantly arranged in a grade with high to maximum support for nodes in the concatenated analysis, and low to maximum support in the coalescent tree (**Figure 2B**; **Supplementary Material 5**).

In Meliaceae, three major clades were recovered with high support in both the concatenated and coalescent analyses (**Figure 2B**, clades M1–3; **Supplementary Material 5**). Within clade M1, *Chukrasia* and *Schmardaea* form a clade sister to the rest of the genera. *Cedrela* and *Toona* form a clade together, and are mutually paraphyletic. In clade M2, *Pterorhachis* and *Owenia* are successive sisters leading to a clade containing *Azadirachta* and *Melia*. *Melia* was paraphyletic in relation to *Azadirachta* in both analyses. In clade M3, *Munronia* was placed as sister to the rest of the clade. The relationship of the subclade containing *Calodecaryia*, *Humbertioturraea*, *Naregamia*, *Nymania*, and *Turraea* was uncertain, being placed as sister to a subclade of *Lepidotrichilia* + *Malleastrum* in the concatenated analysis with low support (BS = 87), and sister to the subclade of *Pseudobersama* + *Trichilia* in the coalescent analysis with no support (PP = 0.38). The placement of *Vavaea* was consistent across analyses with strong support, but the relationships of the subclade comprising *Anthocarapa*, *Chisocheton*, *Heckeldora*, *Leplaea*, *Neoguarea*, *Ruagea, Synoum*, and *Turraeanthus* received moderate to weak support (**Figure 2B**; **Supplementary Material 5**). Additionally, the placement of *Reinwardtiodendron* differed slightly between coalescent and concatenated analyses, with *Aphanamixis* sister to a clade containing *Aglaia* + *Reinwardtiodendron* in the concatenated analysis, but *Reinwardtiodendron* retrieved as sister to a clade containing *Aglaia* + *Aphanamixis* in the coalescent analysis (**Figure 2B**; **Supplementary Material 5**). *Aglaia* was retrieved as monophyletic with maximum support in both trees.

Rutaceae comprised four main clades, all receiving maximum support in both the concatenated and coalescent analyses (**Figure 2C**, clades R1–4; **Supplementary Material 5**). Clades R1 and R2 form a grade (with five and three genera respectively), and the larger clades R3 and R4 were retrieved as sisters. Generic relationships had high to maximum support in clades R1, R2 and R3, with two main exceptions in clade R3: the placement of the clade containing *Aegle, Aeglopsis, Afraegle*, and *Balsamocitrus* received moderate to low support (BS = 95, PP = 0.51), as did the placement of the clade containing *Burkillanthus*, *Pleiospermium*, *Limnocitrus*, and *Swinglea* (BS = 97, PP = 0.44) (**Figure 2C**; **Supplementary Material 5**). Relationships in clade R4 were generally less well supported, with inconsistencies in the topologies of the concatenated and coalescent trees. Major topological differences were in the placement of *Flindersia, Nematolepis*, and *Ptelea*, and in the placement of the clade containing *Decazyx*, *Peltostigma*, and *Plethadenia* (**Figure 2C**; **Supplementary Material 5**). All genera were monophyletic in both trees, with the exception of *Boronia* and *Melicope* in both the concatenated and coalescent analyses (**Figure 2C**; **Supplementary Material 5**).

### Divergence time estimation

The three optimal loci selected for dating analyses were loci 5162, 5333 and 6091, resulting in an alignment of 4,738 bp. The ages for major clades in Sapindales were similar across chronograms regardless of whether they were estimated with the CC-complete or RC-complete root priors, with Cretaceous stem and crown ages for the order and all families. However, in the UC-complete analysis (which constrained the analysis to ages from a scenario assuming the angiosperms to be very old) major Sapindales clades were considerably older. In this analysis, the stem and crown nodes of Sapindales were in the Jurassic, as were stem nodes of Nitrariaceae, Biebersteiniaceae, Sapindaceae, Simaroubaceae, and Kirkiaceae. The crown nodes of Sapindaceae and Simaroubaceae were in the Lower Cretaceous. Meliaceae, Burseraceae, Anacardiaceae, and Rutaceae had Lower Cretaceous stem nodes, and crown nodes at the Lower-Upper Cretaceous boundary (**Supplementary Materials 6**–**9**). Given the similarity of the CC-complete and RC-complete analyses, we herein focus on the results of the RC-complete and UC-complete analyses, to compare the results of when a younger-Pentapetalae and older-Pentapetalae scenario, respectively, is adopted.

In the analysis using the RC-complete root constraint, the age of the stem node of Sapindales was estimated at c. 131 (124–137) Ma (**Figure 4A**). Nitrariaceae, Biebersteiniaceae, and Sapindaceae diverged in the Lower Cretaceous with an estimated stem age of 124 (117–130) Ma for Nitrariaceae (making this the crown age of Sapindales), and 122 (114–128) Ma for Biebersteiniaceae and Sapindaceae (**Supplementary Material 6**; **Figure 4A**). The major split from the most recent common ancestor of the KAB and SRM clades was estimated to have occurred in the Lower Cretaceous, c. 118 (110–125) Ma. Kirkiaceae emerged approximately 109 (100–119) Ma, and Burseraceae and Anacardiaceae split from their most recent common ancestor at approximately 100 (89–111) Ma (**Figure 4B**). Simaroubaceae diverged at c. 108 (100–117) Ma, and Meliaceae and Rutaceae diverged from their most recent common ancestor at c. 104 (96–114) Ma (**Figures 4B, C**). The estimated crown ages for Nitrariaceae and Sapindaceae were c. 72 (48–102) and 103 (94–113) Ma, respectively. The estimated crown ages for Kirkiaceae, Anacardiaceae, and Burseraceae are 2 (0.2–5), 88 (77–100), and 85 (71–99) Ma, and the crown ages for Simaroubaceae, Rutaceae, and Meliaceae are 83 (66–102), 97 (87–107), and 86 (72–98) Ma, respectively.

**Figure 4A:**
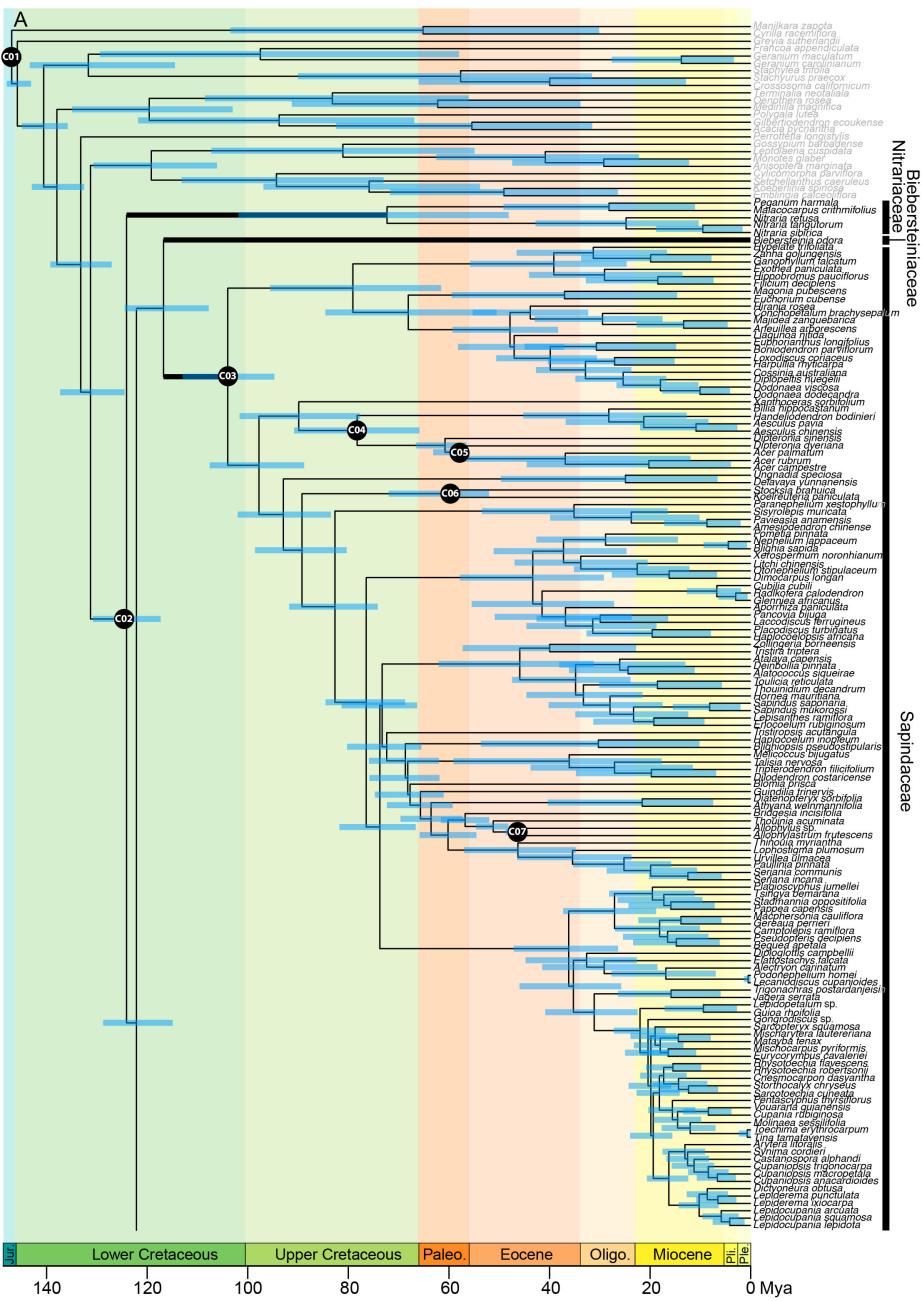


**Figure 4B:**
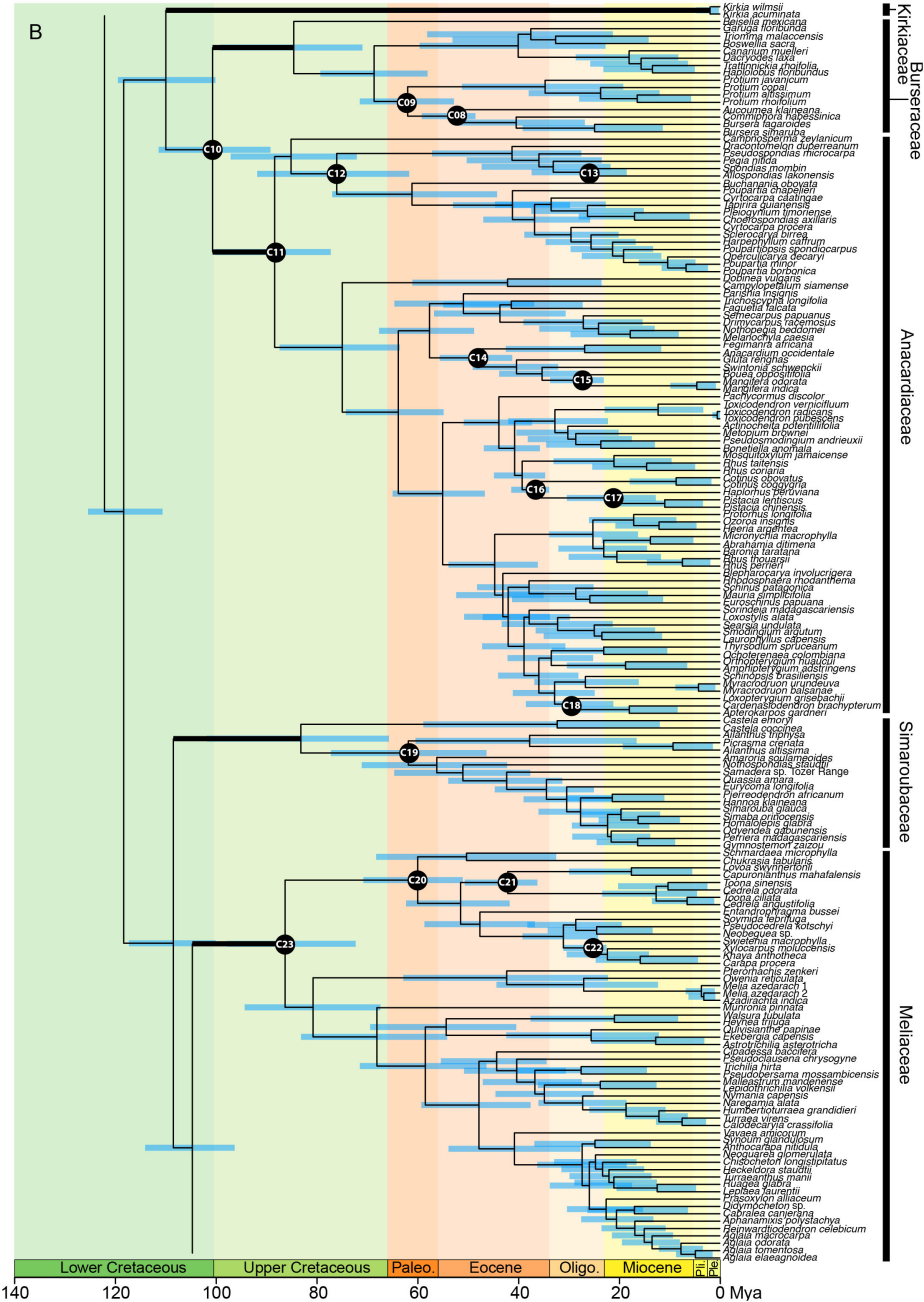


**Figure 4C:**
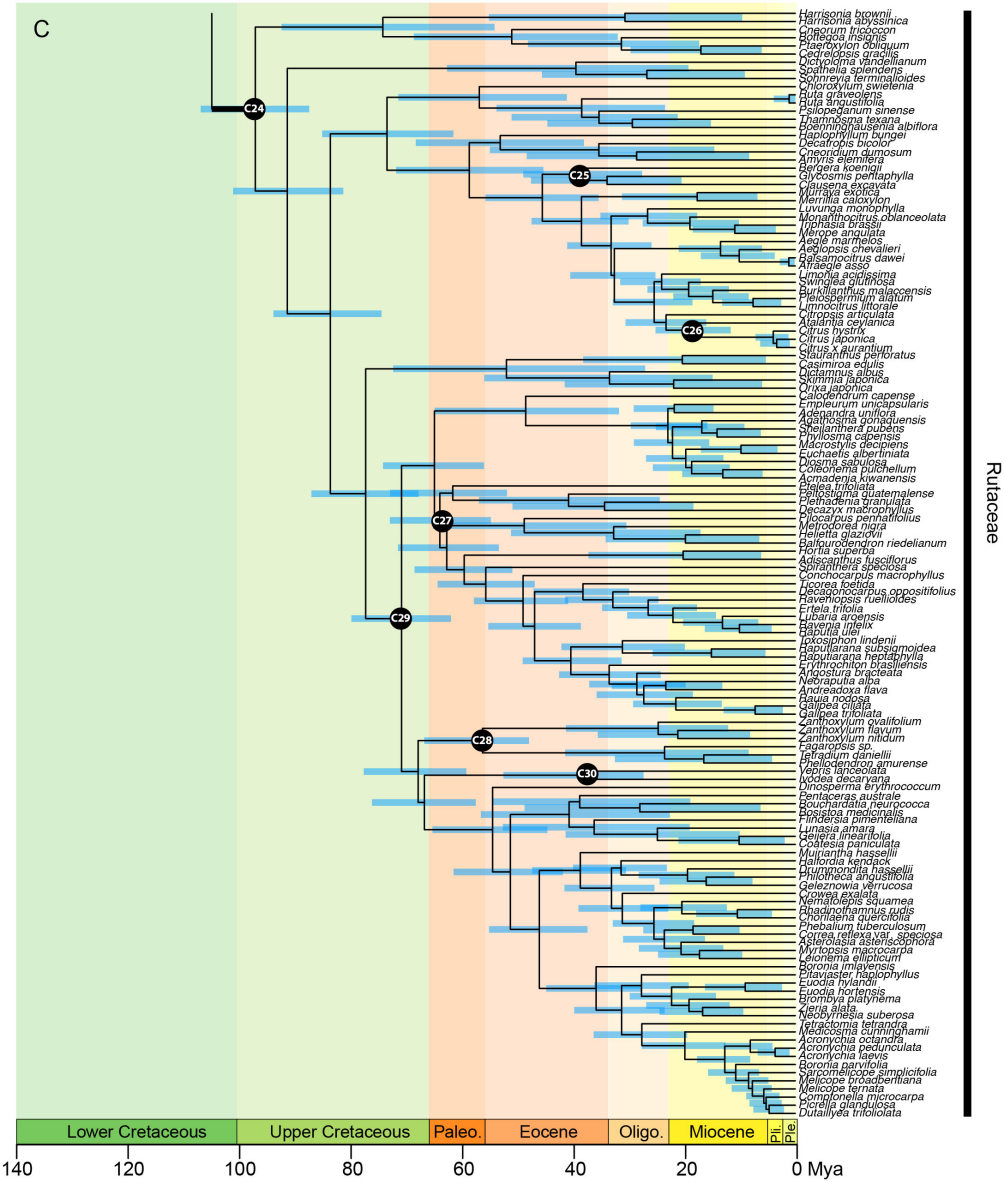
Chronogram of Sapindales as estimated under a relaxed-clock model with a normal secondary root prior distribution (node C01) and uniform primary calibrations. Tree topology was fixed to the concatenated tree (see **Figure 2**). **(A)** shows Nitrariaceae, Biebersteiniaceae, Sapindaceae; **(B)** shows Kirkiaceae, Burseraceae, Anacardiaceae, Simaroubaceae, Meliaceae, and **(C)** shows Rutaceae. Black circles with C01-C30 signify calibrated nodes (see **Table 1** for fossil information); blue bars represent 95% highest posterior densities (HPDs); thickened branches indicate stem branches of families.

In the analysis using the UC-complete root constraint (where an ‘old-Pentapetalae’ scenario is assumed), the age of the stem node of Sapindales was estimated at c. 186 (174–199) Ma (**Supplementary Materials 6**, **9**). Nitrariaceae, Biebersteiniaceae, and Sapindaceae diverged in the Middle Jurassic with an estimated stem age of 172 (159–187) Ma for Nitrariaceae (making this the crown age of Sapindales), and 169 (153–182) Ma for Biebersteiniaceae and Sapindaceae (**Supplementary Materials 6**, **9**). The major split from the most recent common ancestor of the KAB and SRM clades was estimated to have occurred at the Upper/Middle Jurassic boundary, c. 162 (146–176) Ma. Kirkiaceae emerged approximately 148 (131–165) Ma, and Burseraceae and Anacardiaceae split from their most recent common ancestor at approximately 134 (116–151) Ma (**Supplementary Materials 6**, **9**). Simaroubaceae diverged at c. 145 (128–161) Ma, and Meliaceae and Rutaceae diverged from their most recent common ancestor at c. 139 (123–157) Ma (**Supplementary Materials 6**, **9**). The estimated crown ages for Nitrariaceae and Sapindaceae were c. 103 (63–145) and 135 (117–151) Ma, respectively. The estimated crown ages for Kirkiaceae, Anacardiaceae, and Burseraceae are 2 (0.28–5), 114 (96–131), and 108 (84–130) Ma, and the crown ages for Simaroubaceae, Rutaceae, and Meliaceae are 109 (85–134), 128 (111–145), and 109 (89–132) Ma, respectively.

Using a Birth-Death tree prior made little difference to the ages of the chronogram, with the major clades of Sapindales an average of only 0.13 Ma younger when estimated with a Birth–Death tree prior as opposed to a Yule prior (**Supplementary Materials 10**, **11**). Likewise, the application of log-normal prior distributions for fossil calibrations made little difference to the ages of the major nodes of Sapindales, with major nodes only 1–2 Ma younger than the same analysis with uniformly distributed fossil priors, and with overlapping HPD intervals.

## Discussion

### The rise of Sapindales families in the Mid-Cretaceous Hothouse

Our results strongly support the monophyly of Sapindales, with the stem lineage of the order evolving at c. 131 (124–137) Ma or c. 186 (174–199) Ma, depending on whether angiosperms (and Pentapetalae) are assumed to be younger, or older, respectively. The former estimate is slightly older — and the latter much older — than ages estimated in previous studies, such as the angiosperm-wide study of Magallón et al. (2015) and the Sapindales chronogram of Muellner-Riehl et al. (2016), who dated the stem node of Sapindales to be 104 (98–112) and 111 (106–117) Ma, respectively. Our dating analyses place the emergence of the order c. 41 or 96 Ma before the preservation of the oldest-known fossil of Sapindales (a seed of †*Sapindospermum nitidum* from the Czech Republic; Knobloch and Mai, 1986). In both scenarios, the short stem of the order followed by the rapid succession of family divergences suggests extensive diversification in the Lower-Mid Cretaceous, coincident with a global warming period and the persistence of ancient Sapindales lineages since then (Scotese, 2021). Regardless of the crown age of the angiosperms, the Mid-Cretaceous Hothouse is modelled to have had a substantial effect on the evolution of Sapindales families. When angiosperms are assumed to be relatively young (and thus the age of crown Pentapetalae is modelled to be younger), most families diverged and diversified during the Mid-Cretaceous Hothouse period. If crown angiosperms are assumed to be ancient (and thus the age of crown Pentapetalae is older), Sapindales families emerged just prior to the Mid-Cretaceous Hothouse period and diversification of the families occurred during the Mid-Cretaceous Hothouse. Although both scenarios are possible, there is a chance that ages estimated with the ancient angiosperm scenario are overestimated; in this scenario, the maximum age of crown angiosperms is considered to be coincident with the first appearance of angiosperm-like pollen grains in the fossil record (247 Ma). However, it is possible that these angiosperm-like pollen grain fossils are stem angiosperm relatives rather than crown angiosperm members, and so when applied as a maximum age for the crown of the angiosperms, this calibration could lead to an overestimation of crown-group ages, including the estimated age of the Pentapetalae used in our analysis. This risk is likely compounded by the use of uniform priors in the analysis, whereby older ages close to the appearance of angiosperm-like pollen grains in the fossil record would be just as likely as younger ages.

Novel to this study, our analyses suggest that the well-supported KAB and SRM clades are sisters, and that Sapindaceae does not fall within these clades. This contrasts with previous studies that have tentatively placed Sapindaceae within the KAB clade, or as sister to the SRM clade (Chase et al., 1993; Gadek et al., 1996; Appelhans et al., 2012; Muellner-Riehl et al., 2016; Lin et al., 2018; Li et al., 2019; Ramírez-Barahona et al., 2020; Li et al., 2021). However, despite dense sampling and the inclusion of 330 loci, the relationship between Sapindaceae and Biebersteiniaceae could not be resolved, with these families uncertainly placed as sisters in our concatenated tree and as poorly-supported successive sisters to the KAB + SRM clade in our coalescent tree. As a result, the sister family to the KAB and SRM clades cannot be identified. In line with the results of Li et al. (2021), we consistently retrieved Nitrariaceae as sister to all remaining Sapindales with moderate support. However, the poor support for the nodes leading to Sapindaceae and Biebersteiniaceae means that the relationships between Nitrariaceae, Biebersteiniaceae, and Sapindaceae are perhaps best represented as a polytomy. What is clear from all dating analyses, however, is that these families emerged early and rapidly in the evolution of the order, having diverged within an estimated period of approximately 5 Ma. The difficulty of reconstructing relationships between Nitrariaceae, Biebersteiniaceae, and Sapindaceae may be due to this rapid and ancient divergence of families, in combination with unavoidable sampling heterogeneity caused by a low within-family diversity in Biebersteiniaceae and Nitrariaceae. As previously suggested by Muellner-Riehl et al. (2016), the low extant diversity of Biebersteiniaceae and Nitrariaceae on long stem branches is likely to be indicative of a prevalent history of extinction relative to other Sapindales families (although it could also be due to low speciation rates, or a combination thereof), and such ‘depauperon’ lineages are often difficult to place in phylogenetic analyses (Donoghue and Sanderson, 2015). Further research with custom loci or whole genomes may be required to resolve the relationships of these families, if it is possible at all. Given the modern-day xeric habitat of Nitrariaceae, if this family is confirmed to be sister to the rest of Sapindales, it could point to an extra-tropical origin for this predominantly tropical order. This hypothesis is supported by a concentration of extant mesic- and xeric-adapted lineages in the Nitrariaceae, Biebersteiniaceae, and Sapindaceae, and the presence of large evaporite and arid belts from the Jurassic-Lower Cretaceous boundary when these families are likely to have evolved (Hay, 2017; Zhang et al., 2018). However, the spatio-temporal diversification dynamics of depauperon lineages such as Nitrariaceae are notoriously complex and difficult to reconstruct (Donoghue and Sanderson, 2015), so this hypothesis needs careful consideration and should be explicitly tested.

Kirkiaceae, Anacardiaceae, and Burseraceae were retrieved as a clade in this study (‘the KAB clade’), adding to a substantial body of biochemical, morphological and molecular evidence suggesting that these families are closely related (Chase et al., 1993; Gadek et al., 1996; Muellner-Riehl et al., 2016). When angiosperms are assumed to be relatively young, the KAB clade was reconstructed as diverging from its sister SRM clade in the Lower Cretaceous, with Kirkiaceae, Anacardiaceae, and Burseraceae diverging at the Lower-Upper Cretaceous boundary. This is coincident with the beginning of the Mid-Cretaceous Hothouse, a period characterised by low-lying land masses, rising sea-levels, and high CO_2_ concentrations and global temperatures (Barral et al., 2017; Scotese, 2021). Kirkiaceae was found to have diverged from the ancestor of Burseraceae and Anacardiaceae at c. 110 (100–119) Ma, and Burseraceae and Anacardiaceae as diverging at c. 101 (89–111) Ma. These results are slightly older than the analyses of Muellner-Riehl et al. (2016) (who estimated the stem age of Kirkiaceae and the Burseraceae/Anacardiaceae split to be 95 [86–103] and 87 [78–97] Ma, respectively), but in line with Weeks et al. (2014), who estimated the divergence of Anacardiaceae and Burseraceae to be at 116 (105–127) Ma. The crown nodes of Anacardiaceae and Burseraceae at 88 (77–100) and 85 (71–99) Ma, respectively, are just after the Cenomanian–Turonian Thermal Maximum of the Mid-Cretaceous Hothouse period, which is thought to mark the time with the highest temperatures and sea levels in the past 250 million years, suggesting it may have been a key period for the diversification of these families (Scotese, 2021). In an ancient-angiosperm scenario, the KAB and SRM clades are reconstructed as diverging in the Jurassic, with Kirkiaceae, Anacardiaceae, and Burseraceae emerging at the Jurassic-Cretaceous boundary, and crown diversification of Anacardiaceae and Burseraceae coincident with the start of the Mid-Cretaceous Hothouse period (rather than the peak). The young crown node of Kirkiaceae in all analyses could be indicative of extensive extinction since the divergence of the family, or of delayed speciation until the Pleistocene. The contrasting temporal patterns of diversification of Kirkiaceae compared to Anacardiaceae and Burseraceae could provide interesting insights into the evolution of depauperon lineages in future studies (Donoghue and Sanderson, 2015).

Simaroubaceae, Rutaceae, and Meliaceae are retrieved as a clade within Sapindales (the ‘SRM clade’), in line with previous studies on the order (Gadek et al., 1996; Muellner et al., 2007; Appelhans et al., 2012; Muellner-Riehl et al., 2016; Lin et al., 2018; Li et al., 2019; Ramírez-Barahona et al., 2020; Li et al., 2021). The SRM clade is united by the presence of nortriterpenoids and the ability to form wood traumatic ducts (Gadek et al., 1996; Kubitzki, 2011; Chuang et al., 2022; Pace et al., 2022). However, in contrast to previous phylogenetic studies suggesting that Simaroubaceae and Meliaceae are sister families (Muellner et al., 2007; Appelhans et al., 2012; Muellner-Riehl et al., 2016), or that Simaroubaceae and Rutaceae are sister families (Gadek et al., 1996; Lin et al., 2018; Li et al., 2019; Ramírez-Barahona et al., 2020; Li et al., 2021), our analysis unequivocally inferred Meliaceae and Rutaceae as sister families. Regardless of what the crown age of the angiosperms was assumed to be, our dating analyses indicate that the three families diverged in an extremely short time, with Simaroubaceae splitting from the ancestor of Rutaceae and Meliaceae in the Lower Cretaceous, and Rutaceae and Meliaceae diverging from their common ancestor within the next 3-6 million years. As with the families in the KAB clade, the diversification of families in the SRM clade then followed to coincide with the peak or end of the Mid-Cretaceous Hothouse (in the UC-complete and RC-complete analysis, respectively) (Scotese, 2021). The rapid divergence of Simaroubaceae, Rutaceae, and Meliaceae could explain the difficulty of reconstructing familial relationships with the smaller datasets of previous studies. Ancient hybridization in the early history of these families could also explain contrasting family topologies in previous studies based on plastid data, and may be supported by the high degree of paralogy in Rutaceae and Meliaceae detected in this study relative to other Sapindales families. Given that our study includes an order of magnitude more data and denser sampling than previous phylogenetic studies of the order, we suggest that it is the most reliable representation of family relationships published to date. However, future research should be conducted with short- and long-read sequences to investigate whether ancient hybridization could be driving contrasting topologies between nuclear and plastid phylogenetic trees.

Morphological and anatomical data should be reconsidered in light of this new phylogenetic framework. Phytochemically, Meliaceae and Rutaceae are characterised by the presence of limonoid nortriterpenoids, while Simaroubaceae is characterised by quassinoid nortriterpenoids (Gadek et al., 1996; Fernandes da Silva et al., 2022). Recently, Chuang et al. (2022) found that biosynthesis of both quassinoids and limonoids have protolimonoid melianol as an intermediate compound, and that this pathway is controlled by conserved genes. Our new phylogenetic hypothesis for the SRM clade raises the possibility that the downstream change in metabolic pathway from protolimonoid melianol to produce limonoids could be a synapomorphy for sister families Meliaceae + Rutaceae. Further research is needed to test the synapomorphies for these groups and explore the potential of other putative phytochemical synapomorphies.

### Divergence of the major Sapindales infra-familial clades

Within Sapindaceae, the three major clades retrieved are congruent with the recent infra-familial classification of Buerki et al. (2021), with S1 corresponding to the subfamily Dodonaeoideae, S2 including monotypic Xanthoceratoideae and monophyletic Hippocastanoideae, and S3 equivalent to Sapindoideae (**Figure 2A**). Our dating analysis suggests that these subfamilies are ancient, with the divergence of Dodonaeoideae, Xanthoceratoideae, Hippocastanoideae, and Sapindoideae aligning with the Cenomanian–Turonian Thermal Maximum of the Upper Cretaceous, regardless of the crown age of the angiosperms (Bentham and Hooker, 1862; Hutchinson, 1926; Gadek et al., 1996; Savolainen et al., 2000). The rich data used in this study has enabled us to reconstruct subfamily topology with high support for the first time, particularly in relation to the position of Xanthoceratoideae. This family includes one species (*Xanthoceras sorbifolium*), a deciduous shrub to small tree that inhabits xeric areas of China. *Xanthoceras* was originally included in Dodonaeoideae (Radlkofer, 1931), but due to its distinct morphological features, habitat, and placement as sister to the remainder of Sapindaceae in molecular studies, it was transferred to its own family and eventually subfamily of Sapindaceae (Harrington et al., 2005; Buerki et al., 2010; Buerki et al., 2021). In previous molecular studies with low numbers of loci, Xanthoceratoideae was always retrieved as sister to the remainder of Sapindaceae with weak support (e.g., BS = 70 in Harrington et al., 2005; BS = 56 in Buerki et al., 2010; BS = 76 in Muellner-Riehl et al., 2016). The more recent Angiosperms353 phylogeny of Sapindaceae by Buerki et al. (2021), which focused on infra-familial taxonomy and did not test the topology within families, rooted their analysis on Xanthoceratoideae, based on the tree of Muellner-Riehl et al. (2016), without outgroup samples from other families. When the tree of Buerki et al. (2021) is re-rooted, their topology agrees with ours, strongly indicating that Xanthoceratoideae is in fact nested within Sapindaceae and sister to Hippocastanoideae (clade S2 of **Figure 2A**). This novel finding has interesting implications for our understanding of the biogeographical and morphological evolution of the family. Based largely on previous inferences of Xanthoceratoideae relationships and its modern distribution, the origin of Sapindaceae was thought to be in Eurasia, with expansions into Gondwana during the Late Paleocene (Buerki et al., 2013). The subfamilial relationships presented here for Sapindaceae brings this interpretation of biogeographical history into question, and deserves further attention.

In Burseraceae, the major clades retrieved are largely congruent with the current classification of the family, with clades B1, B2, B3, and B4 corresponding to the *Boswellia*, *Canarium*, *Protium*, and *Bursera* alliances respectively, and *Beiselia* representing the monotypic *Beiselia* alliance (Daly et al., 2011). Most of these alliances are resolved as monophyletic here, the only exception being the *Boswellia* and *Canarium* alliances due to the placement of *Triomma* in the *Boswellia* alliance instead of the *Canarium* alliance (Daly et al., 2011). While the ages estimated in our UC-complete analyses are older than any study previously published, our RC-complete estimates are in agreement with the study of Weeks et al. (2014), where the *Beiselia* alliance (of which *Beiselia* is the sole, monospecific genus) was retrieved as sister to the rest of Burseraceae, having diverged from the ancestor of the remainder of Burseraceae in the Upper Cretaceous (at 85 [71−99] Ma). In contrast to Weeks et al. (2014), however, our RC-complete and CC-complete analyses suggest that the diversification of Burseraceae into the four major alliances was delayed until the Paleogene. More sampling, particularly in the *Protium* alliance and for the genus *Rosselia* (treated by Daly et al., 2011 as an unplaced genus), is needed to further corroborate infra-familial classification and clarify generic relationships.

Anacardiaceae relationships in this study are largely congruent with previous studies of the family, with our analyses supporting the recognition of two subfamilies: Spondioideae (= clade A1, **Figure 2B**), and Anacardioideae without *Campnosperma* (= clade A2, **Figure 2B**) (Pell, 2004; Pell et al., 2011; Weeks et al., 2014). Historically, placement of *Campnosperma* has alternated between Anacardioideae and Spondioideae (Wannan and Quinn, 1990; Wannan and Quinn, 1991; Pell, 2004; Mitchell et al., 2006; Weeks et al., 2014). In our analyses, the position of *Campnosperma* was not fully resolved, being placed with Spondioideae in the concatenated tree, but as sister to Spondioideae + Anacardioideae in the coalescent analysis. *Campnosperma* diverged from its most recent common ancestor either in the Lower Cretaceous (when an ancient-angiosperm scenario is adopted), or the Upper Cretaceous (when angiosperms are considered to be younger), close to the time of the divergence of Spondioideae and Anacardioideae. This long history of independent evolution could explain the difficulty of classifying *Campnosperma* both in both morphological and molecular studies. Additional data may be needed to resolve its subfamilial assignment. As in previous phylogenetic studies of Anacardiaceae, Engler’s (1876) tribal classification (*sensu* Mitchell and Mori, 1987) appears artificial, with all but Dobineae retrieved as polyphyletic, suggesting that taxonomic revision at this level is needed (Pell, 2004; Weeks et al., 2014). Revision of the generic limits of *Rhus, Poupartia*, and *Cyrtocarpa* is indicated on the basis of their non-monophyly in this analysis, as also suggested by previous studies (Pell et al., 2008; Herrera et al., 2018).

In Simaroubaceae, the three major clades retrieved are broadly congruent with those found in previous studies, with some exceptions. We confirm that *Holacantha* and *Castela* are sister to the rest of the family (clade Si1, **Figure 2B**); however, *Picrasma* falls within a paraphyletic *Ailanthus*, in clade Si2 (**Figure 2B**; Clayton et al., 2007; Clayton et al., 2009; Clayton, 2011). This surprising result may be caused in part by the relatively high degree of paralogy in clade Si2 compared to the rest of the family, or the omission of *Leitneria*, and warrants further investigation. The rest of the family (in Si3) forms a monophyletic grade, potentially explaining the labile taxonomic history of Simaroubaceae and the difficulty in identifying morphological synapomorphies in this group (Pirani et al., 2022).

In Meliaceae, the major clades retrieved support a two-subfamily classification system, with clade M1 (**Figure 2B**) equivalent to Cedreloideae, and clades M2 and M3 (**Figure 2B**) comprising a monophyletic Melioideae (Muellner et al., 2003; Muellner et al., 2006). However, as in previous molecular analyses, our results suggest that the morphological tribal classification of Pennington and Styles (1975) is in need of revision, with only Aglaieae and Melieae retrieved as monophyletic (Muellner et al., 2003; Muellner et al., 2006; Muellner et al., 2008; Koenen et al., 2015). The resolution of Melioideae has been greatly improved with the Angiosperms353 loci, and the topology (particularly of tribes Trichillieae and Turraeae) differs from previous molecular studies, suggesting that Melioideae comprises two main subclades. Subclade M2 contains *Melia*, *Azadirachta* (from tribe Melieae) and *Owenia* and *Pterorhachis* (from tribe Trichilieae). The relationship of *Owenia* with Melieae was previously shown by Muellner et al. (2008); Koenen et al. (2015) and Muellner-Riehl et al. (2016), but the placement of *Pterorhachis* differs substantially from previous classifications. In Melioideae subclade M3, *Munronia* is sister to the rest of Melioideae, which includes the remainder of Trichilieae split across two clades, Turreae (which was paraphyletic in relation to one of the clades of Trichilieae), the monotypic Vavaeae, and a paraphyletic Guareae in relation to Aglaieae. It is notable that in Meliaceae, particularly in Melioideae, there is extreme variation degree of paralogy in Angiosperms353 loci. This extreme variation in paralogy is in line with cytological studies that found Meliaceae has the highest variation in chromosome numbers in Sapindales (with a maximum 2n = 360 in *Trichilia dregeana*, tribe Trichilieae), likely driven by repeated polyploidization events and occasional dysploidy (Guimarães and Forni-Martins, 2022). Although the influence of paralogy was reduced by the encoding of ambiguity characters in our sequences and our resulting tree is well-supported, we suggest that the extreme variation in paralogy may still affect the reconstructed topology and cause discordance with morphological taxonomic concepts, especially if the cause of paralogy is ancient hybridization (i.e., allopolyploidy). Examples of this could be in the surprising placements of *Pterorhachis* (which has low levels of paralogy relative to related genera) and *Munronia* (which has extremely high levels of paralogy for the order). Therefore, future phylogenetic and systematic studies on Meliaceae should focus on phasing gene copies to infer the type of gene duplication events that occurred in the evolutionary history of the family (i.e. autopolyploidization, allopolyploidization or duplication of certain regions), where they occurred, and how to reconstruct any reticulation events (e.g., Morales-Briones et al., 2021; Nauheimer et al., 2021).

The major clades retrieved in Rutaceae correspond broadly with the most recent subfamily classification (Appelhans et al., 2021): Rutoideae, Aurantoideae and the monotypic Haplophylloideae are monophyletic within clade R3 (**Figure 2C**). However, *Decatropis* and *Stauranthus* make Amyridoideae (within clade R3) and Zanthoxyloideae (most of R4; **Figure 2C**) non-monophyletic, with *Decatropis* falling in Amyridoideae instead of Zanthoxyloideae, and *Stauranthus* retrieved with Zanthoxyloideae instead of Amyridoideae. Both *Decatropis* and *Stauranthus* are small genera from Central America that have not been included in previous phylogenetic studies, and their assignment to these subfamilies should be further tested.

Most notably, our results suggest that Rutaceae subfamily Cneoroideae is polyphyletic, with genera split across two clades (R1 and R2; **Figure 2C**). Genera of clade R1 (**Figure 2C**) lack the characteristic glandular dots of typical Rutaceae (Appelhans et al., 2011), and until recently were assigned to Simaroubaceae (*Harrisonia*), Cneoraceae (*Cneorum*), and Ptaeroxylaceae (*Bottegoa*, *Cedrelopsis, Ptaeroxylon*). Likewise, genera of clade R2 (**Figure 2C**; *Dictyoloma*, *Sohnreyia, Spathelia*) were originally assigned to tribe Spathelieae of Simaroubaceae, based primarily on possession of staminal filament appendages and gynoecium structure, but were placed in Rutaceae by Engler (1931). The aforementioned genera (in clades R1 and R2 of our analysis) were recognised as a subfamily of Rutaceae based on weak to moderate phylogenetic support (Gadek et al., 1996; Chase et al., 1999; Groppo et al., 2008; Appelhans et al., 2011; Appelhans et al., 2021), similar biochemistry, and shared absence or restricted presence of schizogenous oil glands (Waterman, 1993; Waterman, 2007; Appelhans et al., 2021), although Groppo et al. (2008) noted that synapomorphies for the group were lacking. With the dense sampling and large number of loci included in our study, we have shown that Cneoroideae is not monophyletic and is in need of taxonomic revision. Furthermore, the divergence of the Cneoroideae clades (R1 and R2) is likely to be ancient, occurring in the Upper Cretaceous (or Lower Cretaceous, if an ancient angiosperm scenario is assumed), in parallel with the divergence of Anacardiaceae and Burseraceae. Therefore, in combination with uncertain morphological synapomorphies uniting these clades with core Rutaceae (Appelhans et al., 2011; Groppo et al., 2012), reinstating the families Cneoraceae and/or Ptaeroxylaceae may be warranted.

As in the Melioideae of Meliaceae (M2 & M3; **Figure 2B**), extreme variation in paralogy was observed across genera in Rutaceae, particularly in clade R4 (mostly comprising subfamily Zanthoxyloideae). This may be driving topological conflict between the phylogenies produced with concatenated and coalescent analyses (particularly in the placement of *Decazyx, Flindersia*, *Nematolepis*, *Peltostigma*, *Plethadenia*, and *Ptelea*), and conflicting support for relationships of certain genera that are incongruent with working hypotheses derived mainly from plastome-based molecular phylogenetic trees and morphology (e.g., the non-monophyly of *Boronia* and the placement of *Correa, Halfordia*, *Muiriantha*, and *Phebalium*). Chromosome number is known to vary substantially across Rutaceae lineages, with a genome duplication event hypothesised to have occurred early in the evolution of the family followed by multiple polyploidization events (Appelhans et al., 2012; Paetzold et al., 2018; Appelhans et al., 2021; Guimarães and Forni-Martins, 2022). Future studies investigating the nature of gene duplication events in Rutaceae should be undertaken to improve our understanding and reconstruction of the evolution of the family, particularly subfamily Zanthoxyloideae.

Dense sampling of Sapindales genera and sequencing of Angiosperms353 loci has confirmed the monophyly of the nine currently recognised families and improved resolution of their relationships, but also indicated that the recognition of the previously accepted families Cneoraceae and Ptaeroxylaceae, which are currently placed in Rutaceae (clade R1), may be warranted. Regardless of the crown age of the angiosperms, Sapindales is clearly an ancient order, and its families emerged rapidly. Our results support the idea that Mid-Cretaceous climate change drove the diversification of angiosperm families, showing that the Mid-Cretaceous Hothouse likely had a substantial impact on the evolution of Sapindales. If the angiosperms are assumed to be ancient, the rising temperatures and Cenomanian–Turonian Thermal Maximum of the Mid-Cretaceous Hothouse period could have been a key period for the diversification of Sapindales families that were already present; if the angiosperms are assumed to be younger, the rising temperatures of the Mid-Cretaceous Hothouse period may have been coincident with the emergence of Sapindales families, and the cooling temperatures following the Cenomanian–Turonian Thermal Maximum was likely coincident with family diversification. It would be interesting to corroborate these results by investigating how diversification dynamics change with climate change, and if it is more likely that crown nodes of families would coincide with climate minima (such as the start of the Mid-Cretaceous Hothouse) or maxima (such as the Cenomanian–Turonian Thermal Maximum). In most families, infra-familial classifications need some revision, and our analysis may give insight into why infra-familial classification is so difficult: signals of gene duplication are heterogeneously dispersed throughout the order, and are particularly strong in Rutaceae, Meliaceae and some Anacardioideae (Anacardiaceae). Taken with evidence from cytological studies and the complex morphological patterns in these clades (Pennington and Styles, 1975; Appelhans et al., 2012; Paetzold et al., 2018; Appelhans et al., 2021; Guimarães and Forni-Martins, 2022), it points to a complex evolutionary history potentially involving local gene duplication, ancient hybridization (allopolyploidization) and autopolyploidization. These processes, especially ancient hybridization, may affect our ability to reconstruct the evolution of these clades as a bifurcating tree and interpret morphology (Morales-Briones et al., 2021; Nauheimer et al., 2021; Heslop-Harrison et al., 2022). Therefore, investigation of the processes responsible for these signals of gene duplication will be critical for furthering our understanding of evolutionary relationships in these clades. Moreover, the heterogeneous signal of gene duplication across Sapindales is interesting in itself: why do some families and clades in Sapindales retain a signal of gene duplication in their genome, while others don’t? Thus, further investigation of the processes underlying gene duplication events may give key insight not only into the evolution of this ecologically and commercially important order, but angiosperm evolution more broadly.

## Data availability statement

The datasets generated for this study can be found in the NCBI’s Sequence Read Archive (SRA): https://www.ncbi.nlm.nih.gov/sra. Consensus sequences, alignments and tree files generated in this study are available at: doi: 10.5281/zenodo.7585555.

## Author contributions

EJ conceived the study, along with DC, KN, KT and PAFTOL Principal Investigators WB, FF and IL. Sampling was conducted by EJ, MA, SB, JV, JP and MB. OM helped to manage and source samples. EJ conducted laboratory work with assistance from AZ. EJ carried out all analyses with support from AZ and LN. HS provided expertise in dating analysis and access to the PROTEUS database. MA, SB, MC, JV, JP, JB, MB, MC, MD, SP, MG, PL, JMi, CS, JMi, HO, CP, AW and AM-R contributed family expertise. EJ wrote the manuscript, with input from MA, SB, MC, JV, JP, JB, MB, MC, MD, SP, MG, PL, JMi, CS, JMu, HO, CP, LN, HS, AM-R, FF, KN, KT, WB and DC. All authors contributed to the article and approved the submitted version. 

## Supplementary material

Supplementary Material 1 List of ingroup and outgroup samples included in the genus-level phylogenetic analysis of Sapindales and their NCBI SRA accession numbers.

Supplementary Material 2 Output of HybPhaser showing recovery statistics for loci, allele divergence, and the proportion of loci with more than 0% SNPs (hetereozygosity), 0.5% SNPs, 1% SNPs and 2% SNPs.

Supplementary Material 3 Details of each fossil used for time-calibration of the Sapindales tree.

Supplementary Material 4 Trees from the concatenated alignment showing the allele divergence of each sample, and their proportion of loci with more than 0%, 0.5%, 1% and 2% SNPs.

Supplementary Material 5 Coalescent species tree of Sapindales generated from cleaned gene trees using ASTRAL v5.7.8, with node support shown as posterior probability.

Supplementary Material 6
**Table 1** Ages and 95% HPD intervals for major clades in Sapindales under different dating schemes. ‘C.’ denotes crown node, ‘S.’ denotes stem node. KAB refers to major Sapindales clade with Kirkiaceae, Anacardiaceae and Burseraceae; SRM refers to major Sapindales clade with Simaroubaceae, Rutaceae and Meliaceae. **Figure 1** Comparison of chronograms estimated under a young-angiosperm (RC-complete; blue) and old-angiosperm (UC-complete; red) scenario. Thick lines mark the stem branch of Sapindales families for each dating analysis. Grey line under chronogram represents global average paleotemperature following Scotese (2021), with orange shading demarcating the Mid-Cretaceous Hothouse and asterisk (*) indicating the Cenomanian–Turonian Thermal Maximum.

Supplementary Material 7 Chronogram generated under the CC-complete dating analysis in BEAST with a uniform root prior set to 140.33-144.29, Birth-Death tree prior and uniform internal fossil priors. Ages and HPDs shown in red above nodes.

Supplementary Material 8 Chronogram generated under the RC-complete dating analysis in BEAST with a uniform root prior set to 143.91-147.94, Birth-Death tree prior and uniform internal fossil priors. Ages and HPDs shown in red above nodes.

Supplementary Material 9 Chronogram generated under the UC-complete dating analysis in BEAST with a uniform root prior set to 212.25-221.02, Birth-Death tree prior and uniform internal fossil priors. Ages and HPDs shown in red above nodes.

Supplementary Material 10 Sensitivity analysis showing chronogram generated under the RC-complete dating analysis in BEAST with a uniform root prior set to 143.91-147.94, Yule tree prior and uniform internal fossil priors. Ages and HPDs shown in red above nodes.

Supplementary Material 11 Sensitivity analysis showing chronogram generated under the RC-complete dating analysis in BEAST with a uniform root prior set to 143.91-147.94, Birth-Death tree prior and lognormal internal fossil priors. Ages and HPDs shown in red above nodes.
