# Supplementary material for: Phylogenomic analyses of Sapindales support new family relationships, rapid Mid-Cretaceous Hothouse diversification, and heterogeneous histories of gene duplication
Source: Front Plant Sci. 2023 Mar 7;14:1063174. doi: 10.3389/fpls.2023.1063174 (PMC10028101; doi:10.3389/fpls.2023.1063174)
Supplement: Supplementary Material 1 — List of ingroup and outgroup samples included in the genus-level phylogenetic analysis of Sapindales and their NCBI SRA accession numbers. [file DataSheet_1.zip › Supplementary Material/Supplementary Material 1.pdf]

Supplementary file 1 List of ingroup and outgroup samples included in the genus-level phylogenetic analysis of Sapindales.

Family	Species	Material type	Collector	Voucher	SRA ID
Anacardiaceae	*Abrahamia ditimena*	DNA	Randrianasolo 781	MO	ERR7620092
Anacardiaceae	*Actinocheita filicina*	Herbarium	Salinas 7503	K	ERR7619456
Anacardiaceae	*Allospondias lakonensis*	DNA	Pell 1035	NY (BKL)	ERR7620093
Anacardiaceae	*Amphipterygium adstringens*	DNA	Pendry 845	E	ERR7620094
Anacardiaceae	*Anacardium occidentale*	Herbarium	McKenna, S.G. 363	CNS131081	ERR7619987
Anacardiaceae	*Apterokarpos gardneri*	DNA	Pirani 2586	NY	ERR7620095
Anacardiaceae	*Baronia taratana*	DNA	Pell 625	NY	ERR7620097
Anacardiaceae	*Blepharocarya involucrigera*	DNA	R. Jensen 826	A	ERR7620098
Anacardiaceae	*Bonetiella anomala*	DNA	Johnston, Wendt & Chiang 11488	F	ERR7620099
Anacardiaceae	*Bouea oppositifolia*	Herbarium	Saw, L.G. FRI48291	CNS14338	ERR7619990
Anacardiaceae	*Buchanania obovata*	Silica	Wannan, B.S. 6674	CNS141235	ERR7619978
Anacardiaceae	*Campnosperma zeylanicum*	Herbarium		K; K DNA bank no. 6369	ERR7618705
Anacardiaceae	*Campylopetalum siamense*	DNA	Garrett 1398	NY	ERR7620100
Anacardiaceae	*Cardenasiodendron brachypterum*	DNA	Pendry 691	E	ERR7620101
Anacardiaceae	*Choerospondias axillaris*	DNA	JD Mitchell 652	NY	ERR7620102
Anacardiaceae	*Cotinus coggygria*	SRA			SRR829833
Anacardiaceae	*Cotinus obovatus*	Silica	Joyce & Maurin	K; K living collection no. 1969-12584	ERR7620168
Anacardiaceae	*Cyrtocarpa caatingae*	Herbarium	Ule 7256	K	ERR7619455
Anacardiaceae	*Cyrtocarpa procera*	DNA	Torres 124	NY	ERR7620103
Anacardiaceae	*Dobinea vulgaris*	DNA	Delendick 617	NY	ERR7620104
Anacardiaceae	*Dracontomelon duperreanum*	DNA	SK Pell 1034	NY	ERR7620105
Anacardiaceae	*Drimycarpus racemosus*	DNA	Grierson/Long 4261	A	ERR7620106
Anacardiaceae	*Euroschinus papuana*	Herbarium	Takeuchi, W.; Ama, D. 17126	K000618182	ERR7619414
Anacardiaceae	*Faguetia falcata*	DNA	Pell, S. 600	NY	ERR7620107
Anacardiaceae	*Fegimanra africana*	DNA	Reitsma & Reitsma 1257	NY	ERR7620108
Anacardiaceae	*Gluta renghas*	Herbarium	Chase, M.W. 2066	K; K DNA bank no. 1953	ERR7618706

Anacardiaceae	*Haplorhus peruviana*	DNA	O. Zöllner 403	L	ERR7620109
Anacardiaceae	*Harpephyllum caffrum*	DNA	Lau 1588	NY	ERR7620110
Anacardiaceae	*Heeria argentea*	DNA	Chase, M.W. 6588	K DNA bank no. 4855	ERR7620186
Anacardiaceae	*Laurophyllus capensis*	DNA	Manning, J.C. & Laura 2906	K DNA bank no. 29002	ERR7620212
Anacardiaceae	*Loxopterygium grisebachii*	DNA	Cadrera *et al.* 27543	K DNA bank no. 17824	ERR7620189
Anacardiaceae	*Loxostylis alata*	DNA	C. Pinheiro 79	NY	ERR7620111
Anacardiaceae	*Mangifera indica*	SRA			SRR1562187
Anacardiaceae	*Mangifera odorata*	DNA		CNS	ERR7619992
Anacardiaceae	*Mauria simplicifolia*	DNA	Leiva *et al.* 1552	F	ERR7620112
Anacardiaceae	*Melanochyla caesia*	DNA	Ambriansyah & Arifin 903	MO	ERR7620113
Anacardiaceae	*Metopium brownei*	DNA	Brokaw 295	NY	ERR7620115
Anacardiaceae	*Micronychia macrophylla*	DNA	Pell 643	NY	ERR7620116
Anacardiaceae	*Mosquitoxylum jamaicense*	DNA	Rodríguez 736	MO	ERR7620117
Anacardiaceae	*Myracrodruon urundeuva*	DNA	Pendry 724	E	ERR7620118
Anacardiaceae	*Myracrodruon balansae*	DNA	Anderson, W.R. 13597	MICH1460619; K DNA bank no. 21936	ERR7620145
Anacardiaceae	*Nothopegia beddomei*	DNA	Samuel, R.	K DNA bank no. 21392	ERR7620198
Anacardiaceae	*Ochoterenaea colombiana*	DNA	Escobar, Folsom, Brand & Sánchez 2598	F	ERR7620119
Anacardiaceae	*Operculicarya decaryi*	DNA	Randrianasolo 627	MO	ERR7620120
Anacardiaceae	*Orthopterygium huaucui*	DNA	Smith 5726	NY	ERR7620121
Anacardiaceae	*Ozoroa insignis*	DNA	Randrianasolo 68	MO	ERR7620122
Anacardiaceae	*Pachycormus discolor*	DNA	Chase, M.W. 3074	K DNA bank no. 2873	ERR7620164
Anacardiaceae	*Parishia insignis*	Silica	Bachelier	NA	ERR7620123
Anacardiaceae	*Pegia nitida*	DNA	Zhanhuo 92-254	MO	ERR7620124
Anacardiaceae	*Pistacia chinensis*	SRA			SRR8661763
Anacardiaceae	*Pistacia lentiscus*	SRA			SRR5043662
Anacardiaceae	*Pleiogynium timoriense*	Herbarium	Chase, M.W. 2069	K; K DNA bank no. 1956	ERR7618707

Anacardiaceae	*Poupartia borbonica*	Silica	Joyce & Maurin	K; K living collection no. 2010-2013	ERR7620170
Anacardiaceae	*Poupartia chapelieri*	Herbarium	Dumetz 1294	K001275567	ERR7620410
Anacardiaceae	*Poupartia minor*	DNA	Pell 657	NY	ERR7620127
Anacardiaceae	*Poupartiopsis spondiocarpus*	DNA	Randrianasolo 592	MO	ERR7620128
Anacardiaceae	*Protorhus longifolia*	DNA	R.Brand 322	NY	ERR7620129
Anacardiaceae	*Pseudosmodingium andrieuxii*	DNA	Tenorio 17041	F	ERR7620131
Anacardiaceae	*Pseudospondias microcarpa*	DNA	RA 809	MO	ERR7620130
Anacardiaceae	*Rhodosphaera rhodanthema*	Herbarium	Forster, P.I.; Booth, R. 25343	QRS128713	ERR7619986
Anacardiaceae	*Rhus coriaria*	DNA	E. Vitek 2000-301	W	ERR7620132
Anacardiaceae	*Rhus perrieri*	DNA	Randrianasolo 629	MO	ERR7620133
Anacardiaceae	*Rhus taitensis*	DNA	Pell 841	NY	ERR7620114
Anacardiaceae	*Rhus thouarsii*	DNA	Pell 638	NY	ERR7620134
Anacardiaceae	*Schinopsis brasiliensis*	DNA	Bridgewater 1012	E	ERR7620135
Anacardiaceae	*Schinus patagonica*	DNA	Kepos 2785	K DNA bank no. 28228	ERR7620211
Anacardiaceae	*Sclerocarya birrea*	DNA	SKP 695	NY (BKL)	ERR7620136
Anacardiaceae	*Searsia undulata*	DNA	SKP 692	NY (BKL)	ERR7620137
Anacardiaceae	*Semecarpus papuanus*	DNA	Johns, R.J. 9795	K000260707; K DNA bank no. 18116	ERR7620190
Anacardiaceae	*Smodingium argutum*	DNA	Winter 88	MOR	ERR7620138
Anacardiaceae	*Sorindeia madagascariensis*	DNA	Randrianasolo 653	MO	ERR7620139
Anacardiaceae	*Spondias mombin*	Herbarium	Ameka *et al.* 213	GC; K DNA bank no. 40825	ERR7618708
Anacardiaceae	*Swintonia schwenckii*	DNA	C. Herscovitch s.n.	NY	ERR7620140
Anacardiaceae	*Tapirira guianensis*	DNA	Daly 13984	NY	ERR7620141
Anacardiaceae	*Thyrsodium spruceanum*	DNA	S. Mori 24215	NY	ERR7620142
Anacardiaceae	*Toxicodendron pubescens*	DNA	J.D. Mitchell 1501	NY	ERR7620143
Anacardiaceae	*Toxicodendron radicans*	SRA			ERR2040463
Anacardiaceae	*Toxicodendron vernicifluum*	SRA			SRR5858884
Anacardiaceae	*Trichoscypha longifolia*	Silica	Burgt, X.M. van der 1856	K001061794; K DNA bank no. 73151	ERR7620209

Biebersteiniaceae	*Biebersteinia odora*	DNA	Holubec	K DNA bank no. 22402	ERR4180115
Burseraceae	*Aucoumea klaineana*	Herbarium	J.J.F.E. de Wilde *et al.* 11307	K001275407	ERR7620223
Burseraceae	*Beiselia mexicana*	DNA	Chase, M.W. 639	K DNA bank no. 158	ERR7620152
Burseraceae	*Boswellia sacra*	DNA	Chase, M.W. 34495	K DNA bank no. 3495	ERR7620214
Burseraceae	*Bursera fagaroides*	Silica		K living collection no. 1989-2554	ERR7620174
Burseraceae	*Bursera simaruba*	SRA			ERR2040465
Burseraceae	*Canarium muelleri*	Silica	Howard, M. MGH244	BRI1014904	SRR22164185
Burseraceae	*Commiphora habessinica*	DNA	Chase, M.W. 526	K DNA bank no. 16	ERR7620149
Burseraceae	*Dacryodes laxa*	DNA	Chase, M.W. 2087	K DNA bank no. 1974	ERR7620158
Burseraceae	*Garuga floribunda*	Herbarium	Hyland, B. 11522	QRS67790	SRR22164184
Burseraceae	*Haplolobus floribundus*	Herbarium	Kanis, A. 1108	K000062329	ERR7619415
Burseraceae	*Protium altissimum*	Herbarium	Zappi, D. 1250	K000578064	ERR7620226
Burseraceae	*Protium copal*	SRA			SRR9107802
Burseraceae	*Protium javanicum*	Herbarium	Chase, M.W. 2089	K; K DNA bank no. 1976	ERR7618709
Burseraceae	*Protium rhoifolium*	Herbarium	Miralha 46	K001275409	ERR7620225
Burseraceae	*Trattinnickia rhoifolia*	Herbarium	Mori & Gracie sn	K00127541	ERR7620227
Burseraceae	*Triomma malaccensis*	DNA	Chase, M.W. 2091	K DNA bank no. 1978	ERR7620159
Caricaceae	*Cylicomorpha parviflora*	SRA			ERR4180126
Combretaceae	*Terminalia neotaliala*	SRA			ERR2040313
Crossosomataceae	*Crossosoma californicum*	SRA			SRR7451089
Cyrillaceae	*Cyrilla racemiflora*	SRA			SRR7451102
Dipentodontaceae	*Perrottetia longistylis*	SRA			ERR4297056
Dipterocarpaceae	*Anisoptera marginata*	SRA			ERR4180094
Dipterocarpaceae	*Monotes glaber*	SRA			ERR4180058
Emblingiaceae	*Emblingia calceoliflora*	SRA			ERR4180003
Fabaceae	*Acacia pycnantha*	SRA			ERR2040344
Fabaceae	*Gilbertiodendron ecoukense*	SRA			SRR7451091
Francoaceae	*Francoa appendiculata*	SRA			ERR2040311

Francoaceae	*Greyia sutherlandii*	SRA			ERR2040312
Geraniaceae	*Geranium carolinianum*	SRA			ERR2040309
Geraniaceae	*Geranium maculatum*	SRA			ERR204031
Kirkiaceae	*Kirkia acuminata*	Silica	Appelhans MA393	GOET	ERR7620080
Kirkiaceae	*Kirkia wilmsii*	SRA			ERR2040467
Koeberliniaceae	*Koeberlinia spinosa*	SRA			ERR4180172
Malvaceae	*Gossypium barbadense*	SRA			SRR8878749
Melastomataceae	*Medinilla magnifica*	SRA			ERR2040321
Meliaceae	*Aglaia elaeagnoidea*	Silica	Ford, A. 5776	CNS134440	SRR21798412
Meliaceae	*Aglaia macrocarpa*	Silica	Trethowan 608	K	ERR7620411
Meliaceae	*Aglaia odorata*	SRA			SRR5947159
Meliaceae	*Aglaia tomentosa*	DNA	Greger, H. 689	K DNA bank no. 18158	ERR7620191
Meliaceae	*Anthocarapa nitidula*	Silica	Costion, C. 1335	CNS131163	ERR7619982
Meliaceae	*Aphanamixis polystachya*	DNA	Middleton, D.J. *et al.* 1329	K DNA bank no. 41973	ERR7620218
Meliaceae	*Astrotrichilia asterotricha*	Herbarium	P.B. Phillipson *et al.* 6139	K	ERR7620089
Meliaceae	*Azadirachta indica*	Silica	McDonald, K.R. 10191	CNS137932	ERR7619979
Meliaceae	*Cabralea canjerana*	DNA	Wasum, R. 1196	K DNA bank no. 19711	ERR7620194
Meliaceae	*Calodecaryia crassifolia*	DNA	Croat, T.B. 31521	K DNA bank no. 19701	ERR7620192
Meliaceae	*Capuronianthus mahafalensis*	DNA	Cheek *et al.* 3254	K DNA bank no. 3607	ERR7620181
Meliaceae	*Carapa procera*	DNA	Senterre, B. & Obiang, D. 4243	K DNA bank no. 26675	ERR7620202
Meliaceae	*Cedrela angustifolia*	DNA	Muellner, A.N. 2054	K DNA bank no. 21127	ERR7620197
Meliaceae	*Cedrela odorata*	SRA			SRR7505213
Meliaceae	*Chisocheton longistipitatus*	Herbarium	Ford, A. 4730	QRS128685	SRR21798422
Meliaceae	*Chukrasia tabularis*	Herbarium	Meijer 32	K001275564	ERR7620405
Meliaceae	*Cipadessa baccifera*	DNA	Chase, M.W. 249	K DNA bank no. 22012	ERR7620146
Meliaceae	*Didymocheton* sp.	DNA	Chase, M.W. 1311	K DNA bank no. 576	SRR22164179
Meliaceae	*Ekebergia capensis*	DNA	Chase, M.W. 5807	K DNA bank no. 409	ERR7620185

Meliaceae	*Entandrophragma bussei*	Herbarium	Bidgood *et al.* 1156	K001275565	ERR7620406
Meliaceae	*Heckeldora staudtii*	DNA	Senterre, B., Obiang, D. & Esono, P. 2874	K DNA bank no. 26681	ERR7620204
Meliaceae	*Heynea trijuga*	DNA	Boyce 87	K DNA bank no. 48352	ERR7620222
Meliaceae	*Humbertioturraea grandidieri*	Herbarium	C.C.H. Jongkind *et al.* 3605	K	ERR7620090
Meliaceae	*Khaya anthotheca*	DNA	Chase, M.W. 2859	K DNA bank no. 2666	ERR7620162
Meliaceae	*Lepidotrichilia volkensii*	Herbarium	I. Friis *et al.* 9816	K	ERR7620091
Meliaceae	*Leplaea laurentii*	DNA	Evrard, C. 3286	K DNA bank no. 41997	ERR7620221
Meliaceae	*Lovoa swynnertonii*	DNA	Faden & Evans 70122	K DNA bank no. 2667	ERR7620163
Meliaceae	*Malleastrum mandenense*	DNA	Cheek *et al.* 3175	K DNA bank no. 3608	ERR7620182
Meliaceae	*Melia azedarach* 1	DNA	Leon, C.J., Li Bao-Li, Huang & Zhou 350	K; K DNA bank no. 37230	ERR7620215
Meliaceae	*Melia azedarach* 2	SRA			ERR2040470
Meliaceae	*Munronia pinnata*	Herbarium	Ridsdale, C.E. 281	K001275561	ERR7620409
Meliaceae	*Naregamia alata*	DNA	K.C.Kanodia 89603	K DNA bank no. 3591	ERR7620178
Meliaceae	*Neobeguea* sp.	DNA	Cheek *et al.* 3251	K DNA bank no. 3609	ERR7620183
Meliaceae	*Neoguarea glomerulata*	DNA	Senterre, B. & Obiang, D. 365	K DNA bank no. 2668	ERR7620203
Meliaceae	*Nymania capensis*	DNA	Chase, M.W. 27	K DNA bank no. 22033	ERR7620148
Meliaceae	*Owenia reticulata*	Silica	Jensen, R. 1851	CNS139403	ERR7619980
Meliaceae	*Prasoxylon alliaceum*	Silica	Costion, C. 3017	CNS135374	SRR21798415
Meliaceae	*Pseudobersama mossambicensis*	DNA	Bidgood, Abdallah & Vollesen 1426	K DNA bank no. 3595	ERR7620179
Meliaceae	*Pseudocedrela kotschyi*	DNA	Asase 8	K DNA bank no. 17369	ERR7620188
Meliaceae	*Pseudoclausena chrysogyne*	Herbarium	Muellner, A.N. *et al.* 2052	K; K DNA bank no. 19709	ERR7618710
Meliaceae	*Pterorhachis zenkeri*	DNA	F.J.Breteler 2741	K DNA bank no. 359	ERR7620177
Meliaceae	*Quivisianthe papinae*	DNA	Cheek *et al.* 3192	K DNA bank no. 361	ERR7620184

Meliaceae	*Reinwardtiodendron celebicum*	Herbarium	Kostermans, A.J.G.H 13776	K; K DNA bank no. 16381	ERR7618711
Meliaceae	*Ruagea glabra*	DNA	Marten & Herrera 68	K DNA bank no. 25687	ERR7620200
Meliaceae	*Schmardaea microphylla*	Herbarium	Lewis & Lozano 3034	K001275563	ERR7620407
Meliaceae	*Soymida febrifuga*	Herbarium	Meijer *et al.* 637	K001275562	ERR7620408
Meliaceae	*Swietenia macrophylla*	DNA	Chase, M.W. 25	K DNA bank no. 22013	ERR7620147
Meliaceae	*Synoum glandulosum*	DNA	Schodde, R. 5101	K DNA bank no. 19704	ERR7620193
Meliaceae	*Toona ciliata*	Silica	Shapcott, A. Toi2-1	CNS143714	SRR22164178
Meliaceae	*Toona sinensis*	SRA			SRR847482
Meliaceae	*Trichilia hirta*	DNA	Wallnofer *et al.* 9676	K DNA bank no. 25639	ERR7620199
Meliaceae	*Turraea virens*	Herbarium	Ralimanana	K; K DNA bank no. 15733	ERR7618712
Meliaceae	*Turraeanthus mannii*	DNA	Cheek, M. 8058	K DNA bank no. 3596	ERR7620180
Meliaceae	*Vavaea amicorum*	Herbarium	Curry, P. 1614	K; K DNA bank no. 48348	ERR7618713
Meliaceae	*Walsura tubulata*	Herbarium	Chase, M.W. 1314	K; K DNA bank no. 585	ERR7618714
Meliaceae	*Xylocarpus moluccensis*	Herbarium	Puradyatmika 10417	K; K DNA bank no. 19996	ERR7618715
Nitrariaceae	*Malacocarpus crithmifolius*	DNA	Sheahan, M-C.	K DNA bank no. 916	ERR7620151
Nitrariaceae	*Nitraria retusa*	DNA	Chase, M.W. 597	K DNA bank no. 918	ERR7620150
Nitrariaceae	*Nitraria sibirica*	SRA			SRR7013771
Nitrariaceae	*Nitraria tangutorum*	SRA			SRR1768462
Nitrariaceae	*Peganum harmala*	DNA	Chase, M.W. 38653.19685	K DNA bank no. 39167	ERR7620216
Onagraceae	*Oenothera rosea*	SRA			ERR706864
Polygalaceae	*Polygala lutea*	SRA			ERR706838
Rutaceae	*Acmadenia kiwanensis*	DNA	JEV 2405	K DNA bank no. 26932	ERR7620207
Rutaceae	*Acronychia laevis*	Silica	Worboys, S.J. 1350	CNS144810	SRR22164177
Rutaceae	*Acronychia octandra*	Herbarium	Forster PIF14620	K001275568	ERR7620412

Rutaceae	*Acronychia pedunculata*	DNA	Chase, M.W. 313	NCU562141; K DNA bank no. 22076	ERR7618716
Rutaceae	*Adenandra uniflora*	DNA	Chase, M.W. 1769	K DNA bank no. 1578	ERR7620157
Rutaceae	*Adiscanthus fusciflorus*	Herbarium	Groppo *et al.* 953	K001177833	ERR7620228
Rutaceae	*Aegle marmelos*	SRA			SRR7268533
Rutaceae	*Aeglopsis chevalieri*	Herbarium	Cheek *et al.* 13878	K000615634	ERR7620229
Rutaceae	*Afraegle asso*	Silica	Tchiengue, B. 3788	K001243700; K DNA bank no. 88054	ERR7620210
Rutaceae	*Agathosma gonaquensis*	Silica	Joyce & Maurin	K; K living collection no. 2008-1399	ERR7620173
Rutaceae	*Amyris elemifera*	Herbarium	Breckle 10844	GOET	ERR7619993
Rutaceae	*Andreadoxa flava*	Herbarium	dos Santos, T.S. *et al.* 4588	K001173105	ERR7620359
Rutaceae	*Angostura bracteata*	DNA	Pirani *et al.* 357	K DNA bank no. 671	ERR7620187
Rutaceae	*Asterolasia asteriscophora*	Silica	Bayly, M. MJB2564	MELUD114862	ERR7599574
Rutaceae	*Atalantia ceylanica*	Herbarium	Chase, M.W. 1341	K; K DNA bank no. 727	ERR7618717
Rutaceae	*Balfourodendron riedelianum*	Herbarium	Silva, S.M. & Ziller, S.R. 1889	K00117391	ERR7620360
Rutaceae	*Balsamocitrus dawei*	Herbarium	Dawe, M.J. 1042	K000199535	ERR7620361
Rutaceae	*Bergera koenigii*	Silica		K living collection no. 2013-568	ERR7620169
Rutaceae	*Boenninghausenia albiflora*	Silica	Appelhans MA574	GOET	ERR7620063
Rutaceae	*Boronia imlayensis*	Silica	Bayly, M. MJB2005	MELU105861	ERR7599570
Rutaceae	*Boronia parvifolia*	Herbarium	Munzinger & McPherson 813	MO	ERR7620081
Rutaceae	*Bosistoa medicinalis*	Silica	Sankowsky, G. 4001	CNS14299	ERR7619981
Rutaceae	*Bottegoa insignis*	DNA	Fris, Vollesen & Hassan 4824	K DNA bank no. 2087	ERR7620161
Rutaceae	*Bouchardatia neurococca*	Herbarium	Sankowsky,G. Sanko2949	MELUD105886	ERR7599608
Rutaceae	*Brombya platynema*	DNA	Ford AF4819	L	ERR7620082
Rutaceae	*Burkillanthus malaccensis*	Herbarium	Jones & Kusen 3287	K00127553	ERR7620362
Rutaceae	*Calodendrum capense*	Herbarium	Chapman *et al.* 6034	K001275531	ERR7620363
Rutaceae	*Casimiroa edulis*	Herbarium	Estrada *et al.* 5035	K001275532	ERR7620364
Rutaceae	*Cedrelopsis gracilis*	DNA	Randrianarivelojosia	TAN, DNA sample L	ERR7620083

Rutaceae	*Chloroxylon swietenia*	DNA	Chase, M.W. 1291	K; K DNA bank no. 398	SRR22164176
Rutaceae	*Chorilaena quercifolia*	Silica	Bayly, M. MJB1954	MEL 2383575	ERR7599571
Rutaceae	*Citropsis articulata*	Herbarium	Bidgood *et al.* 4797	K001275533	ERR7620365
Rutaceae	*Citrus* x *aurantium*	SRA			ERR760725
Rutaceae	*Citrus hystrix*	Silica		GOET	ERR7620065
Rutaceae	*Citrus japonica*	SRA			SRR6357068
Rutaceae	*Clausena excavata*	Herbarium	Chase, M.W. 1343	K; K DNA bank no. 729	ERR7618718
Rutaceae	*Cneoridium dumosum*	Silica	Freund 76	RSA	ERR7620066
Rutaceae	*Cneorum tricoccon*	Silica		GOET	ERR7620067
Rutaceae	*Coatesia paniculata*	Herbarium	Forster PIF29113	GOET	ERR7619994
Rutaceae	*Coleonema pulchellum*	Silica	Joyce & Maurin	K; K living collection no. 2010-742	ERR7620171
Rutaceae	*Comptonella microcarpa*	DNA	Porter P. Lowry Iwe 5734	MO	ERR7620084
Rutaceae	*Conchocarpus macrophyllus*	Herbarium	Pirani *et al.* 6078	K001266141	ERR7620366
Rutaceae	*Correa reflexa var. speciosa*	Silica		GOET living collection	ERR7620068
Rutaceae	*Crowea exalata*	Silica		K; K living collection no. 2016-102	ERR7620175
Rutaceae	*Decagonocarpus oppositifolius*	Herbarium	Maas *et al.* 6887	K001275534	ERR7620367
Rutaceae	*Decatropis bicolor*	Herbarium	de Nova *et al.* AN 451	K000341813	ERR7620368
Rutaceae	*Decazyx macrophyllus*	Herbarium	Estrada & Vega 2415	K001275535	ERR7620369
Rutaceae	*Dictamnus albus*	Silica	Appelhans MA576	GOET	ERR7620069
Rutaceae	*Dictyoloma vandellianum*	DNA	Appelhans MA381	L	ERR7620085
Rutaceae	*Dinosperma erythrococca*	Silica	Kilgour, C.D. 523	CNS135407	ERR7619983
Rutaceae	*Diosma sabulosa*	DNA	JEV 2409	K DNA bank no. 26929	ERR7620205
Rutaceae	*Drummondita hassellii*	DNA	Chase, M.W. 2188	K DNA bank no. 1903	ERR7620160
Rutaceae	*Dutaillyea trifoliolata*	Herbarium	Bernardi, L. LB 1260	K001275536	ERR7620370
Rutaceae	*Empleurum unicapsularis*	Herbarium	Cowell *et al.* 4057	K001275537	ERR7620373
Rutaceae	*Ertela trifolia*	Herbarium	Sasaki *et al.* 1535	K000447255	ERR7620371

Rutaceae	*Erythrochiton brasiliensis*	Silica	Joyce & Maurin	K; K living collection no. 1966-20101	ERR7620167
Rutaceae	*Euchaetis albertiniana*	DNA	JEV 2408	K DNA bank no. 2693	ERR7620206
Rutaceae	*Euodia hortensis*	Herbarium	Drake 235	US	ERR7620055
Rutaceae	*Euodia hylandii*	Herbarium	Craven, L.A. 10017	CANB498131	SRR22164175
Rutaceae	*Fagaropsis* sp.	Herbarium	Burger 3054	US	ERR7620056
Rutaceae	*Flindersia pimenteliana*	Herbarium	Hartley, T.G. 10493	K000062298	ERR7619416
Rutaceae	*Galipea ciliata*	Herbarium	Guedes *et al.* 5155	K001177777	ERR7620414
Rutaceae	*Galipea trifoliata*	Herbarium	Sasaki *et al.* 2063	K00044791	ERR7620372
Rutaceae	*Geijera linearifolia*	Herbarium	Smith, R.J., Hopper, S.D., Sweedman, L. 267	K; K DNA bank no. 37682	ERR7618719
Rutaceae	*Geleznowia verrucosa*	Silica	Mole, B. BM344	NSW1003545	SRR22164174
Rutaceae	*Glycosmis pentaphylla*	Herbarium	Chase, M.W. 1345	K; K DNA bank no. 731	ERR7618720
Rutaceae	*Halfordia kendack*	Silica	Forster, P. PIF34073	AQ0743506 (BRI)	SRR22164173
Rutaceae	*Haplophyllum bungei*	Silica	Manafzadeh 207	Z	ERR7620071
Rutaceae	*Harrisonia abyssinica*	Silica	Appelhans MA313	L	ERR7620072
Rutaceae	*Harrisonia brownii*	Herbarium	Dunlop, C., Cowie, I. 9451	K; K DNA bank no. 37057	ERR7618722
Rutaceae	*Helietta glaziovii*	Herbarium	Pirani *et al.* 5325	K001173941	ERR7620374
Rutaceae	*Hortia superba*	Herbarium	Groppo *et al.* 95	K001173896	ERR7620375
Rutaceae	*Ivodea decaryana*	Herbarium	Andriamihajarivo 296	MO	ERR7620057
Rutaceae	*Leionema ellipticum*	Silica	Ford, A. 2262	CNS	ERR7619984
Rutaceae	*Limonia acidissima*	Herbarium	Lau, J. SING 2009-272	K001275539	ERR7620377
Rutaceae	*Limnocitrus littorale*	Herbarium	Clemens & Clemens 3263	K001275538	ERR7620376
Rutaceae	*Lubaria aroensis*	Herbarium	Rodriguez 293	K00127554	ERR7620378
Rutaceae	*Lunasia amara*	Herbarium	Chase, M.W. 1347	K; K DNA bank no. 733	ERR7618723
Rutaceae	*Luvunga monophylla*	Herbarium	Ford, A. 6211	CNS14029	ERR7619991
Rutaceae	*Macrostylis decipiens*	Herbarium	Trinder-Smith 663	K001275542	ERR7620380
Rutaceae	*Medicosma cunninghamii*	Herbarium		K; K DNA bank no. 2152	ERR7618724
Rutaceae	*Merillia caloxylon*	Herbarium	Tokilip 143526	K000270699	ERR7620383

Rutaceae	*Melicope broadbentiana*	Herbarium	Ford, A. CL1A-2	CNS146709	SRR22164172
Rutaceae	*Melicope ternata*	Silica	Appelhans MA487	GOET	ERR7620073
Rutaceae	*Merope angulata*	Herbarium	Murata *et al.* J-486	K001275544	ERR7620382
Rutaceae	*Metrodorea nigra*	DNA	Kallunki et al 57	K DNA bank no. 1465	ERR7620155
Rutaceae	*Monanthocitrus oblanceolata*	Herbarium	Sinanggul, H. 57242	K001275545	ERR7620384
Rutaceae	*Muiriantha hassellii*	Herbarium	Thiele, K.R. 4549	PERTH 0870051	ERR7620423
Rutaceae	*Murraya exotica*	Herbarium	Lin Y-L.J. 91	K; K DNA bank no. 39631	ERR7618725
Rutaceae	*Myrtopsis macrocarpa*	Herbarium	Van Balgooy 6955	L	ERR7620058
Rutaceae	*Nematolepis squamea*	Herbarium	Visoiu & Kerr 708	K001275546	ERR7620385
Rutaceae	*Neobyrnesia suberosa*	Silica	Bayly, M. MJB1904	MEL2383567	ERR7599576
Rutaceae	*Neoraputia alba*	DNA	J.Kallunki *et al.* 611	K DNA bank no. 1477	ERR7620156
Rutaceae	*Orixa japonica*	Silica		GOET	ERR7620074
Rutaceae	*Peltostigma guatemalense*	Herbarium	Jimenez *et al.* 71	K001275549	ERR7620388
Rutaceae	*Pentaceras australe*	Herbarium	Forster PIF28897	NY	ERR7620059
Rutaceae	*Phebalium tuberculosum*	Silica	Mole, B. BJM375	NSW1003583	ERR7599572
Rutaceae	*Phellodendron amurense*	Silica		GOET	ERR7620075
Rutaceae	*Philotheca angustifolia*	Silica	Bayly, M. MJB1990	MEL2383589	ERR7599573
Rutaceae	*Phyllosma capensis*	Herbarium	Oliver 4316	K00127555	ERR7620389
Rutaceae	*Picrella glandulosa*	Herbarium	HS McKee 3189	US	ERR7620060
Rutaceae	*Pilocarpus pennatifolius*	Silica	Joyce & Maurin	K; K living collection no. 1969-17371	ERR7620176
Rutaceae	*Pitaviaster haplophyllus*	DNA	Ford 4821	L	ERR7620086
Rutaceae	*Pleiospermium alatum*	Herbarium	Kostermans 25109	K001275551	ERR7620390
Rutaceae	*Plethadenia granulata*	Herbarium	B Peguero, T Zanoni & E Soto 4025	NY	ERR7620061
Rutaceae	*Psilopeganum sinense*	Silica	Wen 12478	US	ERR7620076
Rutaceae	*Ptaeroxylon obliquum*	DNA	Fay, M.F.	K DNA bank no. 14714	ERR9229981
Rutaceae	*Ptelea trifoliata*	Silica		GOET	ERR7620077
Rutaceae	*Raputia heptaphylla*	Herbarium	Herrera 8783	K001275552	ERR7620391
Rutaceae	*Raputia ulei*	Herbarium	Cid Ferreira 5767	K001177894	ERR7620393

Rutaceae	*Raputiarana subsigmoidea*	Herbarium	Sasaki *et al.* 250	K001173096	ERR7620392
Rutaceae	*Rauia nodosa*	Herbarium	Pirani *et al.* 4685	K001177896	ERR7620394
Rutaceae	*Ravenia infelix*	DNA	J.Kallunki *et al.* 614	K DNA bank no. 1464	ERR7620154
Rutaceae	*Raveniopsis ruellioides*	Herbarium	Mutchinck *et al.* 64	K001275553	ERR7620396
Rutaceae	*Rhadinothamnus rudis*	Silica	Dixon, K. W. 1015	K; K DNA bank no. 36062	ERR7620208
Rutaceae	*Ruta angustifolia*	SRA			SRR790086
Rutaceae	*Ruta graveolens*	Silica	Appelhans MA578	GOET	ERR7620078
Rutaceae	*Sarcomelicope simplicifolia*	Silica	Joyce & Maurin	K; K living collection no. 1985-794	ERR7620165
Rutaceae	*Sheilanthera pubens*	Herbarium	Williams 2122	K001275569	ERR7620413
Rutaceae	*Skimmia japonica*	Silica		GOET	ERR7620079
Rutaceae	*Sohnreyia terminalioides*	DNA	A. Gentry *et al.*, 31751	MO, DNA sample L	ERR7620087
Rutaceae	*Spathelia splendens*	DNA	P Vásquez 2009-2	HAC, L	ERR7620088
Rutaceae	*Spiranthera speciosa*	Herbarium	Pirani *et al.* 3659	K001240038	ERR7620386
Rutaceae	*Stauranthus perforatus*	Herbarium	Thomsen 82	K00127557	ERR7620415
Rutaceae	*Swinglea glutinosa*	Herbarium	Hodgson Lowe 4342	K001275571	ERR7620416
Rutaceae	*Tetractomia tetrandra*	Herbarium	Brambach 1472	GOET	ERR7620062
Rutaceae	*Tetradium daniellii*	Silica	Joyce & Maurin	K; K living collection no. 2019-1434	ERR7620166
Rutaceae	*Thamnosma texana*	Herbarium	Halse 4427	K001275554	ERR7620397
Rutaceae	*Ticorea foetida*	Herbarium	Jansen-Jacobs 6800	K001275572	ERR7620417
Rutaceae	*Toxosiphon lindenii*	Herbarium	Estrada & Vega 2417	K001275573	ERR7620418
Rutaceae	*Triphasia brassii*	Herbarium	Frodin, D.G. 8097	K000062297	ERR7619418
Rutaceae	*Vepris lanceolata*	Silica		K; K living collection no. 2010-201	ERR7620172
Rutaceae	*Zanthoxylum flavum*	DNA	Hamilton, M.A. 612	K000214339; K DNA bank no. 41216	ERR7618726
Rutaceae	*Zanthoxylum nitidum*	SRA			SRR10861972
Rutaceae	*Zanthoxylum ovalifolium*	Silica	Costion, C. 1644	CNS131383	SRR22164183

Rutaceae	*Zieria alata*	DNA	Crayn, D.M. DMC1169	CNS135627; ATH DNA no. L_H1	ERR7619975
Sapindaceae	*Acer campestre*	DNA	Fay 407	K DNA bank no. 8163	ERR5033234
Sapindaceae	*Acer palmatum*	SRA			SRR1980924
Sapindaceae	*Acer rubrum*	SRA			SRR11712223
Sapindaceae	*Aesculus chinensis*	SRA			SRR8073719
Sapindaceae	*Aesculus pavia*	SRA			ERR2040474
Sapindaceae	*Alatococcus siqueirae*	Herbarium	Folli 1761	K	ERR5034694
Sapindaceae	*Alectryon carinatum*	DNA	Munzinger 6743	K DNA bank no. 70369	ERR5033290
Sapindaceae	*Allophylastrum frutescens*	Herbarium	Lima 812	K	ERR5033461
Sapindaceae	*Allophylus* sp.	DNA	Buerki, S. 164	K; K DNA bank no. 66869	ERR5084249
Sapindaceae	*Amesiodendron chinense*	Herbarium	Buerki, S. 117	BM	ERR5033462
Sapindaceae	*Aporrhiza paniculata*	Herbarium	Swnowden 1669	BM	ERR5034719
Sapindaceae	*Arfeuillea arborescens*	DNA	Chase, M.W. 2122	K DNA bank no. 2013	ERR5033200
Sapindaceae	*Arytera litoralis*	DNA	Chase, M.W. 2123	K DNA bank no. 2014	ERR5034624
Sapindaceae	*Atalaya capensis*	DNA	Ohaeri, A.O s.n.	K DNA bank no. 40471	ERR5033277
Sapindaceae	*Athyana weinmanniifolia*	DNA	Pennington 17581	K DNA bank no. 18374	ERR5006146
Sapindaceae	*Beguea apetala*	DNA	Buerki, S. 149	G	ERR5033487
Sapindaceae	*Billia hippocastanum*	DNA	Pennington & Zamora 604	K DNA bank no. 1416	ERR5033194
Sapindaceae	*Blighia sapida*	DNA	Chase, M.W. 2124	K DNA bank no. 2015	ERR5033201
Sapindaceae	*Blighiopsis pseudostipularis*	Herbarium	Troupin 7717	BM	ERR5034695
Sapindaceae	*Blomia prisca*	Herbarium	Martinez *et al.* 3016	BM	ERR5033464
Sapindaceae	*Boniodendron parviflorum*	Herbarium	Ford 291	K000701344	ERR5034696
Sapindaceae	*Bridgesia incisifolia*	DNA	Killip & Pisano 39778	K DNA bank no. 2671	ERR5033219
Sapindaceae	*Camptolepis ramiflora*	Herbarium	Gautier, L. 4373	G	ERR5034697
Sapindaceae	*Castanospora alphandi*	Herbarium	Hartley & Hyland 15106	K	ERR5033465

Sapindaceae	*Cnesmocarpon dasyantha*	Herbarium	Forbes s.n.	BM	ERR5033466
Sapindaceae	*Conchopetalum brachysepalum*	Herbarium	Randriamampionona 637	MO	ERR5033467
Sapindaceae	*Cossinia australiana*	Silica	Pollock, A.B. ABP2852	BRI759067	SRR22164182
Sapindaceae	*Cubilia cubili*	DNA	Chase, M.W. 2125	K DNA bank no. 2016	ERR5033202
Sapindaceae	*Cupania rubiginosa*	DNA	Mori 8868	K DNA bank no. 3618	ERR5006129
Sapindaceae	*Cupaniopsis anacardioides*	DNA	Chase, M.W. 217	K DNA bank no. 2198	ERR5006157
Sapindaceae	*Cupaniopsis macropetala*	DNA	Deby 1227	K DNA bank no. 66952	ERR5033495
Sapindaceae	*Cupaniopsis trigonocarpa*	Herbarium	Veillon 720	L0469922	ERR5034720
Sapindaceae	*Deinbollia pinnata*	DNA	Odewo, T.K s.n.	K DNA bank no. 40408	ERR5033275
Sapindaceae	*Delavaya yunnanensis*	DNA	Forrest 20682	K DNA bank no. 3623	ERR5033222
Sapindaceae	*Diatenopteryx sorbifolia*	DNA	Tressens 3504	K DNA bank no. 267	ERR5006127
Sapindaceae	*Dictyoneura obtusa*	Herbarium	Polak 1233	BM	ERR5034030
Sapindaceae	*Dilodendron costaricense*	Herbarium	Galdames 5516	BM	ERR5033468
Sapindaceae	*Dimocarpus longan*	DNA	Buerki, S. 113	G	ERR5033486
Sapindaceae	*Diploglottis campbellii*	DNA	Chase, M.W. 2048	K DNA bank no. 1857	ERR5033195
Sapindaceae	*Diplopeltis huegelii*	DNA	Chase, M.W. 2192	K DNA bank no. 1907	ERR5033197
Sapindaceae	*Dipteronia dyeriana*	SRA			SRR2127991
Sapindaceae	*Dipteronia sinensis*	DNA	Chase, M.W. 19988	K DNA bank no. 19861, K living collection no. 1998-139	ERR5006151
Sapindaceae	*Dodonaea dodecandra*	DNA	Harrington, M. MH291	CNS141698; ATH DNA bank no. D1551	ERR7619976
Sapindaceae	*Dodonaea viscosa*	SRA			SRR1914335
Sapindaceae	*Elattostachys falcata*	Herbarium	Buerki 176	G	ERR5033287
Sapindaceae	*Eriocoelum rubiginosum*	DNA	Chase, M.W. 135	K DNA bank no. 743	ERR5084243
Sapindaceae	*Euchorium cubense*	Herbarium	Ekman 18678	K00058627	ERR5034698

Sapindaceae	*Euphorianthus longifolius*	DNA	Chase, M.W. 2126	K DNA bank no. 2017	ERR5034625
Sapindaceae	*Eurycorymbus cavaleriei*	DNA	Chase, M.W. 2127	K DNA bank no. 2018	ERR5033203
Sapindaceae	*Exothea paniculata*	Herbarium	Stafford 172	BM	ERR5033469
Sapindaceae	*Filicium decipiens*	DNA	Chase, M.W. 2128	K DNA bank no. 2019	ERR5033204
Sapindaceae	*Ganophyllum falcatum*	Herbarium	Chase 2129	K	ERR5033205
Sapindaceae	*Gereaua perrieri*	Herbarium	Rakotomalaza 165	MO	ERR5034699
Sapindaceae	*Glenniea africanus*	DNA	Adeyemi, T.O. 2	K DNA bank no. 40403	ERR5033274
Sapindaceae	*Gongrodiscus* sp.	DNA	Callmander, M.W. 896	G	ERR5084264
Sapindaceae	*Guindilia trinervis*	DNA	Chase, M.W. 802	K DNA bank no. 745; K living collection 1988-8205	ERR5033193
Sapindaceae	*Guioa rhoifolia*	DNA	Buerki, S. 16	K DNA bank no. 66859	ERR5034646
Sapindaceae	*Handeliodendron bodinieri*	Herbarium	Steward & Cheo 498	BM	ERR5033470
Sapindaceae	*Haplocoelopsis africana*	Herbarium	Dale 382	BM000073098	ERR5033471
Sapindaceae	*Haplocoelum inopleum*	Herbarium	Kassner 287	BM000073104	ERR5033472
Sapindaceae	*Harpullia rhyticarpa*	Silica	Stuart, S.A. 1571	CNS143233	SRR22164181
Sapindaceae	*Hippobromus pauciflorus*	Herbarium	Maurin, O. 1551	BNRH0002073	ERR5033500
Sapindaceae	*Hirania rosea*	Herbarium	Kuchar 17237	K	ERR5034700
Sapindaceae	*Hornea mauritiana*	Herbarium	Friedmann 3369	K	ERR5033473
Sapindaceae	*Hypelate trifoliata*	Herbarium	Visaya M.J. 94	K	ERR5033488
Sapindaceae	*Jagera serrata*	DNA	Chase, M.W. 2130	K DNA bank no. 2021	ERR5033206
Sapindaceae	*Koelreuteria paniculata*	DNA	Chase, M.W. 115	K DNA bank no. 21878	ERR5033251
Sapindaceae	*Laccodiscus ferrugineus*	Herbarium	Talbot 415	K	ERR5033482
Sapindaceae	*Lecaniodiscus cupanioides*	DNA	Ameka *et al.* 221	K DNA bank no. 40835	ERR5033281
Sapindaceae	*Lepiderema ixiocarpa*	Herbarium	Jensen 883	L078480	ERR5034723
Sapindaceae	*Lepiderema punctulata*	Herbarium	Forster, P.I. 29953	L0784798	ERR5033492
Sapindaceae	*Lepidocupania arcuata*	DNA	Munzinger 6728	NOU; K DNA bank no. 70366	ERR5034728

Sapindaceae	*Lepidocupania lepidota*	DNA	Munzinger 6705	MPU; K DNA bank no. 70361	ERR5034727
Sapindaceae	*Lepidocupania squamosa*	Herbarium	MacKee 20428	L0469876	ERR5034721
Sapindaceae	*Lepidopetalum* sp.	DNA	Munzinger 6926	MO; K DNA bank no. 66895	ERR5084250
Sapindaceae	*Lepisanthes ramiflora*	DNA	Buerki, S. 324	K DNA bank no. 66876	ERR5034647
Sapindaceae	*Litchi chinensis*	DNA	Chase, M.W. 2131	K DNA bank no. 2022	ERR5033207
Sapindaceae	*Llagunoa nitida*	DNA	Penningron *et al.* 17552	K DNA bank no. 18373	ERR5006145
Sapindaceae	*Lophostigma plumosum*	Herbarium	Brooke 506	BM	ERR5033474
Sapindaceae	*Loxodiscus coriaceus*	DNA	Bradford 1136	K DNA bank no. 21333	ERR5006155
Sapindaceae	*Macphersonia cauliflora*	Herbarium	Rakotovao 3607	MO	ERR5034702
Sapindaceae	*Magonia pubescens*	DNA	Mori 16966	K DNA bank no. 3621	ERR5006131
Sapindaceae	*Majidea zanguebarica*	DNA	Verdcourt 2128	K DNA bank no. 362	ERR5006130
Sapindaceae	*Matayba tenax*	DNA	Chase, M.W. 2132	K DNA bank no. 2023	ERR5084244
Sapindaceae	*Melicoccus bijugatus*	DNA	Ogu	FHI; K DNA bank no. 40422	ERR5033276
Sapindaceae	*Mischarytera lautereriana*	Herbarium	Gray 485	K	ERR5033475
Sapindaceae	*Mischocarpus pyriformis*	DNA	Chase, M.W. 2059	K DNA bank no. 1868	ERR5033196
Sapindaceae	*Molinaea sessilifolia*	Herbarium	Ravelonarivo 1784	MO	ERR5034711
Sapindaceae	*Nephelium lappaceum*	SRA			SRR11613036
Sapindaceae	*Otonephelium stipulaceum*	Herbarium	Hassan Flora Project 2848	K	ERR5034703
Sapindaceae	*Pancovia bijuga*	DNA	Juillick s.n.	K DNA bank no. 40423	ERR5034644
Sapindaceae	*Pappea capensis*	DNA	Chase, M.W. 6585	K DNA bank no. 4852	ERR5033227
Sapindaceae	*Paranephelium xestophyllum*	DNA	Buerki, S. 345	G	ERR5033294
Sapindaceae	*Paullinia pinnata*	DNA	Adeyemi, T.O. 26	K DNA bank no. 40481	ERR5033278
Sapindaceae	*Pavieasia anamensis*	Herbarium	Cuong 415	L0405051	ERR5033494
Sapindaceae	*Pentascyphus thyrsiflorus*	Herbarium	Nee 42461	K	ERR5034704

Sapindaceae	*Placodiscus turbinatus*	Herbarium	s.n. 2239	K000426281	ERR5034715
Sapindaceae	*Plagioscyphus jumellei*	DNA	Buerki, S. 146	G	ERR5034713
Sapindaceae	*Podonephelium homei*	DNA	Callmander, M.W. 908	K DNA bank no. 41039	ERR5034645
Sapindaceae	*Pometia pinnata*	Herbarium	Buerki, S. 351	G	ERR5033484
Sapindaceae	*Pseudopteris decipiens*	DNA	Kakazomannjary 12529	K DNA bank no. 3617	ERR5006128
Sapindaceae	*Radlkofera calodendron*	DNA	Adeyemi, T.O. 3	K DNA bank no. 40496	ERR5033280
Sapindaceae	*Rhysotoechia flavescens*	Herbarium	Gray 4521	K	ERR5034706
Sapindaceae	*Rhysotoechia robertsonii*	Herbarium	Gray 5134	K	ERR5033476
Sapindaceae	*Sapindus mukorossi*	SRA			SRR8302229
Sapindaceae	*Sapindus saponaria*	DNA	Chase, M.W. 2136	K; K DNA bank no. 2027	ERR5033208
Sapindaceae	*Sarcopteryx squamosa*	Herbarium	Brass 13801	BM	ERR5034712
Sapindaceae	*Sarcotoechia cuneata*	Herbarium	Gray 2798	K	ERR5033477
Sapindaceae	*Serjania communis*	DNA	Chase, M.W. 2138	K DNA bank no. 2029	ERR5033209
Sapindaceae	*Serjania incana*	Herbarium	Arbo 2635	K	ERR5034701
Sapindaceae	*Sisyrolepis muricata*	Herbarium	Kerr 2187	K	ERR5034707
Sapindaceae	*Stadmannia oppositifolia*	DNA	Page, W. 41	K DNA bank no. 30007	ERR5034636
Sapindaceae	*Stocksia brahuica*	Herbarium	Popov 231	BM	ERR5034708
Sapindaceae	*Storthocalyx chryseus*	DNA	Callmander, M.W. 918	G	ERR5033485
Sapindaceae	*Synima cordieri*	DNA	Gray, B. 9624	K DNA bank no. 70328	ERR5033289
Sapindaceae	*Talisia nervosa*	DNA	Pennington, T 628	K DNA bank no. 212	ERR5033212
Sapindaceae	*Thinouia myriantha*	Herbarium	Philipson 2197	BM	ERR5033478
Sapindaceae	*Thouinia acuminata*	DNA	Liston 6332	K DNA bank no. 3185	ERR5033221
Sapindaceae	*Thouinidium decandrum*	Herbarium	Monro 196	BM00056489	ERR5033479
Sapindaceae	*Tina tamatavensis*	DNA	Buerki, S. 14	G	ERR5034709
Sapindaceae	*Toechima erythrocarpum*	Herbarium	Gray, B. 9084	K	ERR5033483
Sapindaceae	*Toulicia reticulata*	Herbarium	Pennington *et al.* 16988	K	ERR5034714
Sapindaceae	*Trigonachras postardanjeisin*	DNA	Buerki, S. 338	K DNA bank no. 66883	ERR5034648

Sapindaceae	*Tripterodendron filicifolium*	Herbarium	Glaziou 11822	P	ERR5034710
Sapindaceae	*Tristira triptera*	DNA	Chase, M.W. 2139	K DNA bank no. 203	ERR5033210
Sapindaceae	*Tristiropsis acutangula*	Silica	Hyland, B. 16432	QRS122503	SRR22164180
Sapindaceae	*Tsingya bemarana*	Herbarium	Leandri 1969	K	ERR5034716
Sapindaceae	*Ungnadia speciosa*	DNA	Chase, M.W. 2854	K DNA bank no. 2661; K living collection no. 1996-15	ERR5033218
Sapindaceae	*Urvillea ulmacea*	Herbarium	Rolando Tn Ohiz 166	BM	ERR5033480
Sapindaceae	*Vouarana guianensis*	Herbarium	Nascimento 624	BM	ERR5033481
Sapindaceae	*Xanthoceras sorbifolium*	DNA	Maurin, O. 4384	K DNA bank no. 74254	ERR5033596
Sapindaceae	*Xerospermum noronhianum*	DNA	Chase, M.W. 214	K DNA bank no. 2031	ERR5033211
Sapindaceae	*Zanha golungensis*	DNA	Adeyemi, T.O. 35	K DNA bank no. 40482	ERR5033279
Sapindaceae	*Zollingeria borneensis*	Herbarium	Elmer 21128	BM	ERR5034726
Sapotaceae	*Manilkara zapota*	SRA			ERR2040506
Sarcolaenaceae	*Leptolaena cuspidata*	SRA			ERR4180027
Setchellanthaceae	*Setchellanthus caeruleus*	SRA			ERR4180002
Simaroubaceae	*Ailanthus altissima*	SRA			ERR2040477
Simaroubaceae	*Ailanthus triphysa*	Silica	Ford, A. 3095	QRS126785	ERR7619988
Simaroubaceae	*Amaroria soulameoides*	Herbarium	Prasad *et al.* EY055	K001275555	ERR7620398
Simaroubaceae	*Castela coccinea*	Silica	Bourdy, G. GB1967	SPF	ERR7620421
Simaroubaceae	*Castela emoryi*	Herbarium	Way *et al.* WSSB14	K001275556	ERR7620399
Simaroubaceae	*Eurycoma longifolia*	DNA	RS.Th. 7.0218	K DNA bank no. 20645	ERR7620196
Simaroubaceae	*Gymnostemon zaizou*	Herbarium	Bamps 2593	K00127556	ERR7620404
Simaroubaceae	*Hannoa klaineana*	Herbarium	van der Burgt 2058	K001243682	ERR7620401
Simaroubaceae	*Homalolepis glabra*	Silica	Devecchi, M.F. MFD298	SPF	ERR7620422
Simaroubaceae	*Nothospondias staudtii*	Herbarium	Daramola 24	K00004090	ERR7620400
Simaroubaceae	*Odyendea gabunensis*	Herbarium	Cheek *et al.* 15832	K000673044	ERR9229991
Simaroubaceae	*Perriera madagascariensis*	Herbarium	Phillipson *et al.* 6271	K001275558	ERR7620402
Simaroubaceae	*Picrasma crenata*	Silica	Alves, G.G.N. 76	SPF	ERR7620419

Simaroubaceae	*Pierreodendron africanum*	Herbarium	Ern 2342	K001275559	ERR7620403
Simaroubaceae	*Quassia amara*	DNA	Chase, M.W. 34485	K DNA bank no. 3494	ERR7620213
Simaroubaceae	*Samadera* sp. *Tozer Range*	Silica	Baba, Y. 579	CNS135404	ERR7619985
Simaroubaceae	*Simaba orinocensis*	Silica	Devecchi, M.F. MFD361	SPF	ERR7620420
Simaroubaceae	*Simarouba glauca*	DNA	Chase, M.W. 124	K DNA bank no. 21887	ERR7620144
Stachyuraceae	*Stachyurus praecox*	SRA			ERR2040307
Staphyleaceae	*Staphylea trifolia*	SRA			ERR2040308
