# Supplementary material for: Phylogenomic analyses of Sapindales support new family relationships, rapid Mid-Cretaceous Hothouse diversification, and heterogeneous histories of gene duplication
Source: Front Plant Sci. 2023 Mar 7;14:1063174. doi: 10.3389/fpls.2023.1063174 (PMC10028101; doi:10.3389/fpls.2023.1063174)
Supplement: Supplementary Material 1 — List of ingroup and outgroup samples included in the genus-level phylogenetic analysis of Sapindales and their NCBI SRA accession numbers. [file DataSheet_1.zip › Supplementary Material/Supplementary Material 3.pdf]

Supplementary file 3 Profiles of each fossil used for time-calibration of the Sapindales tree.

**Calibration no.**	C02
**Family**	Sapindaceae
**Full taxon name**	†*Sapindospermum nitidum* Knobloch & Mai
**Reference (first description)**	Knobloch & Mai (1986)
**Reference (latest description)**	
**Organs**	Seed
**Specimens**	Holotype: UUG EK 39618
**Locality**	Trebec Tj-4a, 76-77 m, graue Schluffe, Klikov-Schichtenfolge
**Formation**	
**Country**	Czech Republic
**Reference (age)**	Knobloch & Mai (1986)
**Oldest stratum**	Turonian
**Age quality**	Unrevised (old source < 2000)
**Age justification**	Paleocarpological evidence places the Klikov layer sequence in the Oberturon to Santon.
**Reference (fossil relationships)**	Knobloch & Mai (1986)
**Node assignment method**	Intuitive or unspecified (trusted source)
**Reconciliation method**	Pre-molecular era (before 1990)
**Min. age (Mya)**	89.8
**Calibration**	crown Sapindales
**Node justification**	This seed is thought to be attributable to Sapindoideae (Sapindaceae), and was placed in the extinct genus *Sapindospermum*. Other *Sapindospermum* seeds are known from the Cretaceous of North America, Greenland, Europe, and Siberia, and Sapindaceae fossil wood (*Sapindoxylum*) has been recovered from Cretaceous beds in Egypt (Knobloch and Mai 1986). However, given the age of the fossil and inability to assign it to an extant taxon, we use this fossil conservatively to calibrate the minimum age for the crown of Sapindales.
**Calibration no.**	C03
**Family**	Sapindaceae
**Full taxon name**	†*Aesculus hickeyi* Manchester
**Reference (first description)**	Manchester (2001)
**Reference (latest description)**	
**Organs**	Leaf, fruit, seed
**Specimens**	Holotype: UF 30618
**Locality**	Farmers Butte, North Dakota (UF locality 18744)
**Formation**	Fort Union Formation
**Country**	USA
**Reference (age)**	Belt (2004)
**Oldest stratum**	Paleocene
**Age quality**	Revised (stratigraphic)

**Age justification**	Stratigraphy
**Reference (fossil relationships)**	Magallón *et al.* (2015)
**Node assignment method**	Intuitive or unspecified (trusted source)
**Reconciliation method**	Molecular tree only
**Min. age (Mya)**	56
**Calibration**	crown Sapindaceae
**Node justification**	The leaves of *Aesculus hickeyi* were previously assigned to *Carya antiquorum* (Juglandaceae; Hickey, 1977) and initially known from isolated leaflets in the Golden Valley Formation and Farmers Butte locality. Manchester (2001) was the first to recognise the affinity of the leaves with *Aesculus* (Sapindaceae). We accept the assignment by Manchester (2001) and use this fossil taxon to calibrate the crown node of Sapindaceae.
**Calibration no.**	C04
**Family**	Sapindaceae
**Full taxon name**	†*Dipteronia brownii* McClain & Manchester
**Reference (first description)**	McClain & Manchester (2001)
**Reference (latest description)**	
**Organs**	Fruit
**Specimens**	Holotype: UWBM 39729
**Locality**	Hell's Half Acre, Wyoming
**Formation**	Fort Union Formation
**Country**	USA
**Reference (age)**	Belt (2004)
**Oldest stratum**	Middle Paleocene
**Age quality**	Revised (stratigraphic)
**Age justification**	Based on palynological evidence, the *Dipteronia* fruits were found in a pollen zone P-4 that is considered to be middle Paleocene (60–63 Mya)."
**Reference (fossil relationships)**	McClain & Manchester (2001)
**Node assignment method**	Intuitive or unspecified (trusted source)
**Reconciliation method**	Molecular tree only
**Min. age (Mya)**	60
**Calibration**	stem *Dipteronia*
**Node justification**	The venation is the same in the fossils as in modern *Dipteronia*, complete with fine reticulation on the wing. The fossil *Dipteronia* fruits were assigned to *D. brownii* sp. nov., and most closely resemble extant species *D. sinensis* (McClain & Manchester, 2001). We accept the assignment of this fossil to *Dipteronia*, however do not know when in the evolution of the genus this species evolved. Therefore we take the conservative approach of using this fossil to calibrate the stem node of *Dipteronia*.
**Calibration no.**	C05
**Family**	Sapindaceae
**Full taxon name**	†*Acer* sp.
**Reference (first description)**	Crane *et al*. (1990)

**Reference (latest description)**	
**Organs**	Leaf
**Specimens**	
**Locality**	
**Formation**	
**Country**	
**Reference (age)**	
**Oldest stratum**	Late Paleocene
**Age quality**	Unrevised (old source < 2000)
**Age justification**	
**Reference (fossil relationships)**	Magallón & Castillo (2009)
**Node assignment method**	Intuitive or unspecified (trusted source)
**Reconciliation method**	Molecular tree only
**Min. age (Mya)**	56
**Calibration**	stem *Acer*
**Node justification**	*Acer* fruits first occur in the late Paleocene of North America, and are well represented in the Eocene and later Tertiary of North America, Europe and Asia (Manchester 1999). We accept the assignment of this fossil to *Acer* and take the conservative approach of using it to constrain the stem node of *Acer* to a minimum age of 56 Mya.
**Calibration no.**	C06
**Family**	Sapindaceae
**Full taxon name**	†*Koelreuteria allenii* (Lesq.) W. N. Edwards
**Reference (first description)**	Wang et al (2013)
**Reference (latest description)**	
**Organs**	Fruit
**Specimens**	Holotype: USNM P1617
**Locality**	Wyoming
**Formation**	Green River Formation
**Country**	USA
**Reference (age)**	Smith *et al*. (2003)
**Oldest stratum**	Eocene
**Age quality**	Revised (radioisotropic)
**Age justification**	Radioisotropic evidence (Smith *et al*. 2003).
**Reference (fossil relationships)**	Wang *et al*. (2013)
**Node assignment method**	Intuitive or unspecified (trusted source)
**Reconciliation method**	Molecular tree only
**Min. age (Mya)**	52
**Calibration**	stem *Koelreuteria*
**Node justification**	This is the oldest known specimen of a capsular valve of *Koelreuteria* Wang *et al.* (2013). We accept the assignment of the fossil to *Koelreuteria* and use it conservatively to constrain the stem node of the genus to a minimum age of 52 Mya.

**Calibration no.**	C07
**Family**	Sapindaceae
**Full taxon name**	†*Allophylus graciliformis* (Berry) Berry
**Reference (first description)**	Panti (2018)
**Reference (latest description)**	
**Organs**	Leaf
**Specimens**	MPM PB 3079–3088
**Locality**	Santa Cruz Province, Patagonia
**Formation**	Río Turbio Formation
**Country**	Argentina
**Reference (age)**	Panti (2018)
**Oldest stratum**	Early Eocene
**Age quality**	Revised (stratigraphic)
**Age justification**	The unit was divided into upper and lower informal members. Evidence for the age of the formation comes mainly from dinoflagellates and detrital zircon U/Pb. Dinoflagellate data suggests the age of Early/Middle Eocene for the lower member and Middle/Late Eocene for the upper member. Detrital zircon U/Pb evidence restricts the lower member to the Early Eocene and indicate an Oligocene age for the upper member. Specimens of this fossil were found in the upper and lower members, and so the minimum age of the lower member is used.
**Reference (fossil relationships)**	
**Node assignment method**	Intuitive or unspecified (trusted source)
**Reconciliation method**	Molecular tree only
**Min. age (Mya)**	46
**Calibration**	stem *Allophylus*
**Node justification**	These fossils match with *Allophylus graciliformis* (Berry) as described by Hünicken (1967), particularly with respect to the shape of the base and apex.This species is thought to be related to the extant *Allophylus edulis* (A. St.– Hil., A. Juss. & Cambess.) Hieron. ex Niederl. of northern Argentina (Panti, 2018). We therefore accept the assignment of this fossil to *Allophylus*, and use it conservatively to constrain the stem node of the genus.
**Calibration no.**	C08
**Family**	Burseraceae
**Full taxon name**	†*Bursera inaequalateralis* (Lesq.) MacGintie
**Reference (first description)**	MacGinitie (1969)
**Reference (latest description)**	
**Organs**	Leaf
**Specimens**	Hypotypes: PA20624, PA20722
**Locality**	Locality PA106, Wardell Ranch, Utah
**Formation**	Green River Flora
**Country**	USA
**Reference (age)**	Smith *et al*. (2003)
**Oldest stratum**	Eocene
**Age quality**	Revised (radioisotropic)

**Age justification**	The Green River Formation is thought to encompass a ~5 My period between ca. 53.5 and 48.5 Ma, based on the global marine O isotope record.
**Reference (fossil relationships)**	MacGinitie (1969)
**Node assignment method**	Intuitive or unspecified (trusted source)
**Reconciliation method**	Pre-molecular era (before 1990)
**Min. age (Mya)**	48.5
**Calibration**	crown Burserinae
**Node justification**	*Bursera inaequalateralis* (Lesq.) MacGinitie is similar to several extant *Bursera* species (MacGinitie, 1969), namely *B. penicillata* in *B.* subg. *Elaphrium* and *B. tomentosa*. We therefore assign this fossil conservatively to the crown of Burserinae.
**Calibration no.**	C09
**Family**	Burseraceae
**Full taxon name**	†*Bursericarpum aldwickense* Chandler
**Reference (first description)**	Chandler (1961)
**Reference (latest description)**	
**Organs**	Pyrene, seed
**Specimens**	Holotype: v.30054
**Locality**	Upper Fish Tooth Bed, Bognor, Sussex
**Formation**	London Clay Flora
**Country**	UK
**Reference (age)**	Chandler (1961)
**Oldest stratum**	Ypresian
**Age quality**	Unrevised (old source < 2000)
**Age justification**	Chandler (1961) places the London Clay Flora in the Ypresian, an age that has apparently not been challenged since.
**Reference (fossil relationships)**	Magallón *et al.* (2015)
**Node assignment method**	Intuitive or unspecified (trusted source)
**Reconciliation method**	Molecular tree only
**Min. age (Mya)**	47.8
**Calibration**	stem Protium alliance
**Node justification**	*Bursericarpum aldwickense* is thought to be a relative of the Protium alliance based on the number of pyrenes per fruit (Weeks et al., 2005; Daly et al., 2011). We therefore use this fossil to calibrate the stem of the Protium alliance of Burseraceae.
**Calibration no.**	C10
**Family**	Anacardiaceae
**Full taxon name**	†*Coahuiloxylon terrazasiae* Estrada-Ruiz, Martínez-Vabrera & Cevallos-Ferriz
**Reference (first description)**	Estrada-Ruiz *et al.* (2010)
**Reference (latest description)**	
**Organs**	Wood
**Specimens**	Holotype: IGM-PB 1295, LPB 4679-4693
**Locality**	Atascoso Ranch, 7 km N of Melchor Mu´zquiz town, Mu´zquiz Municipality, Coahuila

**Formation**	Olmos Formation
**Country**	Mexico
**Reference (age)**	Estrada-Ruiz *et al*. (2010)
**Oldest stratum**	Campanian
**Age quality**	Revised (stratigraphic)
**Age justification**	The Olmos Formation has been placed in the early Maastrichtian based on foraminifers, dinoflagellates, acritarchs, bivalve fauna and pollen. Ammonite evidence suggests places the formation in the late Campanian age based on ammonites. Therefore, we use late Campanian-early Maastrichtian for this calibration.
**Reference (fossil relationships)**	Estrada-Ruiz *et al*. (2010)
**Node assignment method**	Intuitive or unspecified (trusted source)
**Reconciliation method**	Molecular tree only
**Min. age (Mya)**	72.1
**Calibration**	stem Anacardiaceae
**Node justification**	The wood structure of *Coahuiloxylon terrazasiae* resembles that of Anacardiaceae and Burseraceae based on the presence of indistinct growth rings, septate and/or nonseptate fibers, alternate intervessel pits and vessel-ray parenchyma pits with reduced borders, simple perforation plates, scanty paratracheal, vasicentric and apotracheal diffuse axial parenchyma, and heterocellular rays (Estrada-Ruiz *et al*., 2010). This is also supported by molecular and anatomical analyses (Terrazas 1994). There is no single wood anatomical character separating these two families; however, the combination of type IIA rays and absence of radial canals place the fossil in *Coahuiloxylon* or *Mauria* in Anacardiaceae (Terrazas, 1994; Estrada-Ruiz et al., 2010). *Coahuiloxylon* and *Mauria* share other diagnostic features of Anacardiaceae such as presence of both nonseptate and septate fibers, paratracheal and apotracheal parenchyma, and heterogeneous type IIA rays (Estrada-Ruiz *et al*., 2010). We therefore use this fossil conservatively to calibrate the stem node of Anacardiaceae to a minimum age of 72.1 Mya.

**Calibration no.**	C11
**Family**	Anacardiaceae
**Full taxon name**	†*Choerospondias sheppeyensis* (Reid & Chandler) Chandler
**Reference (first description)**	Reid & Chandler (1933)
**Reference (latest description)**	Chandler (1961)
**Organs**	Fruit, seed
**Specimens**	Holotype: V.22554
**Locality**	Island of Sheppey
**Formation**	London Clay Flora
**Country**	UK
**Reference (age)**	Chandler (1961)
**Oldest stratum**	Ypresian
**Age quality**	Unrevised (old source < 2000)
**Age justification**	Chandler (1961) places the London Clay Flora in the Ypresian, an age that has apparently not been challenged since.
**Reference (fossil relationships)**	Chandler (1961)
**Node assignment method**	Intuitive or unspecified (trusted source)
**Reconciliation method**	Pre-molecular era (before 1990)

**Min. age (Mya)**	47.8
**Calibration**	crown Anacardiaceae
**Node justification**	These fruits most resemble *Sclerocarya* and *Choerospondias*, but can be separated from extant *Sclerocarya* on the higher number of locules and the absence of woody plugs in their apices (Reid & Chandler, 1933). *Choerospondias* has a similar number of locules but a larger endocarp, and the longitudinal rows of holes on the lateral surfaces between the locules in extant species are fewer and more irregular in the fossils. The fossils are thought to be most closely realted to the extant *C. axillaris* (Roxb.) (Chandler 1961). However, due to the similarity to *Sclerocarya* and broad age range for the Flora we will use this fossil conservatively to date the crown node of Anacardiaceae to a minimum of 47.8 Mya.

**Calibration no.**	C12
**Family**	Anacardiaceae
**Full taxon name**	†*Dracontomelon macdonaldii* (Berry) Herrera, Manchester & Jaramillo
**Reference (first description)**	Herrera *et al*. (2012)
**Reference (latest description)**	
**Organs**	Fruit, seed
**Specimens**	Lectotype: USNM 35316b
**Locality**	Tonosí River, Punta Búcaro, Los Santos State, Azuero Peninsula
**Formation**	Búcaro Formation
**Country**	Panama
**Reference (age)**	Herrera *et al*. (2012)
**Oldest stratum**	Late Eocene
**Age quality**	Revised (stratigraphic)
**Age justification**	Mollusc and foraminifera evidence variously suggest an early to late Eocene age for this sequence.
**Reference (fossil relationships)**	Herrera *et al*. (2012)
**Node assignment method**	Apomorphy-based (apomorphies unlisted or tested)
**Reconciliation method**	Molecular tree only
**Min. age (Mya)**	33.9
**Calibration**	crown Spondioideae (-*Campnosperma*)
**Node justification**	The fossil endocarps resemble most genera of the Spondioideae subfamily of Anacardiaceae, and particularly the extant genus *Dracontomelon* (Herrera *et al*. 2012). The combination of the presence of endocarps with five single-seeded locules, radially-arranged convex-dorsal-apical germination valves without median longitudinal slits, five lacunae, ten peripheral equatorial apertures connected to lacunae, and the tissue organisation of the endocarp is unique to *Dracontomelon* (Herrera *et al*. 2012). However, due to the presence of characters also featuring in other Spondioid genera we use this fossil conservatively to calibrate the crown of Spondioideae, excluding *Campnosperma*.

**Calibration no.**	C13
**Family**	Anacardiaceae
**Full taxon name**	†*Spondias rothwellii* Herrera, Carvalho, Jaramillo & Manchester sp. nov.
**Reference (first description)**	Herrera *et al*. (2019)
**Reference (latest description)**	
**Organs**	Fruit, seed

**Specimens**	Holotype: UF60773. Other specimens: UF60774, UF60775, UF60776, UF60784, UF60777, UF60778–UF60783.
**Locality**	Gaillard Cut section (Lirio East outcrop) of the southeastern part of the Panama Canal
**Formation**	Lower Cucaracha Formation
**Country**	Panama
**Reference (age)**	Herrera *et al*. (2019)
**Oldest stratum**	Early Miocene
**Age quality**	Revised (radioisotropic)
**Age justification**	The age of this formation has been deduced from mammals, pollen, marine invertebrates, magnetostratigraphy, and radiometric dating evidence, all of which indicate an age of ca. 19– 18.5 Ma for the Cucaracha Formation.
**Reference (fossil relationships)**	Herrera *et al*. (2019)
**Node assignment method**	Intuitive or unspecified (trusted source)
**Reconciliation method**	Molecular tree only
**Min. age (Mya)**	18.5
**Calibration**	stem *Spondias*
**Node justification**	Fruits of *S. rothwellii* are thought to be indistinguishable from some extant species of *Spondias* based on endocarp features - particularly the deer antler–like outline of the in transverse section and ventral rows of orbicules within the lacunae that align the fossils with extant Neotropical species (Herrera *et al*. 2019). Hence, the fossil is most similar morphologically to the extant New World representatives of *Spondias*. However, we use this fossil conservatively to calibrate the stem of *Spondias* to a minimum age of 18.5 Mya.

**Calibration no.**	C14
**Family**	Anacardiaceae
**Full taxon name**	†*Anacardium germanicum* Manchester *et al.*
**Reference (first description)**	Manchester *et al*. (2007)
**Reference (latest description)**	
**Organs**	Fruit
**Specimens**	Holotype: SM.B Me 7139. Paratypes: SM.BMe1770,2034,7156,18095.
**Locality**	Messel Pit, Messel oil shale, Messel
**Formation**	Messel Formation
**Country**	Germany
**Reference (age)**	Manchester *et al*. (2007)
**Oldest stratum**	Middle Eocene (Lutetian assumed)
**Age quality**	Revised (stratigraphic)
**Age justification**	Radiometric dating of the underlying volcaniclastic material suggests a Middle Eocene age for the Messel Formation.
**Reference (fossil relationships)**	Manchester *et al*. (2007)
**Node assignment method**	Apomorphy-based (apomorphies unlisted or tested)
**Reconciliation method**	Molecular tree only
**Min. age (Mya)**	41.2
**Calibration**	stem *Fegimanra* + *Anacardium*

**Node justification**	The hypocarp is a specialised structure known only in from Anacardiaceae and the presence of a hypocarp in the fossil specimens aligns the fossil to *Semecarpus*, *Fegimanra* and *Anacardium* (Manchester *et al*. 2007). Based on the shape and surfaces of the drupe and the attachment position of the pedicel, the assignment of the fossil was narrowed to either *Fegimanra* or *Anacardium*; based on the shape of the hypocarp it was decided that this fossil belongs to *Anacardium* (Manchester *et al*. 2007). Although we accept the assignment of this fossil to *Anacardium*, based on the shared characters with *Fegimanra*, we take a conservative approach and use this fossil to calibrate the stem of *Anacardium* and *Fegimanra*.
**Calibration no.**	C15
**Family**	Anacardiaceae
**Full taxon name**	†*Mangifera paleoindica* Sawangchote, Grote, and Dilcher
**Reference (first description)**	Sawangchote *et al.* (2009)
**Reference (latest description)**	
**Organs**	Leaf
**Specimens**	Holotype: SUT083
**Locality**	Ban Pa Kha mine, Li Basin, Li District, Lamphun Province
**Formation**	Ban Pa Kha Subbasin
**Country**	Thailand
**Reference (age)**	Sawangchote *et al.* (2009)
**Oldest stratum**	Late Oligocene
**Age quality**	Revised (stratigraphic)
**Age justification**	The age determination of the Ban Pa Kha Subbasin is uncertain and ranges from Paleogene to early Miocene, on the basis of leaf fossils and pollen. However, the leaf evidence has been discredited and pollen evidence is considered to be more reliable. Therefore age determined by pollen evidence of Oligocene to early Miocene is taken.
**Reference (fossil relationships)**	Sawangchote *et al.* (2009)
**Node assignment method**	Apomorphy-based (apomorphies unlisted or tested)
**Reconciliation method**	Molecular tree only
**Min. age (Mya)**	23.03
**Calibration**	stem *Mangifera*
**Node justification**	Leaf architecture was studied in detail for multiple genera in Anacardiaceae and the authors are confident that the specimens belong to the modern genus *Mangifera*. Comparison of leaf architecture to many extant *Mangifera* species indicates this species is most similar to extant species *Mangifera indica* (Sawangchote *et al.* 2009). We accept the assignment of this fossil to *Mangifera*, however take a conservative approach to dating and use this fossil to calibrate the stem of *Mangifera*.
**Calibration no.**	C16
**Family**	Anacardiaceae
**Full taxon name**	†*Cotinus fraterna* (Lesquereux) MacGinitie
**Reference (first description)**	MacGinitie (1953)
**Reference (latest description)**	Manchester (2001)
**Organs**	Leaf
**Specimens**	Syntypes: USNM 405894, USNM 405895
**Locality**	Florissant, Colorado
**Formation**	Florissant Formation

**Country**	USA
**Reference (age)**	Manchester (2001)
**Oldest stratum**	Late Eocene
**Age quality**	Revised (radioisotropic)
**Age justification**	Most recently, potassium Argon dating dates the Florissant Formation at 35.0 ± 0.8 Ma, and 40Ar/39Ar dating places the formation at 34.07 ± 0.10 Ma. As such, the Florissant beds could either be late Eocene or early Oligocene.
**Reference (fossil relationships)**	MacGinitie (1953)
**Node assignment method**	Intuitive or unspecified (trusted source)
**Reconciliation method**	Pre-molecular era (before 1990)
**Min. age (Mya)**	33.9
**Calibration**	stem *Cotinus*
**Node justification**	Assignment to *Cotinus fraterna* by MacGinitie (1953) does not seem to have been disputed since its publication and revision by Manchester; we therefore accept its placement in *Cotinus* and use this fossil to calibrate the stem node of the genus.

**Calibration no.**	C17
**Family**	Anacardiaceae
**Full taxon name**	*†Pistacia* sp.
**Reference (first description)**	Grímsson *et al*. (2020)
**Reference (latest description)**	
**Organs**	Pollen
**Specimens**	NA
**Locality**	Lavanttal Basin
**Formation**	Lavanttal Basin
**Country**	Austria
**Reference (age)**	Grímsson *et al*. (2011)
**Oldest stratum**	Middle Miocene (middle to upper Serravallian)
**Age quality**	Unrevised (recent source)
**Age justification**	The stratigraphical position within the sequence, the thickness of the sediments surrounding the coals seems, estimated accumulation rates, and hiatus events above the seams suggests the basin is most likely between 12.6 and 12.5 Ma.
**Reference (fossil relationships)**	Grímsson *et al*. (2020)
**Node assignment method**	Apomorphy-based (apomorphies unlisted or tested)
**Reconciliation method**	Molecular tree only
**Min. age (Mya)**	12.7
**Calibration**	stem Pistacia
**Node justification**	*Pistacia* is characterised by unique pollen that has been described in detail (Grímsson *et al*., 2020). The apertures of the fossil pollen from Lavanttal correspond to the colpi of *P. terebinthus* L. as described by Halbritter and Weis (2016). Many debatable *Pistacia* fossils have been identified up to the Cretaceous (Edwards and Wonnacott 1935; Grímsson *et al.* 2020). Based on the unique and well-described morphology of *Pistacia* pollen and potential presence of *Pistacia* pollen in the Cretaceous fossil record, we think it is reasonable to apply a middle Miocene age as a minimum constraint on the stem node of *Pistacia*.

**Calibration no.**	C18
**Family**	Anacardiaceae
**Full taxon name**	†*Loxopterygium laplayense* Burnham and Carranco
**Reference (first description)**	Burnham & Carranco (2004)
**Reference (latest description)**	
**Organs**	Fruit
**Specimens**	Holotype: EPN 1054
**Locality**	RJB 9602 (‘‘Azogues View’’), Cuenca Basin
**Formation**	Azogues Formation
**Country**	Ecuador
**Reference (age)**	Hungerbühler *et al*. (2002)
**Oldest stratum**	Middle Miocene
**Age quality**	Unrevised (recent source)
**Age justification**	Deposits of the Azogues or Guapan Members of the Azogues Formation have been dated at 13.0–10.2 Ma.
**Reference (fossil relationships)**	Burnham & Carranco (2004)
**Node assignment method**	Apomorphy-based (apomorphies unlisted or tested)
**Reconciliation method**	Molecular tree only
**Min. age (Mya)**	13.0
**Calibration**	stem *Loxopterygium*
**Node justification**	This fossil is distinctive in its medially placed stigma remnant on the backbone of the wing, its small size, the remnant calyx at the proximal end of the locule, and the frequently erose to serrate wing tip. This combination of characters assigns the specimens in the genus Loxopterygium of the Anacardiaceae, however it cannot be assigned to any of the extant species of *Loxopterygium* (Burnham & Carranco 2004). We therefore use this fossil to constrain the stem node of *Loxopterygium.*
**Calibration no.**	C19
**Family**	Simaroubaceae
**Full taxon name**	†*Ailanthus confucii* Unger
**Reference (first description)**	Collinson *et al.* (2012)
**Reference (latest description)**	
**Organs**	Fruit
**Specimens**	Specimens: SM.B Me 4006, 4232, 4233, 4747, 4785, 4786, 16837, 21808, 23395, 24010.
**Locality**	Messel Pit, Messel oil shale, Messel
**Formation**	Messel Formation
**Country**	Germany
**Reference (age)**	Collinson *et al.* (2012)
**Oldest stratum**	Middle Eocene (Lutetian assumed)
**Age quality**	Revised (stratigraphic)
**Age justification**	Radiometric dating of the underlying volcaniclastic material suggests a Middle Eocene age for the Messel Formation.
**Reference (fossil relationships)**	Collinson *et al.* (2012)

**Node assignment method**	Intuitive or unspecified (trusted source)
**Reconciliation method**	Molecular tree only
**Min. age (Mya)**	41.2
**Calibration**	stem *Ailanthus* + *Picrasma*
**Node justification**	These specimens belong to a widespread morphospecies which is also known from North America and Asia (Corbett and Manchester 2004). Comparisons with extant species indicate the closest similarity with *Ailanthus altissima* of China (Collinson *et al*. 2012). We accept the assignment of these specimens to *Ailanthus* and use them conservatively to calibrate the stem node of *Ailanthus* and *Picrasma*.

**Calibration no.**	C20
**Family**	Meliaceae
**Full taxon name**	†*Cedrela* sp.
**Reference (first description)**	Hickey & Hodges (1975)
**Reference (latest description)**	
**Organs**	Leaf
**Specimens**	U.S. National Museum specimen 208538
**Locality**	Dubois, Wyoming
**Formation**	Wind River Formation
**Country**	USA
**Reference (age)**	Fan *et al*. (2011)
**Oldest stratum**	Eocene
**Age quality**	Revised (radioisotropic)
**Age justification**	Petrographic, detrital zircon U-Pb geochronology, and paleocurrent analyses date the Wind River Range at ca. 53–51 Ma.
**Reference (fossil relationships)**	Hickey & Hodges (1975)
**Node assignment method**	Intuitive or unspecified (trusted source)
**Reconciliation method**	Pre-molecular era (before 1990)
**Min. age (Mya)**	51
**Calibration**	crown Cedreloideae
**Node justification**	The combination of entire-margined, pinnately compound leaves with an indeterminate leaflet arrangement, angular brochidodromous arches with an outer secondary branch merging with prominent looped marginal venation is restricted to the family Meliaceae. Within extant Meliaceae brochidodromous secondaries are common in *Swietenia* and relatively rare in the closely related genus *Cedrela*. However, only *Cedrela* shows the same pattern of intercostal venation (Hickey & Hodges 1975). Given the similarity of this leaf to *Cedrela* and *Swietenia*, we have chosen to use this fossil as the minimum crown age for Cedreloideae in Meliaceae.

**Calibration no.**	C21
**Family**	Meliaceae
**Full taxon name**	†*Cedrela merrilli* (Chaney) Brown
**Reference (first description)**	Meyer & Manchester (1997)
**Reference (latest description)**	Manchester & McIntosh (2007)
**Organs**	Leaf, seed

**Specimens**	UF278-50012
**Locality**	Crooked River Valley, Post, Oregon
**Formation**	John Day Formation
**Country**	USA
**Reference (age)**	Manchester & McIntosh (2007)
**Oldest stratum**	Late Eocene
**Age quality**	Revised (radioisotropic)
**Age justification**	40Ar/39Ar radioisotopic dating gives the formation a date of 36.21 ± 0.26 Ma.
**Reference (fossil relationships)**	Meyer & Manchester (1997)
**Node assignment method**	Intuitive or unspecified (trusted source)
**Reconciliation method**	Molecular tree only
**Min. age (Mya)**	36.21
**Calibration**	stem *Cedrela* + *Toona*
**Node justification**	The five locules and numerous imbricately positioned winged seeds of this fruit aligns this fossil with the extant Meliaceae genera of *Cedrela* and *Toona* (Manchester & McIntosh, 2007; Meyer & Manchester, 1997). Although the authors suggest the elongated shape is more consistent with *Cedrela*, we take a conservative approach and use the fossil to calibrate the stem node of *Cedrela* + *Toona*.

**Calibration no.**	C22
**Family**	Meliaceae
**Full taxon name**	†*Swietenia miocenica* Castañeda-Posadas & Cevallos-Ferriz
**Reference (first description)**	Castañeda-Posadas & Cevallos-Ferriz (2007)
**Reference (latest description)**	
**Organs**	Flower
**Specimens**	Holotype: Collection of the Museo de Paleontologia Eliseo Palacios Aguilera, sample no. 6114, Tuxtla Gutierrez, Chiapas
**Locality**	Simojovel de Allende, Chiapas
**Formation**	Simojovel Group
**Country**	Mexico
**Reference (age)**	Castañeda-Posadas & Cevallos-Ferriz (2007)
**Oldest stratum**	Late Oligocene
**Age quality**	Revised (stratigraphic)
**Age justification**	Foraminifera evidence suggest an age of 22.5–26.0 Ma (Early Miocene–Late Oligocene) for the group.
**Reference (fossil relationships)**	Castañeda-Posadas & Cevallos-Ferriz (2007)
**Node assignment method**	Apomorphy-based (apomorphies unlisted or tested)
**Reconciliation method**	Molecular tree only
**Min. age (Mya)**	22.5
**Calibration**	stem *Swietenia*
**Node justification**	The complete fusion of the staminal tube, 5-mery, contorted insertion of floral segments and the presence of ciliolated petals and sepals clearly place this fossil flower in Meliaceae. It is most similar to the genus *Swietenia* based on the cylindrical to urceolated staminal tube with a spiny or teethed tip and exposed anthers alternating with

the spines (Castañeda-Posadas & Cevallos-Ferriz 2007). We accept the assignment of this fossil to *Swietenia* and use it to calibrate the minimum age for the stem of the genus.

**Calibration no.**	C23
**Family**	Meliaceae
**Full taxon name**	†*Manchestercarpa vancouverensis* Atkinson
**Reference (first description)**	Atkinson (2020)
**Reference (latest description)**	
**Organs**	Fruit, seed
**Specimens**	Holotype: SH790 B1 bot
**Locality**	Shelter Point, Vancouver Island, British Columbia
**Formation**	Spray Formation
**Country**	Canada
**Reference (age)**	Ward *et al*. (2012)
**Oldest stratum**	Middle Campanian
**Age quality**	Unrevised (recent source)
**Age justification**	Stratigraphic and magnetochrons C33n and C33r of the global polarity time scale suggest an age range for Shelter Point of 79–72 Ma.
**Reference (fossil relationships)**	Atkinson (2020)
**Node assignment method**	Phylogenetic analysis
**Reconciliation method**	Combined morphological and molecular analysis (total evidence)
**Min. age (Mya)**	72.1
**Calibration**	crown Meliaceae
**Node justification**	In the total-evidence analysis of Atkinson (2020), *Manchestercarpa vancouverensis* was recovered within the Melioideae in a clade consisting of *Melia azedarach* L. and *Melia yakimaensis* Pigg, DeVore, Benedict & Creekmore. This clade characterised by a drupaceous fruit morphology. The author suggests that the close relationship of *Manchestercarpa* with *Melia* indicates that the fossil represents a crown member of the tribe Melieae. Given the rigor of the analysis, we accept the assignment of *Manchestercarpa* and use it to calibrate the crown of Meliaceae.

**Calibration no.**	C24
**Family**	Rutaceae
**Full taxon name**	†*Rutaspermum biornatum* Knobloch & Mai
**Reference (first description)**	Knobloch & Mai (1986)
**Reference (latest description)**	
**Organs**	Seed
**Specimens**	Holotype: MMG (9231)
**Locality**	Walbeck, graue Tone
**Formation**	
**Country**	Germany
**Reference (age)**	Magallón *et al*. (2015)
**Oldest stratum**	Maastrichtian
**Age quality**	Unrevised (old source < 2000)

**Age justification**	Stratigraphy.
**Reference (fossil relationships)**	Magallón *et al.* (2015)
**Node assignment method**	Intuitive or unspecified (trusted source)
**Reconciliation method**	Molecular tree only
**Min. age (Mya)**	66
**Calibration**	crown Rutaceae
**Node justification**	The seeds of *Rutaspermum biornatum* are thought to be the oldest reliable representatives of Rutaceae (Gregor 1988). *Rutaspermum* is closely related to the subfamily Zanthoxyleae (=Amyridoideae), particularly *Zanthoxylum* and *Fagara* (Magallón *et al.*, 2015). However, traditional sub-classifications of Rutaceae are not retrieved in our analysis as monophyletic, and therefore this fossil is used to constrain the crown node of Rutaceae.
**Calibration no.**	C25
**Family**	Rutaceae
**Full taxon name**	†*Clausena* sp.
**Reference (first description)**	Pan (2010)
**Reference (latest description)**	
**Organs**	Leaf
**Specimens**	CH41-70, CH41-67, CH41-73, CH41-81
**Locality**	Sublocality CH41, Gondar, Amhara District
**Formation**	Guang River flora
**Country**	Ethiopia
**Reference (age)**	Pan (2010)
**Oldest stratum**	Late Oligocene
**Age quality**	Revised (radioisotropic)
**Age justification**	The age of the Guang River flora is well constrained based on K/Ar radiometric dating methods, 40Ar/39Ar radiometric dating, paleomagnetic reversal stratigraphy and 206Pb/238U analysis. As such the age of 27.23± 0.1 Ma is applied to the flora.
**Reference (fossil relationships)**	Pan (2010)
**Node assignment method**	Apomorphy-based (apomorphies unlisted or tested)
**Reconciliation method**	Molecular tree only
**Min. age (Mya)**	27.23
**Calibration**	stem *Clausena* + *Glycosmis*
**Node justification**	The the crenate margins of the fossil leaflets and presence "rutoid" teeth are thought to be consistent with the family Rutaceae (Pan, 2010). The multicellular uniseriate hairs suggest an affinity with the genus *Clausena* (Pan, 2010). We use this fossil conservatively to constrain the minumum age of *Clausena* + *Glycosmis* to 27.23 Mya.
**Calibration no.**	C26
**Family**	Rutaceae
**Full taxon name**	†*Citrus linczangensis* Xie et al.
**Reference (first description)**	Xie *et al*. (2013)
**Reference (latest description)**	

**Organs**	Leaf
**Specimens**	Holotype: LUMCD090206-022A&B, Institute of Paleontology and Stratigraphy, Lanzhou University, China
**Locality**	Bangmai Village, Lincang City, Yunnan Province
**Formation**	Bangmai Formation
**Country**	China
**Reference (age)**	Xie *et al*. (2013)
**Oldest stratum**	Late Miocene
**Age quality**	Revised (stratigraphic)
**Age justification**	The Bangmai Formation is considered to be of late Miocene age (no later than 11.6 Ma), on the basis of floristic and stratigraphic correlations.
**Reference (fossil relationships)**	
**Node assignment method**	Apomorphy-based (apomorphies unlisted or tested)
**Reconciliation method**	Molecular tree only
**Min. age (Mya)**	11.6
**Calibration**	stem *Citrus*
**Node justification**	Together, the articulated and broadly winged petiole, well-developed intramarginal venation in the lower part of the lamina and the entire margin place this fossil in *Citrus (*Xie *et al*., 2013). This is considered to be the oldest reliable fossil of *Citrus*, and we assign it to the stem of the genus.

**Calibration no.**	C27
**Family**	Rutaceae
**Full taxon name**	†*Ptelea paliuruoides* (Brown) Manchester & O'Leary
**Reference (first description)**	Manchester & O'Leary (2010)
**Reference (latest description)**	
**Organs**	Fruit, seed
**Specimens**	Holotype: "Brown, 1934, Roan Creek, Colorado"
**Locality**	Colorado and Utah
**Formation**	Green River Formation
**Country**	USA
**Reference (age)**	Manchester & O'Leary (2010)
**Oldest stratum**	Middle Eocene
**Age quality**	Revised (radioisotropic)
**Age justification**	The Green River Formation is thought to encompass a ~5 My period between ca. 53.5 and 48.5 Ma, based on the global marine O isotope record.
**Reference (fossil relationships)**	Manchester & O'Leary (2010)
**Node assignment method**	Apomorphy-based (apomorphies unlisted or tested)
**Reconciliation method**	Molecular tree only
**Min. age (Mya)**	48.5
**Calibration**	stem *Ptelea* + *Peltostigma* + *Plethadenia* + *Decazyx* clade
**Node justification**	Evidence of pellucid dots on this fossil are an important feature in common with Rutaceae; along with the locule structure, thin pedicel and hypogynous perianth, this fossil is though to be most closely aligned with *Ptelea* (Manchester & O'Leary, 2010).

We take a conservative approach with this fossil and use it to calibrate the stem node of the clade including *Ptelea* + *Peltostigma* + *Plethadenia* + *Decazyx*.

**Calibration no.**	C28
**Family**	Rutaceae
**Full taxon name**	†*Zanthoxylum* sp.
**Reference (first description)**	Chandler (1961)
**Reference (latest description)**	
**Organs**	Seed
**Specimens**	Holotype: V.29693
**Locality**	Oldhaven Beds, Bishopstone, Herne Bay, Kent
**Formation**	London Clay Flora
**Country**	UK
**Reference (age)**	Chandler (1961)
**Oldest stratum**	Ypresian
**Age quality**	Unrevised (old source < 2000)
**Age justification**	Chandler (1961) places the London Clay Flora in the Ypresian, an age that has apparently not been challenged since.
**Reference (fossil relationships)**	Chandler (1961)
**Node assignment method**	Intuitive or unspecified (trusted source)
**Reconciliation method**	Pre-molecular era (before 1990)
**Min. age (Mya)**	47.8
**Calibration**	stem *Zanthoxylum*
**Node justification**	Seed has morphological affinities with *Zanthoxylum*. Although there are no extant species in Europe, *Zanthoxylum* has a rich and reliable fossil record, and the oldest fossils are from Early Eocene sediments in England (Chandler 1961; Gregor 1989). We therefore accept the assignment of this fossil to *Zanthoxylum* and use it to calibrate the minimum age of the stem node of the genus.

**Calibration no.**	C29
**Family**	Rutaceae
**Full taxon name**	*†Euodia costata* (Chandler) Tiffney
**Reference (first description)**	Tiffney (1981)
**Reference (latest description)**	
**Organs**	Seed
**Specimens**	Holotype: V 20062, British Museum (Natural History)
**Locality**	Central and south London and north Kent
**Formation**	Reading Formation
**Country**	UK
**Reference (age)**	Aldiss (2014)
**Oldest stratum**	Latest Paleocene (Thanetian)
**Age quality**	Revised (radioisotropic)
**Age justification**	The Reading Formation is placed in the Early Eocene (earliest Ypresian), possibly including the latest Paleocene (Thanetian), by reference to magnetostratigraphy and stable isotope stratigraphy.

**Reference (fossil relationships)**	Tiffney (1981)
**Node assignment method**	Intuitive or unspecified (trusted source)
**Reconciliation method**	Molecular tree only
**Min. age (Mya)**	56
**Calibration**	crown Rutoideae clade
**Node justification**	This fossil was originally described as *Phellodendron costatum* Chandler and was later transferred to *Euodia* by Tiffney (1981). Since then, many extant species have been transferred to *Tetradium* and *Melicope*, and the species most similar to the fossil *Euodia costata* was transferred to *Tetradium*. Pending revision of the fossil in light of modern taxonomy, we take a conservative approach with this fossil and assign it to calibrate the crown node of the Rutoideae clade, which contains *Euodia, Tetradium* and *Phellodendron*.
**Calibration no.**	C30
**Family**	Rutaceae
**Full taxon name**	†*Vepris* sp.
**Reference (first description)**	Pan (2010)
**Reference (latest description)**	
**Organs**	Leaf
**Specimens**	CH41-78 and CH41-99
**Locality**	Sublocality CH41, Gondar, Amhara District
**Formation**	Guang River flora
**Country**	Ethiopia
**Reference (age)**	Pan (2010)
**Oldest stratum**	Late Oligocene
**Age quality**	Revised (radioisotropic)
**Age justification**	The age of the Guang River flora is well constrained based on K/Ar radiometric dating methods, 40Ar/39Ar radiometric dating, paleomagnetic reversal stratigraphy and 206Pb/238U analysis. As such the age of 27.23± 0.1 Ma is applied to the flora.
**Reference (fossil relationships)**	Pan (2010)
**Node assignment method**	Apomorphy-based (apomorphies unlisted or tested)
**Reconciliation method**	Molecular tree only
**Min. age (Mya)**	27.23
**Calibration**	stem *Vepris*
**Node justification**	This fossil was assigned to the genus *Vepris* based on leaflet morphology and venation, stomatal structure, petiolule length, epidermis indumentum and cell wall structure (Pan, 2010). We accept this assignment and use this fossil to calibrate the minimum age for the stem of *Vepris*.
