# Supplementary material for: Phylogenomic analyses of Sapindales support new family relationships, rapid Mid-Cretaceous Hothouse diversification, and heterogeneous histories of gene duplication
Source: Front Plant Sci. 2023 Mar 7;14:1063174. doi: 10.3389/fpls.2023.1063174 (PMC10028101; doi:10.3389/fpls.2023.1063174)
Supplement: Supplementary Material 1 — List of ingroup and outgroup samples included in the genus-level phylogenetic analysis of Sapindales and their NCBI SRA accession numbers. [file DataSheet_1.zip › Supplementary Material/Supplementary Material 6.pdf]

Supplementary file 6

Table 1 Ages and 95% HPD intervals for major clades in Sapindales under different dating schemes. 'C.' denotes crown node, 'S.' denotes stem node. KAB refers to major Sapindales clade with Kirkiaceae, Anacardiaceae and Burseraceae; SRM refers to major Sapindales clade with Simaroubaceae, Rutaceae and Meliaceae.

	**CC-complete**	**RC-complete**	**UC-complete**	**Sensitivity analysis: fossil prior distribution**	**Sensitivity analysis: tree prior**
Root prior	*CC-complete (140.33-144.29), uniform*	*RC-complete (143.91-147.94), uniform*	*UC-complete (212.25-221.02) uniform*	*RC-complete (143.91-147.94), uniform*	*RC-complete (143.91-147.94), uniform*
Tree prior	*Birth-Death*	*Birth-Death*	*Birth-Death*	*Birth-Death*	*Yule*
Internal fossil priors	*Uniform*	*Uniform*	*Uniform*	*Log-normal*	*Uniform*
**Stem Sapindales**	128.21 (122.44-134.22)	131.23 (124.45-137.24)	186.48 (174.35-199.53)	130.8 (124.8-136.93)	131.24 (124.92-137.5)
**Crown Sapindales**	121.12 (114.22-127.65)	124.05 (117.28-130.74)	172.4 (158.72-186.91)	123.51 (116.94-130.19)	124.15 (117.58-130.6)
**Stem Biebersteiniaceae + Sapindaceae**	119.23 (112.24-126.12)	122.09 (114.88-128.7)	168.54 (153.04-181.85)	121.55 (114.92-128.47)	122.22 (115.4-128.74)
**Crown KAB+SRM**	115.67 (108.21-122.64)	118.3 (110.58-125.34)	162.42 (146.29-176.38)	117.81 (110.3-124.84)	118.51 (111.24-125.49)
**Stem Simaroubaceae**	105.81 (97.03-114.77)	108.43 (100.01-117.25)	145.28 (127.88-160.84)	107.99 (99.06-116.99)	108.22 (99.54-117.15)
**Stem Meliaceae/Stem Rutaceae**	101.98 (92.29-111.22)	104.68 (96.28-114.03)	139.43 (123.01-156.98)	104.11 (94.78-112.44)	104.35 (95.86-114.04)
**Crown Rutaceae**	94.47 (84.28-104.64)	96.87 (87.21-106.66)	127.63 (110.9-144.81)	96.21 (87.22-105.33)	96.37 (86.17-105.45)
**Crown Meliaceae**	83.19 (72.14-94.65)	86.27 (72.27-97.74)	109.25 (89.07-131.52)	86.89 (76.24-98.16)	85.18 (72.12-97.05)
**Crown Simaroubaceae**	80.81 (61.98-98.91)	83.14 (65.67-101.82)	109.37 (84.98-133.96)	81.8 (63.18-99.5)	83.33 (64.57-102.09)
**Stem Kirkiaceae**	107.91 (98.87-117.1)	109.93 (100.09-119.42)	148.39 (131.07-164.98)	109.17 (99.87-118.62)	110.49 (100.07-119.76)
**Stem Burseraceae/Stem Anacardiaceae**	99.15 (89.69-108.79)	100.65 (89.15-111.36)	133.77 (115.68-150.97)	99.56 (89.42-109.45)	101.07 (89.58-111.55)
**Crown Anacardiaceae**	87.09 (76.28-97.13)	88.33 (77.21-100.08)	113.74 (95.64-131.35)	86.11 (74.15-98.61)	87.35 (73.4-98.9)
**Crown Burseraceae**	83.38 (69.97-96.44)	84.54 (70.93-99.27)	107.92 (84.02-130.29)	84.84 (72.14-96.94)	84.83 (71.53-99.33)
**Crown Kirkiaceae**	1.86 (0.22-4.32)	1.96 (0.21-4.69)	2.23 (0.28-5.25)	1.9 (0.19-4.5)	1.88 (0.18-4.54)
**Stem Sapindaceae/Stem Biebersteiniaceae**	114.08 (105.41-121.82)	116.72 (107.73-124.43)	158.16 (141.11-174.54)	116.23 (107.57-124.02)	116.82 (108.15-124.7)
**Crown Sapindaceae**	102.22 (93.61-111.15)	103.93 (94.68-112.9)	134.58 (117.09-150.8)	104.08 (94.27-113.01)	104.87 (95.78-113.53)
**Crown Nitrariaceae**	73.04 (48.8-102.04)	72.27 (48.08-101.78)	102.83 (63.26-144.95)	71.09 (46.97-97.14)	70.24 (46.65-100.24)

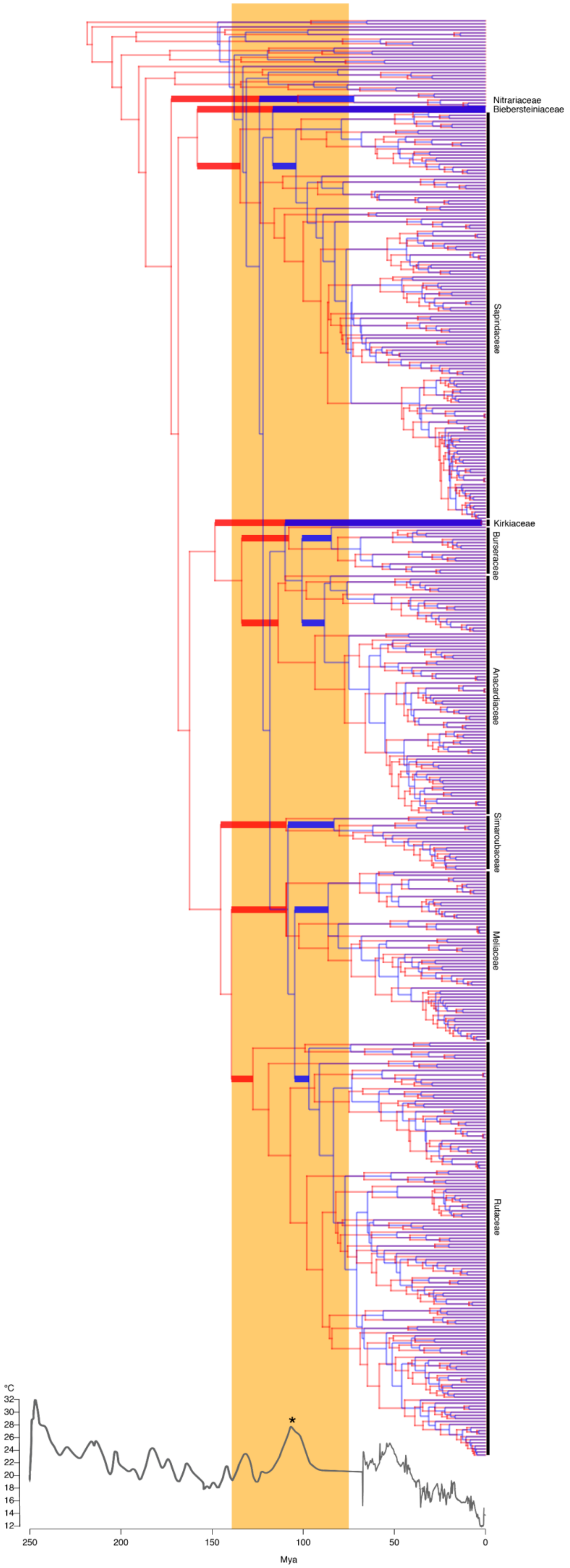

Nitrariaceae
Biebersteiniaceae
Sapindaceae
Kirkiaceae
Burseraceae
Anacardiaceae
Simaroubaceae
Meliaceae
Rutaceae
°C
32
30
28
26
24
22
20
18
16
14
12
*
250
200
150
100
50
0
Mya

Figure 1 Comparison of chronograms estimated under a young-angiosperm (RC-complete; blue) and old-angiosperm (UC-complete; red) scenario. Thick lines mark the stem branch of Sapindales families for each dating analysis. Grey line under chronogram represents global average paleotemperature following Scotese (2021), with orange shading demarcating the Mid-Cretaceous Hothouse and asterisk (*) indicating the Cenomanian–Turonian Thermal Maximum.
