# Supplementary figures and images for: Phylogenomic analyses of Sapindales support new family relationships, rapid Mid-Cretaceous Hothouse diversification, and heterogeneous histories of gene duplication
Source: Front Plant Sci. 2023 Mar 7;14:1063174. doi: 10.3389/fpls.2023.1063174 (PMC10028101; doi:10.3389/fpls.2023.1063174)

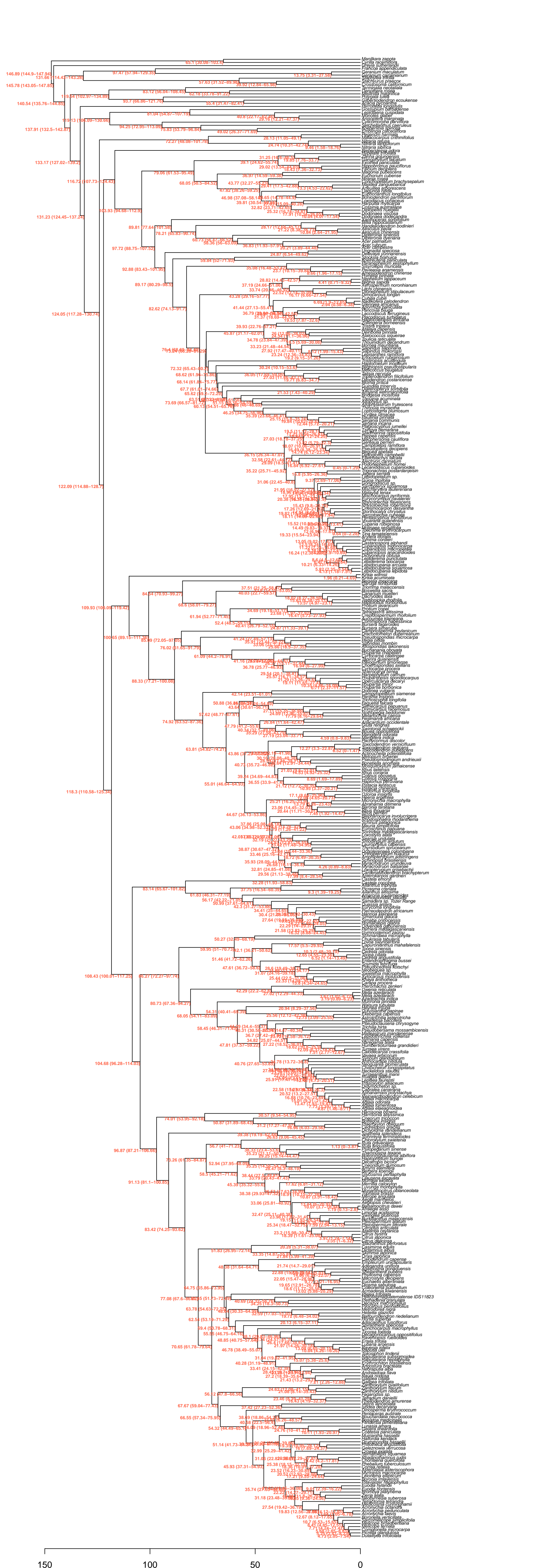

Supplement: Supplementary Material 1 — List of ingroup and outgroup samples included in the genus-level phylogenetic analysis of Sapindales and their NCBI SRA accession numbers. [file DataSheet_1.zip › Supplementary Material/Supplementary Material 8.pdf]

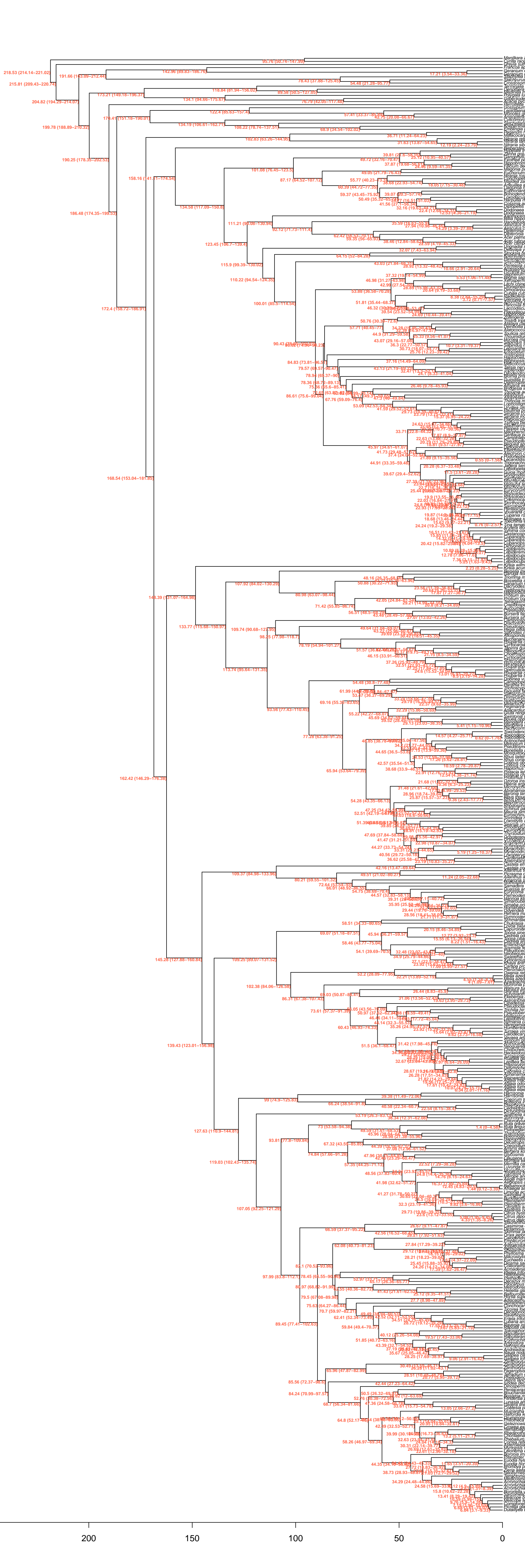

Supplement: Supplementary Material 1 — List of ingroup and outgroup samples included in the genus-level phylogenetic analysis of Sapindales and their NCBI SRA accession numbers. [file DataSheet_1.zip › Supplementary Material/Supplementary Material 9.pdf]

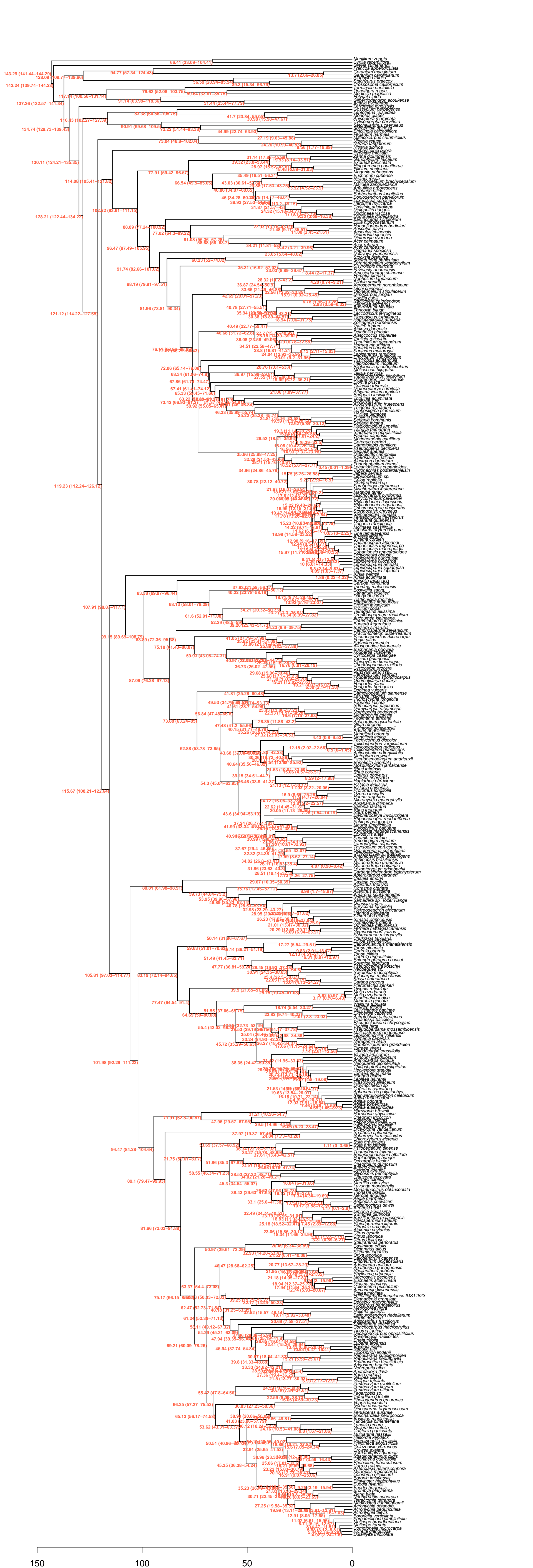

150

100

50

0

Supplement: Supplementary Material 1 — List of ingroup and outgroup samples included in the genus-level phylogenetic analysis of Sapindales and their NCBI SRA accession numbers. [file DataSheet_1.zip › Supplementary Material/Supplementary Material 7.pdf]

Heterozygosity

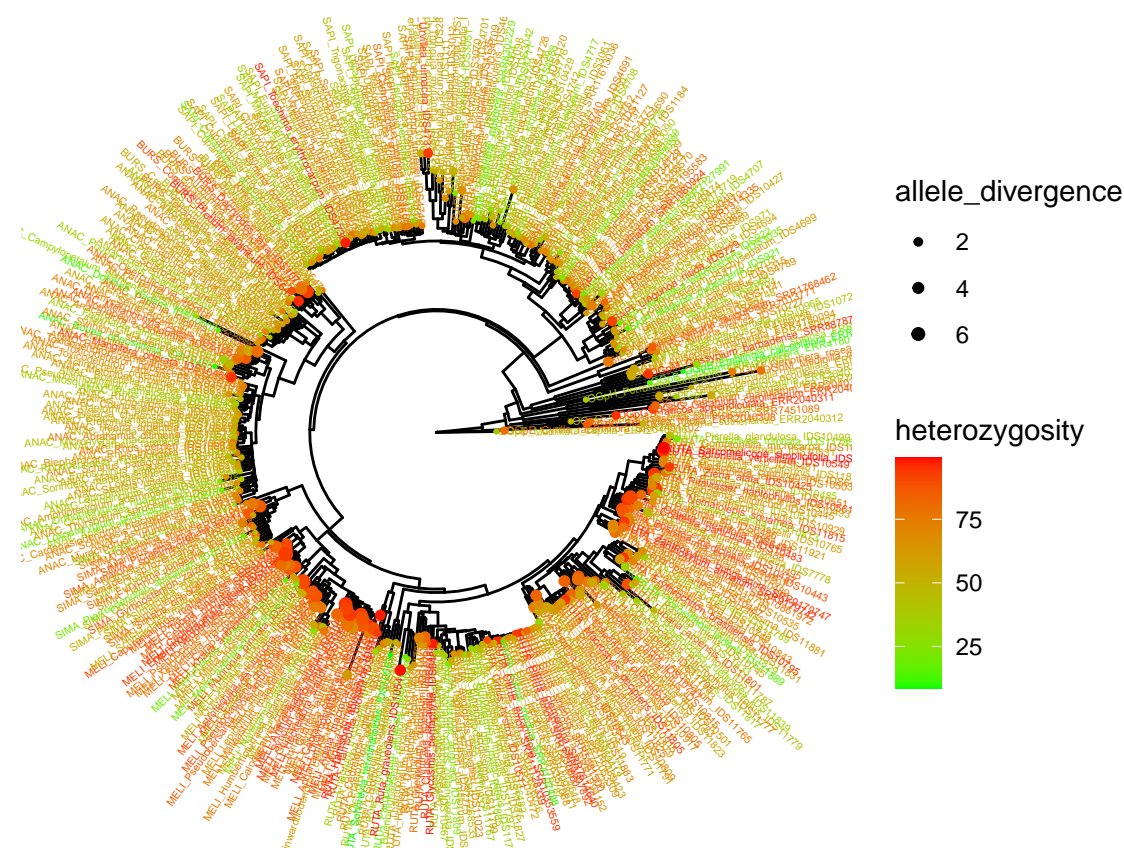

Loci with 0.5% SNPs

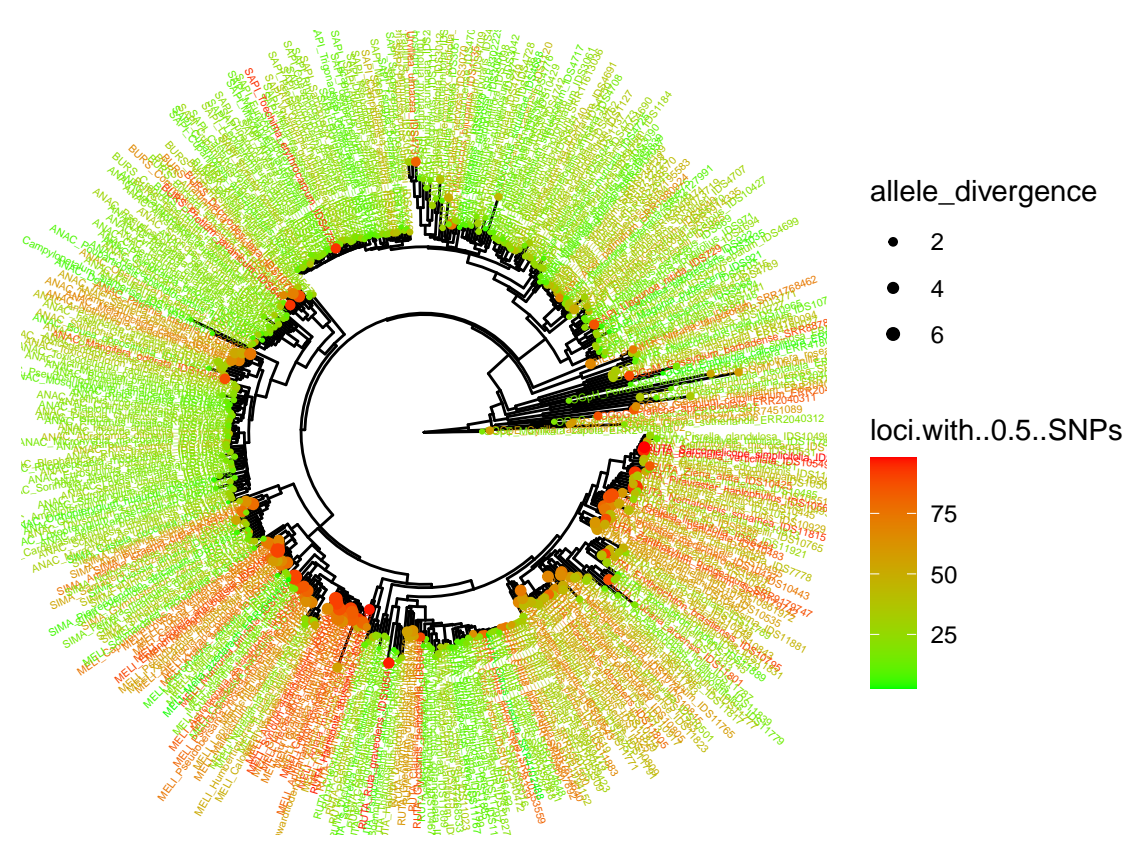

Loci with 1% SNPs

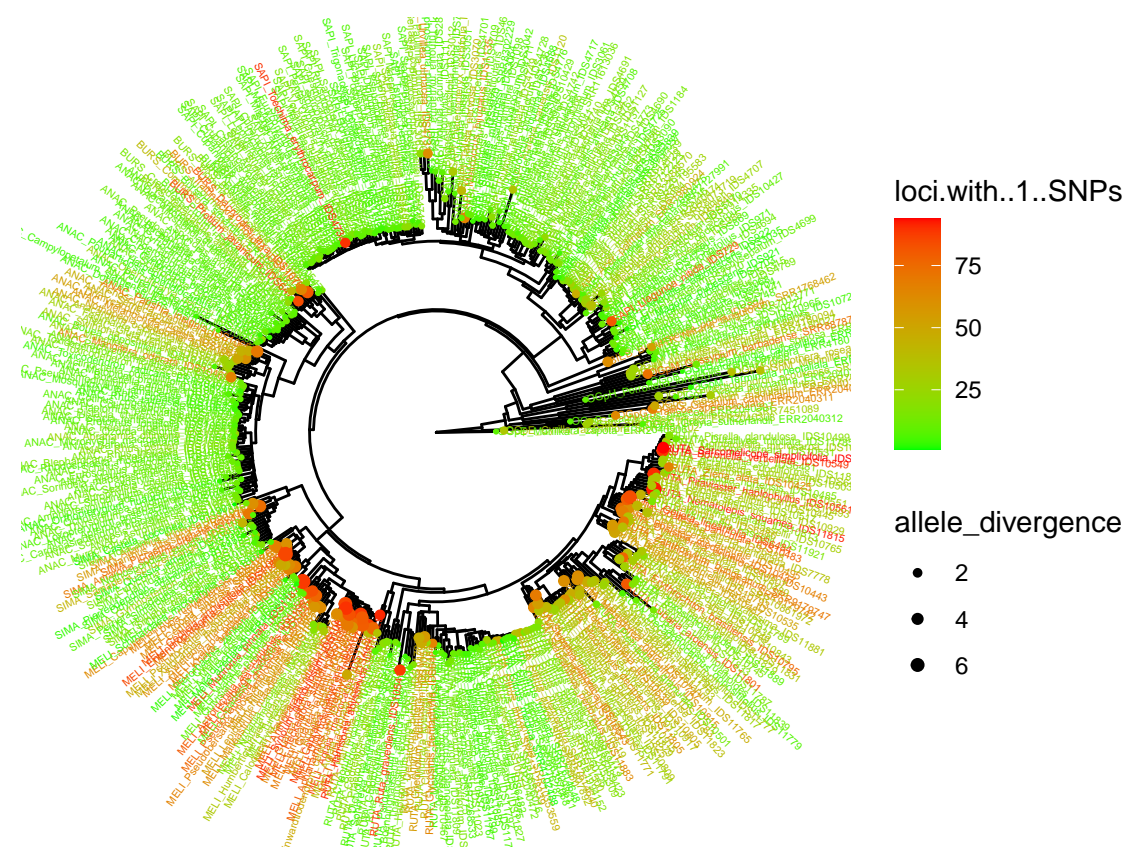

Loci with 2% SNPs

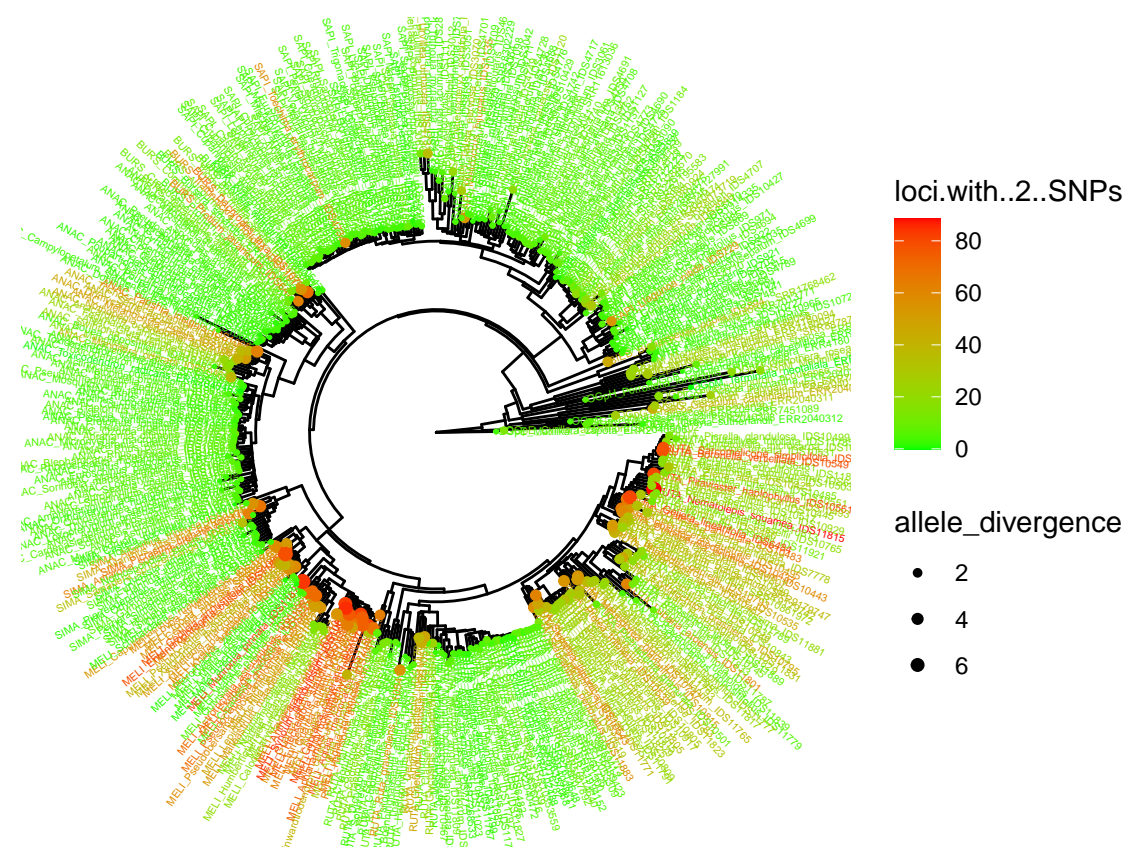

Supplement: Supplementary Material 1 — List of ingroup and outgroup samples included in the genus-level phylogenetic analysis of Sapindales and their NCBI SRA accession numbers. [file DataSheet_1.zip › Supplementary Material/Supplementary Material 4.pdf]

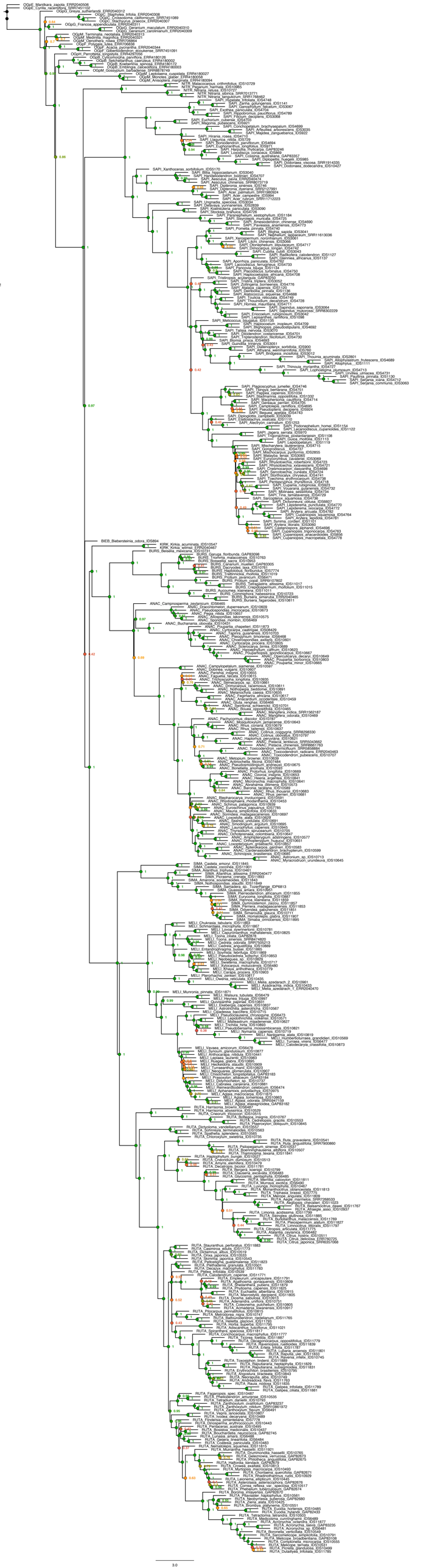

Supplement: Supplementary Material 1 — List of ingroup and outgroup samples included in the genus-level phylogenetic analysis of Sapindales and their NCBI SRA accession numbers. [file DataSheet_1.zip › Supplementary Material/Supplementary Material 5.pdf]

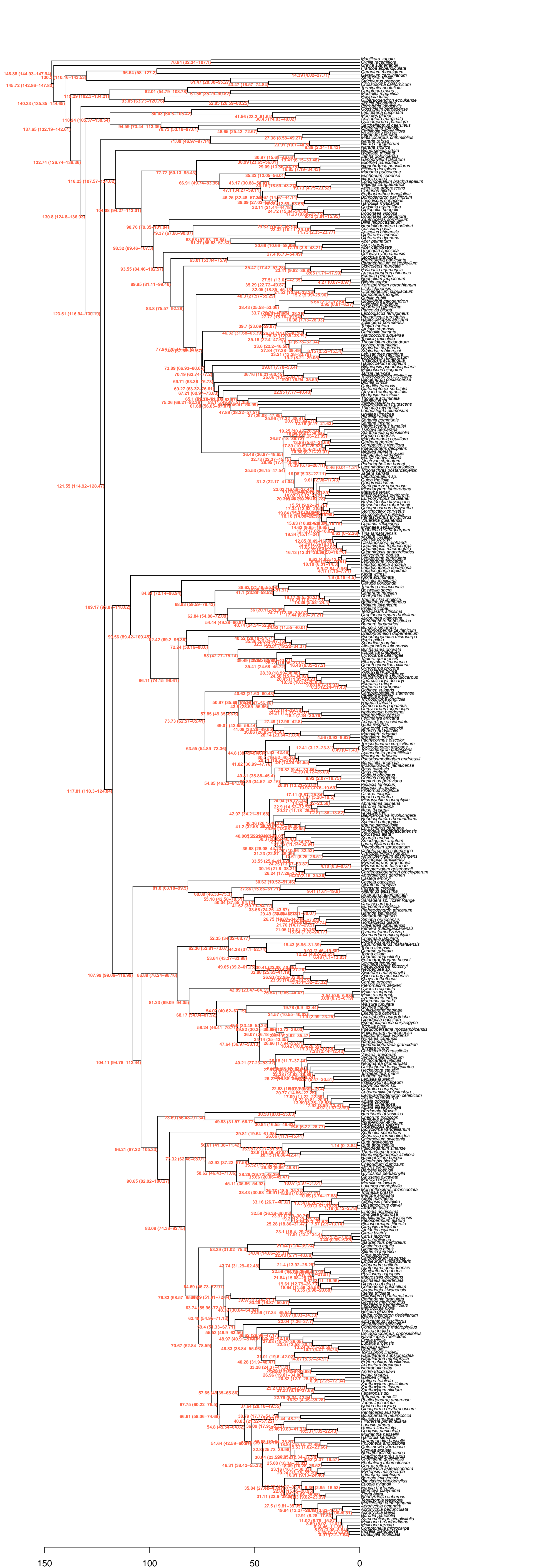

Supplement: Supplementary Material 1 — List of ingroup and outgroup samples included in the genus-level phylogenetic analysis of Sapindales and their NCBI SRA accession numbers. [file DataSheet_1.zip › Supplementary Material/Supplementary Material 11.pdf]

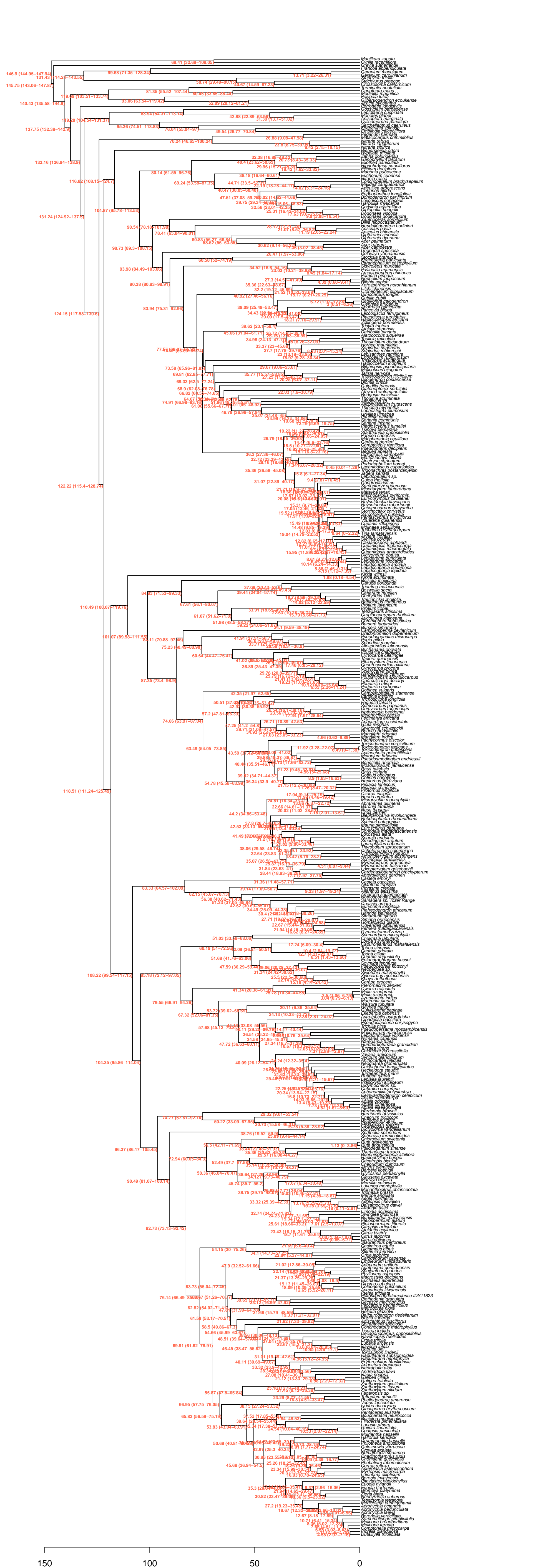

Supplement: Supplementary Material 1 — List of ingroup and outgroup samples included in the genus-level phylogenetic analysis of Sapindales and their NCBI SRA accession numbers. [file DataSheet_1.zip › Supplementary Material/Supplementary Material 10.pdf]
